# Advances in RNA Labeling with Trifluoromethyl Groups

**DOI:** 10.1002/chem.202302220

**Published:** 2023-09-20

**Authors:** Clemens Eichler, Maximilian Himmelstoß, Raphael Plangger, Leonie I. Weber, Markus Hartl, Christoph Kreutz, Ronald Micura

**Affiliations:** ^1^ Institute of Organic Chemistry Center for Molecular Biosciences Innsbruck (CMBI) University of Innsbruck Innrain 80–82 6020 Innsbruck Austria; ^2^ Institute of Biochemistry Center for Molecular Biosciences Innsbruck (CMBI) University of Innsbruck Innrain 80–82 6020 Innsbruck Austria

**Keywords:** ^19^F NMR spectroscopy, nucleoside modifications, oligonucleotides, RNA solid-phase synthesis, trifluoromethyl

## Abstract

Fluorine labeling of ribonucleic acids (RNA) in conjunction with ^19^F NMR spectroscopy has emerged as a powerful strategy for spectroscopic analysis of RNA structure and dynamics, and RNA‐ligand interactions. This study presents the first syntheses of 2′‐OCF_3_ guanosine and uridine phosphoramidites, their incorporation into oligoribonucleotides by solid‐phase synthesis and a comprehensive study of their properties. NMR spectroscopic analysis showed that the 2′‐OCF_3_ modification is associated with preferential C2′‐endo conformation of the U and G ribose in single‐stranded RNA. When paired to the complementary strand, slight destabilization of the duplex caused by the modification was revealed by UV melting curve analysis. Moreover, the power of the 2′‐OCF_3_ label for NMR spectroscopy is demonstrated by dissecting RNA pseudoknot folding and its binding to a small molecule. Furthermore, the 2′‐OCF_3_ modification has potential for applications in therapeutic oligonucleotides. To this end, three 2′‐OCF_3_ modified siRNAs were tested in silencing of the *BASP1* gene which indicated enhanced performance for one of them. Importantly, together with earlier work, the present study completes the set of 2′‐OCF_3_ nucleoside phosphoramidites to all four standard nucleobases (A, U, C, G) and hence enables applications that utilize the favorable properties of the 2′‐OCF_3_ group without any restrictions in placing the modification into the RNA target sequence.

## Graphical abstract

**Synthetic RNA with novel fluorine labeling patterns** is presented. The study completes the set of 2′‐OCF_3_ nucleoside phosphoramidites to all four standard nucleobases and enables advanced NMR spectroscopic and cellular applications that utilize the favorable properties of the 2′‐OCF_3_ group without any RNA sequence restrictions.

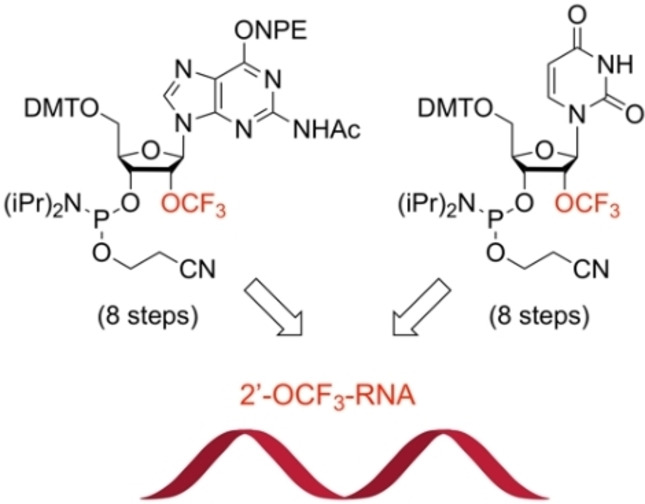

## Introduction

Among the multiple chemical and biophysical approaches to gain insights into RNA structural dynamics and RNA interactions with proteins, other nucleic acids, or small molecules, the use of fluorine labeled RNA combined with ^19^F NMR spectroscopy has attracted significant interest in recent years.[^1^, ^2^, ^3^, ^4^, ^5^, ^6^, ^7^, ^8^, ^9^, ^10^, ^11^, ^12^, ^13^, ^14^, ^15^, ^16^, ^17^, ^18^, ^19^, ^20^, ^21^, ^22^, ^23^, ^24^, ^25^, ^26^, ^27^, ^28^, ^29^, ^30^, ^31^, ^32^, ^33^, ^34^, ^35^, ^36^, ^37^, ^38^, ^39^, ^40^, ^41^, ^42^, ^43^, ^44^] This is due to the exceptional properties of fluorine which include its 100 % natural abundance and consequent high NMR sensitivity. Moreover, fluorine exhibits a significant chemical shift dispersion, rendering it highly responsive to conformational and environmental changes. Fluorine atoms are hardly encountered in native biomolecular systems which is advantageous to monitor the ^19^F NMR signal in complex substance mixtures, for example in cellular extracts or in small‐molecule ligand libraries. However, on the other hand, the lack of fluorine in biomolecules is a drawback because labeling of the biomolecule with a ^19^F handle is required and this is particularly challenging for RNA.

Recently, we have reported on 2′‐*O*‐trifluoromethyl cytidine and ‐adenosine modified RNA as a remarkable labeling concept for NMR spectroscopic applications.^[45]^ The ribose 2′‐OCF_3_ group has the advantage over the widely used 2′‐SCF_3_ label[^28^, ^29^, ^30^] in that it is less thermodynamically destabilizing when residing in a double helix. This conforms with access to more diverse labeling patterns allowing to address a broader scope of research questions. In the previous study we demonstrated that 2′‐OCF_3_ cytidine and adenosine phosphoramidites are readily incorporated into RNA by solid‐phase RNA synthesis with yields that are similar to phosphoramidites of the four standard nucleosides (A, C, G, U).^[45]^ Likewise, deprotection follows the standard protocol. Both facts make 2′‐OCF_3_ labeled RNA accessible with lengths up to 65 and more nucleotides.^[45]^

The introduction of the CF_3_ label at the 2′‐OH group of the nucleoside is more challenging and admittedly remains an unsolved problem with respect to a high‐yielding synthesis of the building blocks.[^46^, ^47^] Accepting the foreseeable low yields for the actual 2′‐OH into 2′‐OCF_3_ transformation, we set out to expand the set of building blocks toward 2′‐OCF_3_ guanosine and uridine phosphoramidites. This expansion is urgently needed because thus far, the outstanding performance of the OCF_3_ label in NMR spectroscopic approaches has been restricted to adenosine and cytidine labeling patterns in target RNA.^[45]^ Here, we demonstrate how to overcome these restrictions by generating access to the complete set of 2′‐OCF_3_ nucleosides for RNA labeling. Consequently, any RNA sequence with site‐specific 2′‐OCF_3_ modifications can be furnished by solid‐phase synthesis for spectroscopic, biochemical, biomedical, and potentially therapeutic applications.

## Results and Discussion

Traditionally, trifluoromethyl ethers are synthesized de novo under harsh reaction conditions using difficult‐to‐handle chemicals, and requiring pre‐functionalized compounds. These methods are limited in practicality/user friendliness and scope.^[48]^ Conceptually, direct OCF_3_ formation via electrophilic trifluoromethylation of alcohols is the most practically straightforward approach. It is also considered to be more tolerant to diverse functional groups, but unfortunately, it is the least explored approach, with only few reagents known in the literature that are capable of this transformation.^[48]^ For instance, a *O*‐(trifluoromethyl) dibenzofuranium salt was successfully employed for the formation of aryl and alkyl trifluoromethyl ethers,^[49]^ however, the preparation of the reagent is challenging. Later, the use of a hypervalent iodine compound for the trifluoromethylation of primary and secondary alcohols using zinc triflimide was reported.^[50]^ A drawback, however, was the requirement for a large excess of the alcohol component. Further developments recently led to an electrophilic trifluoromethylating reagent that combines the hypervalent iodine motif with a sulfoximine ligand (HYPISUL),^[51]^ allowing for a broader substrate scope for trifluoromethylation of a variety of secondary and biorelevant alcohols featuring various functional groups. This reagent seems promising but broad applicability remains to be demonstrated.

Since the above mentioned approaches for trifluoromethylation did not work out in our hands on nucleosides, we decided to focus on the transformation of ribonucleoside 2′‐*O* methyl xanthates to the corresponding 2′‐OCF_3_ modified counterparts albeit this reaction gives generally low yields.[^47^, ^48^] For guanosine, this path was expected to require nucleobase protection to prevent unintended alkylation during methylation of the xanthogenate using methyliodide. Therefore, *O* ^6^‐(4‐nitrophenyl)ethyl (NPE) together with *N* ^2^‐acyl or *N* ^2^‐amidine protection was envisaged which additionally promised sufficient solubility in organic solvent required for practicable workup and isolation of the trifluoromethylated nucleoside derivative with free 5′ and 3′‐OH groups. In this respect, the xanthogenate approach might also be problematic for uridine, however, if so, we were confident that access to 2′‐OCF_3_ U should be feasible by transformation of 2′‐OCF_3_ cytidine into the corresponding uridine.

### Synthesis of 2′‐OCF_3_ guanosine

**Scheme 1:**
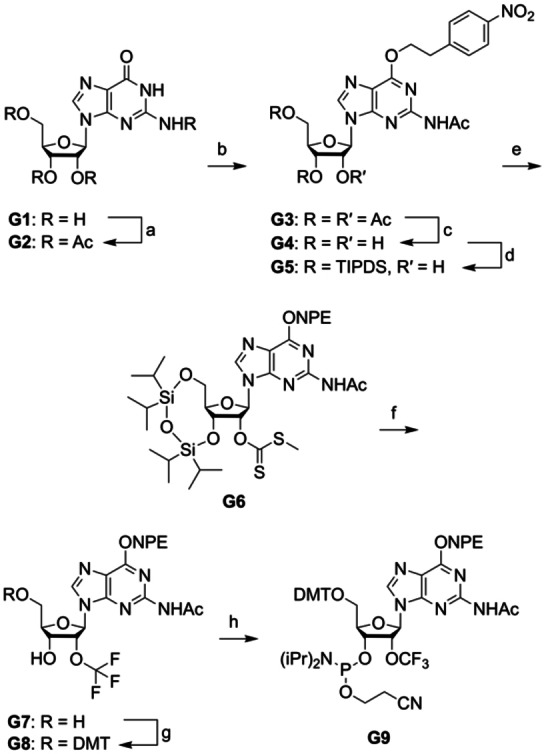
Synthesis of 2′‐OCF_3_ guanosine building block **G9**. Reaction conditions: (a) 6.0 equiv Ac_2_O, 0.5 equiv DMAP, in pyridine, 70 °C, 2 h, 94 %; (b) 1.5 equiv 1‐(4‐nitrophenyl)ethanol, 1.5 equiv PPh_3_, 1.5 equiv diisopropyl azodicarboxylate (DIAD), in THF, 0 °C to room temperature, 16 h, 91 %; (c) in methanol, water and triethylamine, room temperature, 4 h, 81 %; (d) 1.1 equiv TIPDSCl_2_, 2.5 equiv imidazole, in DMF, room temperature, 16 h, 84 %; (e) 1.3 equiv tBuLi, 9.0 equiv CS_2_, 1.3 equiv CH_3_I, in THF, −75 °C to room temperature, 3 h, 70 %; (f) 5.0 equiv NBS, in HF pyridine and CH_2_Cl_2_, −75 °C to 0 °C, 3 h, 16 %; (g) 1.2 equiv DMTCl, 0.3 equiv DMAP, in pyridine, room temperature, 3 h, 83 %; (h) 2.5 equiv 2‐cyanoethyl *N*,*N*‐diisopropylchlorophosphoramidite, 7.5 equiv iPr_2_NEt, 0.5 equiv 1‐methylimidazole, in CH_2_Cl_2_, room temperature, 2 h, 77 %.

The synthetic route to building block **G9** (Scheme 1) started from guanosine **G1**, which was acetylated at its ribose hydroxyls and the exocyclic NH_2_ functionality, providing compound **G2**. After introduction of the *O* ^*6*^‐(4‐nitrophenyl)ethyl group under Mitsunobu conditions furnishing **G3** in high yields, selective removal of hydroxylic acetyl groups was achieved in aqueous methanol‐triethylamine solution. Treatment of **G4** with 1,3‐dichloro‐1,1,3,3‐tetraisopropyldisiloxane (TIPDSCl_2_) selectively installed the Markiewicz protecting group at 3′‐*O* and 5′‐*O* and left the 2′‐OH available for conversion into the 2′‐*O*‐(methylthio)thiocarbonyl functionalized compound **G6** by the use of *tert*‐butyl lithium, carbon disulfide and iodomethane. Transformation to the 2′‐*O*‐trifluoromethyl derivative **G7** was accomplished by treatment with *N*‐bromosuccinimide in hydrofluoric pyridine solution and dichloromethane. Tritylation of the 5′‐OH group proceeded in the presence of 4,4′‐dimethoxytrityl chloride (DMT‐Cl) and dimethylaminopyridine (DMAP) to yield compound **G8**, which was converted to the corresponding phosphoramidite **G9** by reaction with 2‐cyanoethyl *N*,*N*‐diisopropylchlorophosphoramidite. This pathway provides compound **G9** in eight steps with eight chromatographic purifications in 4 % overall yield; in total, 0.7 g of **G9** was obtained in the course of this study.

### Synthesis of 2′‐OCF_3_ uridine

**Scheme 2:**
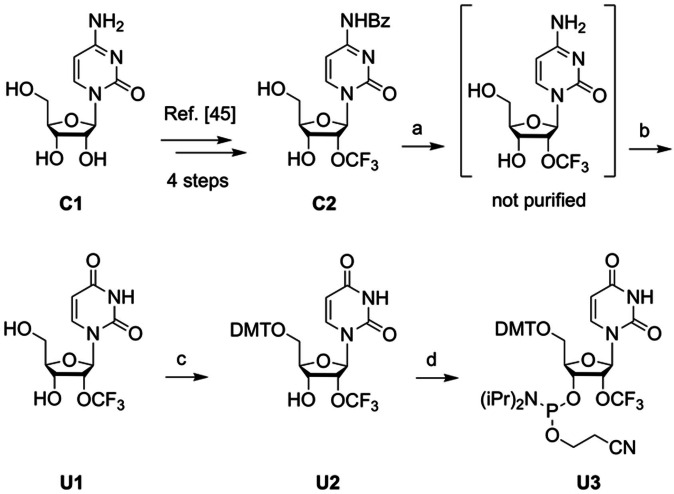
Synthesis of 2′‐OCF_3_ uridine building block **U3**. Reaction conditions: (a) ammonia in methanol, room temperature, 30 min; (b) 1.2 equiv NaNO_2_, 1.2 equiv acetic acid, H_2_O/acetone (2 : 1), room temperature, 2 h, 71 % (over two steps); (c) 1.2 equiv DMTCl, 0.3 equiv DMAP, in pyridine, room temperature, 3 h, 89 %; (h) 2.5 equiv 2‐cyanoethyl *N*,*N*‐diisopropylchlorophosphoramidite, 7.5 equiv iPr_2_NEt, 0.5 equiv 1‐methylimidazole, in CH_2_Cl_2_, room temperature, 4 h, 94 %.

Our diverse and intensive attempts to synthesize 2′‐OCF_3_ modified uridine directly from uridine unfortunately failed. We therefore conceived a path that includes a pyrimidine nucleobase transformation. The synthetic route to building block **U3** (Scheme 2) starts from cytidine **C1** which was transformed into *N* ^4^‐benzoylated 2′‐OCF_3_ cytidine **C2** in four steps in 9.5 % overall yield, following our previously published protocol.^[45]^ Treatment of **C2** with aqueous ammonia in methanol gave the unprotected 2′‐OCF_3_ cytidine which – after evaporation of the solvents – was directly used for the diazotization reaction with sodium nitrate and acetic acid in aqueous solution to yield 2′‐OCF_3_ uridine **U1**. Tritylation of 5′‐OH group was achieved applying 4,4′‐dimethoxytrityl chloride and dimethylaminopyridine to give compound **U2**, which was further converted into the corresponding phosphoramidite **U3** by reaction with 2‐cyanoethyl *N*,*N*‐diisopropylchlorophosphoramidite. This pathway provides compound **U3** in eight steps with eight chromatographic purifications in 6 % overall yield (starting from cytidine); in total, 1.0 g of **U3** was obtained in the course of this study.

### RNA solid‐phase synthesis

**Figure 1:**
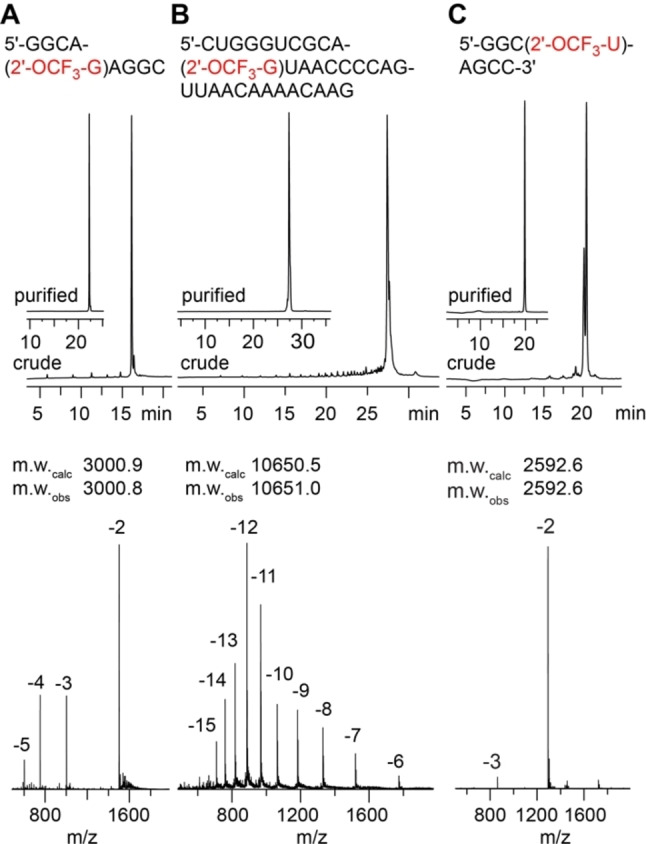
Characterization of 2′‐OCF_3_ guanosine and 2′‐OCF_3_ uridine modified RNA. Anion‐exchange HPLC traces (top) of 9 nt RNA (**A**), 33 nt RNA (**B**), and 8 nt RNA (**C**), and corresponding LC‐ESI mass spectra (bottom). HPLC conditions: Dionex DNAPac PA‐1/200 column (4×250 mm), 80 °C, 1 mL min^−1^, 0–60 % buffer B in 45 min; buffer A: Tris‐HCl (25 mM), 20 % acetonitrile, pH 8.0; buffer B: Tris‐HCl (25 mM), 20 % acetonitrile, NaClO_4_ (0.5 M), pH 8.0. For LC‐ESI MS conditions, see the Supporting Information.

Site‐specific incorporation of 2′‐OCF_3_ modified guanosine and uridine phosphoramidites **G9** and **U3** was achieved by standard RNA solid‐phase synthesis protocols in combination with *N*‐acetylated 2′‐*O*‐[(triisopropylsilyl)oxy]methyl (TOM) phosphoramidites and proceeded with high coupling rates (>98 %) according to trityl monitoring.[^52^, ^53^] Oligonucleotides were cleaved from the solid support and deprotected by treatment with methylamine/ammonia in water (AMA) at 65 °C, followed by reaction with tetra‐*n*‐butylammonium fluoride (TBAF) in tetrahydrofuran. Crude RNAs were desalted by size‐exclusion chromatography and purified on anion‐exchange columns under denaturing conditions (20 % acetonitrile, 80 °C) (Figure 1). To confirm molecular weights, the purified RNAs were analyzed by liquid‐chromatography (LC) electrospray‐ionization (ESI) mass spectrometry (MS) (Figure 1). An overview of the synthesized 2′‐OCF_3_ RNAs is provided in Supporting Table 1.

**Table 1:** Thermodynamic parameters of 2’‐OCF_3_ modified and reference RNAs obtained by UV melting profile analysis.^[a]^

Sequence (5’→3’)	*T_m_* [°C]	Δ*G*°_298_ [kcal mol^−1^]	Δ*H*° [kcal mol^−1^]	Δ*S*° [cal mol^−1^ K^−1^]
GGUCGACC	58.2	−14.5	−78.0	−213
GGUC(2’‐OCF_3_‐G)ACC	50.8	−12.2	−69.4	−192
GUC(2’‐SCF_3_‐G)ACC^[b]^	35.0	−7.8	−72.3	−216
GAAGGGCAACCUUCG	73.3	−7.0	−52.8	−153
GAA(2’‐OCF_3_‐G)‐GGCAACCUUCG	68.5	−6.7	−54.4	−160
GAA(2’‐SCF_3_‐G)‐GGCAACCUUCG^[b]^	57.5	−5.3	−54.7	−166
GGCUAGCC	60.5	−15.3	−80.2	−218
GGC(2’‐OCF_3_‐U)AGCC	54.8	−13.7	−76.2	−210
GAAGGGCAACCUUCG^[c]^	69.9	−7.6	−57.6	−168
GAAGGGCAACC(2’‐OCF_3_‐U)UCG^[c]^	65.3	−6.7	−55.3	−163

[a] Buffer conditions: 10 mM Na_2_HPO_4_, 150 mM NaCl, pH 7.0. Δ*H* and Δ*S* values were obtained by van't Hoff analysis according to references 54 and 55. Errors for Δ*H* and Δ*S*, arising from non‐infinite cooperativity of two‐state transitions and from the assumption of a temperature‐independent enthalpy, are typically 10–15 %. Additional error is introduced when free energies are extrapolated far from melting transitions; errors for Δ*G* are typically 3–5 %. [b] Data reproduced from Ref. [29]. [c] Buffer: Same as [a] but 100 mM NaCl.

### Thermodynamic stability of 2′‐OCF_3_ modified RNA

**Figure 2:**
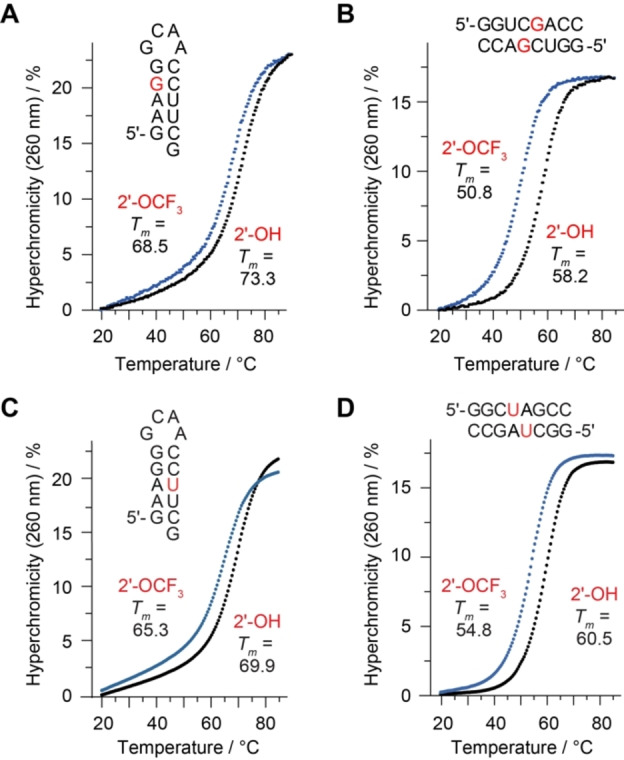
Thermal stabilities of unmodified versus 2′‐OCF_3_ modified oligoribonucleotides. UV‐melting profiles of 2′‐OCF_3_ guanosine containing hairpin (**A**) and self‐complementary duplex (**B**) with the modification located in the base‐pairing region. (**C**, **D**) Same as (**A**, **B)** but with 2′‐OCF_3_ uridine. Conditions: c(RNA)=8 μM (hairpin **A**), 12 μM (palindrome **B**); 10 mM Na_2_HPO_4_, 150 mM NaCl (for **A**, **B**)), 100 mM NaCl (for **C**, **D**), pH 7.0. Nucleotides in red color indicate the positions for 2’‐OCF_3_ modification.

We investigated the impact of a single 2′‐OCF_3_ guanosine modification on RNA pairing stability, determined by UV spectroscopic thermal denaturation studies. For instance, melting profile analysis of the 5′‐GAA(2′‐OCF_3_‐G)G‐GCAA‐CCUUCG hairpin RNA (Table 1, Figure 2A) resulted in a 4.8 °C decrease of the *T_m_* value (*T_m_* 68.5 °C) compared to the unmodified analog (*T_m_* 73.3 °C). When 2′‐OCF_3_‐G resided in the self‐complementary RNA 5′‐GGUC(2′‐OCF_3_‐G)ACC (Figure 2B), the decrease of melting temperature amounted to 7.4 °C (*T_m_* 50.8 °C) compared to the unmodified counterpart (*T_m_* 58.2 °C). This corresponds to a 3.7 °C drop per base pair containing a 2′‐OCF_3_ guanosine.

Likewise, RNA containing 2′‐OCF_3_ uridine were slightly destabilized. This time, we reduced the NaCl concentration from 150 to 100 mM NaCl under otherwise same buffer conditions. Melting profile analysis of the 5′‐GAAGG‐GCAA‐CC(2′‐OCF_3_‐U)UCG hairpin RNA (Figure 2A) resulted in a 4.6 °C decrease of the *T_m_* value (*T_m_* 65.3 °C) compared to the unmodified analog (*T_m_* 69.9 °C). When 2′‐OCF_3_‐U was placed in the self‐complementary RNA 5′‐GGC(2′‐OCF_3_‐U)GACC (Figure 2B), the decrease of melting temperature amounted to 5.7 °C (*T_m_* 54.8 °C) compared to the unmodified counterpart (*T_m_* 60.5 °C). This corresponds to a 2.8 °C drop per base pair containing a 2′‐OCF_3_ uridine.

Taken together, the UV melting study demonstrated that 2′‐OCF_3_ modified RNA is significantly less thermodynamically destabilizing in comparison to the previously reported 2′‐SCF_3_ RNAs (Table 1),^[29]^ It otherwise retains all the advantages for ^19^F NMR spectroscopy attributed to the CF_3_ group, and therefore, a much broader range of applications is foreseeable for 2′‐OCF_3_ RNA.

### 2′‐OCF_3_ ribose conformation

**Figure 3:**
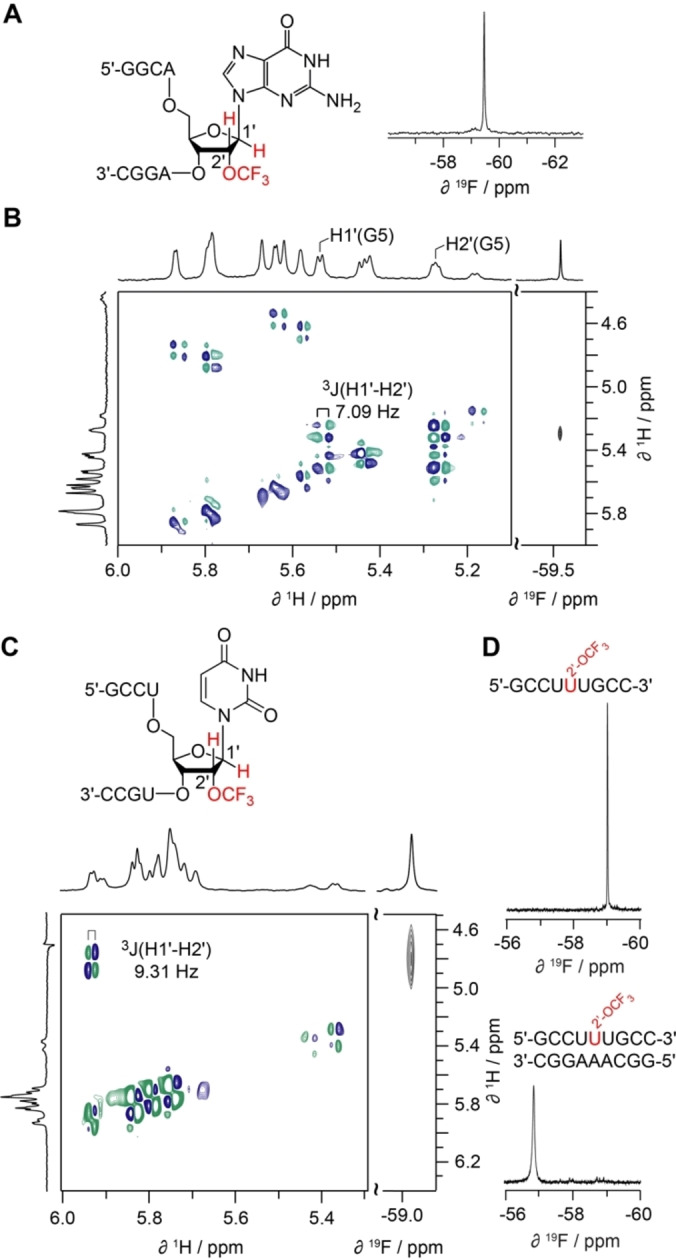
NMR spectroscopic analysis of 2′‐OCF_3_ guanosine and 2′‐OCF_3_ uridine containing RNA. (**A**) ^19^F and (**B**) ^1^H/^1^H DQFCOSY, ^19^F/^1^H HOESY NMR spectra of single‐stranded RNA 5′‐GGCA(2′‐OCF_3_‐G)AGGC; assignment of the H2′ of 2′‐OCF_3_ guanosine moiety was based on the ^19^F/^1^H NOE cross peak; the ^3^ *J* scalar coupling between H2’ and H1’ amounted to 7.1 Hz (71 % C2′ endo). (**C**) ^1^H/^1^H DQFCOSY NMR spectrum of single‐stranded RNA 5′‐GCCU(2′‐OCF_3_‐U)UGCC; the ^3^ *J* scalar coupling between H2′ and H1′ of the 2′‐OCF_3_ uridine moiety amounted to 9.3 Hz (90 % C2′ endo). Conditions: c(RNA)=0.3 mM; 15 mM Na[AsO_2_(CH_3_)_2_] ⋅ 3H_2_O, 25 mM NaCl, 3 mM NaN_3_, in D_2_O, pH 6.5, 298 K.

A major determinant for modified nucleotide helix stability is a label‘s preference to adopt C2′‐endo or C3′‐endo ribose conformation.[^56^, ^57^, ^58^, ^59^] Assuming a simple two state equilibrium between the two sugar puckers, the percental population can be directly calculated from the scalar coupling of H1′ and H2′ of the individual ribose unit. For this purpose, we synthesized the short single‐stranded RNA 5′‐GGCA(2′‐OCF_3_‐G)AGGC (Figure 3A) and assigned the H2′ of the trifluoromethylated guanosine in position 5 in a ^19^F/^1^H NOESY NMR experiment relying on its proximity to the fluorine label. The ^3^ *J* coupling constant to H1′(G5) was then obtained from a 2D ^1^H/^1^H double quantum filtered COSY spectrum (Figure 3B); it amounted to 7.09 Hz which conforms to a C2′‐endo population of ca. 70 %. For 2′‐OCF_3_ uridine in the single stranded RNA 5′‐GCCU(2′‐OCF_3_‐U)UGCC (Figure 3C), the ^3^ *J* coupling constant between H1′ and H2′ of the 2′‐OCF_3_ ribose was determined to be 9.1 Hz which conforms to a C2′‐endo population of 91 %. We mention that only 58 % population for C2′‐endo conformation was measured for 2′‐OCF_3_ adenosine in the single strand 5′‐GGCAG(2′‐OCF_3_‐A)GGC.^[45]^ Taken together, these observations provide evidence that forcing the G and U label into a C3′‐endo ribose pucker, as mandatory for a double helical A‐form RNA, results in a higher energetic penalty than for the 2′‐OCF3 adenosine.

Pairing of 5′‐GCCU(2′‐OCF_3_‐U)UGCC with the complementary RNA strand 5′‐GGCAAAGGC was reflected in a pronounced downfield shift and significant broadening of the ^19^F signal (Figure 3D), indicating reduced conformational (rotational) freedom of the 2′‐OCF_3_ group in the minor groove of the formed double helix.

**Figure 4:**
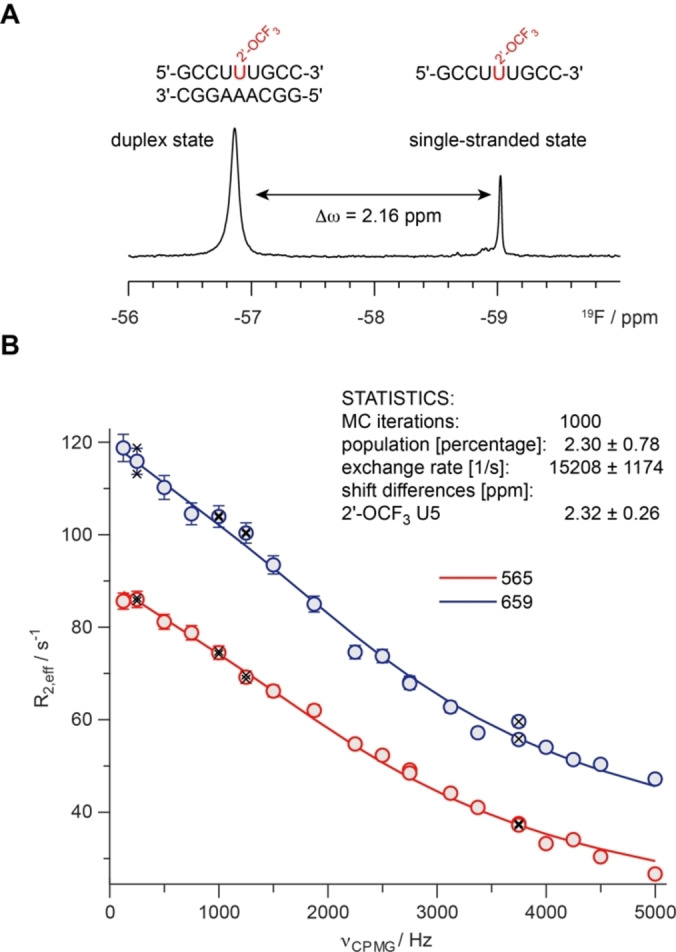
Carr‐Purcell‐Meiboom‐Gill (CPMG) relaxation dispersion (RD) experiment to detect and quantify a potential dynamic process of the 2′‐OCF_3_ modified ribose in single‐stranded vs duplex RNA. (**A**) RNA sequences and ^19^F NMR spectra of a mixture of duplex and single strand in a ratio of 1.0 to 1.2. (**B**) ^19^F‐relaxation dispersion profiles of 2′‐OCF_3_ U5 recorded at 565 and 659 MHz ^19^F‐Larmor frequency. The statistics of the two‐state exchange process are shown as inset (for discussion see main text). R_2_ (transverse relaxation rate), ν_CPMG_ (CPMG field strength). Dots represent experimental data, black crosses repeat experiments and the solid line is the best fit to an intermediate exchange process using the Carver‐Richards equation. MC Monte Carlo iterations for error statistics.

Intrigued by the significant line broadening effect upon duplex formation, we used a Carr‐Purcell‐Meiboom‐Gill (CPMG) relaxation dispersion (RD) experiment to detect and quantify a potential dynamic process on the intermediate chemical shift time scale (Figure 4). For this purpose, we prepared a sample with a slight excess (ca. 20 %) of the single strand carrying the 2′‐OCF_3_‐U label and run a RD experiment with CPMG field strengths up to 5 kHz. A non‐flat dispersion profile was observed for the duplex CF_3_ resonance, whereas for the sharp single stranded resonances no significant dispersion profile was found. The high quality dispersion data could be fit to an intermediate two state exchange process by using the Richard‐Carver equation^[60]^ and an in house written MATLAB script. An excited state population of 2.30±0.78 %, an exchange rate *k*_ex_ (=*k*_forward_+*k*_backward_) of 15.208±1174 s^−1^ and a chemical shift difference of 2.32±0.26 ppm was found. We can rule out an exchange process between single and double stranded state, as no RD profile was observed for the single strand resonance. The single stranded 2′‐OCF_3_‐U populates to ca. 90 % the C2′‐endo state (Figure 3C and D). Correlating the chemical shift difference of the single/double stranded state (Δω 2.16 ppm) to the chemical shift difference between ground and excited state from the RD experiment (Δω 2.32 ppm) supports a sugar pucker equilibrium of the 2′‐OCF_3_‐U in the duplex between the C2′‐endo (excited state, 2.3 %) and the C3′‐endo (ground state, 97.7 %) sugar pucker.

### NMR analysis of RNA small molecule binding

**Figure 5:**
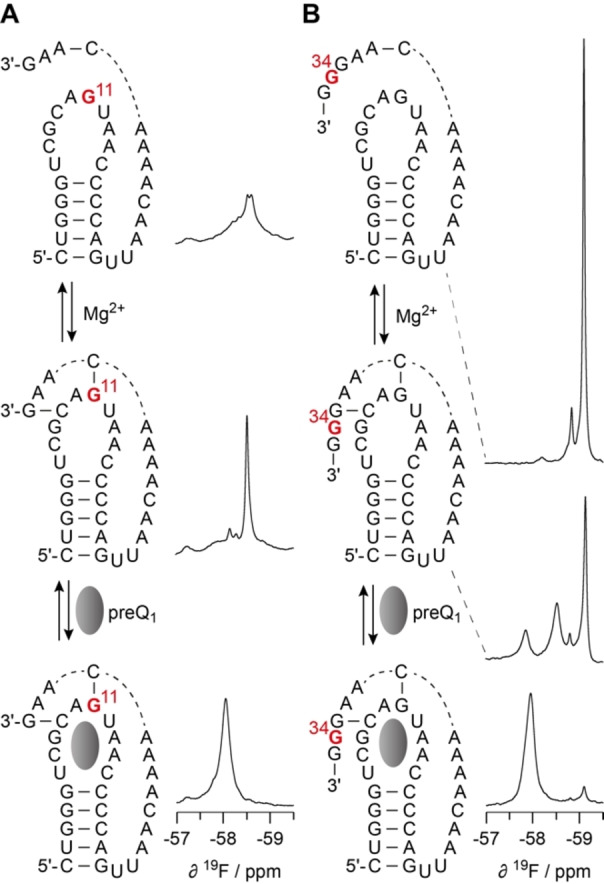
NMR spectroscopic evaluation of Mg^2+^‐induced RNA pseudoknot formation, and subsequent stabilization through binding of a small ligand (*Thermoanaerobacter tengcongensis* preQ_1_ class‐I riboswitch), using individually positioned 2’‐OCF_3_ guanosine labels. ^19^F NMR spectra of preQ_1_ riboswitch model 2’‐OCF_3_ modified at either G11 (**A**) or G34 (**B**). Conditions: c(RNA)=0.3 mM; 15 mM Na[AsO_2_(CH_3_)_2_] ⋅ 3H_2_O, 25 mM NaCl, 3 mM NaN_3_, 10 % D_2_O, pH 6.5, 298 K.

RNA with a single 2′‐OCF_3_ label provides a powerful sensor to monitor RNA folding and RNA interactions with other biomolecules by ^19^F NMR spectroscopy. In this work, we exemplarily applied the 7‐aminomethyl‐deazaguanine (preQ_1_) sensing class‐I riboswitch from *Thermoanaerobacter tengcongensis* (*Tte*)[^61^, ^62^, ^63^, ^64^, ^65^] as model system and tested two positions of guanosine (G11 and G34) for their potential to follow Mg^2+^ induced folding of an RNA pseudoknot and binding of a small molecule (preQ_1_) to this particular aptamer with high (nanomolar) affinity (Figure 5). In aqueous buffer at pH 6.5, the 2′‐OCF_3_‐G11 labeled *Tte* RNA displays a rather broad ^19^F NMR signal group (Figure 5A, top), indicating multiple RNA loop conformations in the intermediate to slow exchange regime. Only when Mg^2+^ is added, a single sharp resonance dominates (Figure 5A, middle) which is consistent with a pre‐organized pseudoknot fold in which G11 is base‐paired with C30 (in accordance with crystallography^62^ and NMR studies[^63^, ^66^]). The observed Mg^2+^ induced pseudoknot folding is also in line with the observation by other methods such as 2APfold[^67^, ^68^] or smFRET spectroscopy.[^67^, ^69^, ^70^] Once the cognate ligand (preQ_1_) is added, the ^19^F signal shifts downfield (Figure 5A, bottom) consistent with Watson‐Crick (WC) base pairing of G11‐C30 (see also Figure 3D for comparison). The increased line width of the ^19^F signal is likely attributed to restricted rotational freedom of the 2′‐OCF_3_ group in the rigid ligand‐RNA complex.

A strength of single label RNA ^19^F NMR analysis as outlined above is the high sensitivity for *local* conformational rearrangements. In this sense, the 2′‐OCF_3_‐G11 label reflects changes in the RNA loop conformation, and additionally, responds to pseudoknot formation through WC base pairing to C30. The dynamics of pseudoknot formation can also be pursued from a complementary perspective, namely the RNA 3′‐tail. Accordingly, the 2′‐OCF_3_‐G34 labeled RNA displays a sharp ^19^F NMR resonance in Mg^2+^ free, aqueous buffer at pH 6.5 (Figure 5B, top) which is consistent with a conformationally flexible unpaired single stranded RNA. When Mg^2+^ is added the fraction of the stem‐loop RNA with dangling 3′‐tail is reduced and two more major ^19^F NMR signals appear (Figure 5B, middle). One of them is assigned to a conformation which closely resembles the final ligand‐bound RNA fold according to the comparable chemical shift values. The other Mg^2+^‐induced conformation likely also reflects a closed (pseudoknotted) conformation but it is structurally more distinct from the final ligand‐RNA complex, consistent with the distinct chemical shifts (Figure 5B, bottom).

In summary, this example demonstrates the power and convenience of singly labeled 2′‐OCF_3_ labeled RNA for the detection of RNA conformational states (including an estimate of the timescale for their exchange) and for the detection of RNA ligand interactions by 1D ^19^F NMR spectroscopy.

### Potential of 2′‐OCF_3_ RNA for RNA interference

As a novel application for the 2′‐OCF_3_ modification, we tested the potential of this modification for gene silencing by small interfering RNA (siRNA). The structural proximity of 2′‐OCF_3_ to 2′‐OCH_3_ makes it a promising candidate for such applications, in particular under the aspect that 2′‐OCH_3_ represents the most frequently encountered modification in clinically approved oligonucleotide therapeutics.^[71]^ Albeit the 2′‐OCF_3_ has a slight destabilizing effect on duplex stability which is not necessarily a disadvantage. Nucleosides with destabilizing effects on Watson‐Crick base pairing are of specific interest for the development of oligonucleotide therapeutics.[^72^, ^73^, ^74^] Most prominent is the unlocked nucleic acid (UNA) missing the covalent bond between C2′ and C3′ of a ribose.^[71]^ UNA modifications facilitate antisense strand selection as the RISC guide, and UNA inserts to the seed region of the siRNA guide strand can significantly reduce off target effects.^[75]^

Here, we intended to explore the performance of 2′‐OCF_3_ for siRNA applications. We employed the model system used previously to knock down the brain acid soluble protein 1 (BASP1) encoding gene by transient siRNA nucleofection in the chicken DF‐1 cell line.^[76]^ Expression of the *BASP1* gene is specifically downregulated by the evolutionary conserved oncoprotein Myc;^[77]^ conversely, the BASP1 protein is an inhibitor of Myc‐induced cell transformation.^[76]^

**Figure 6:**
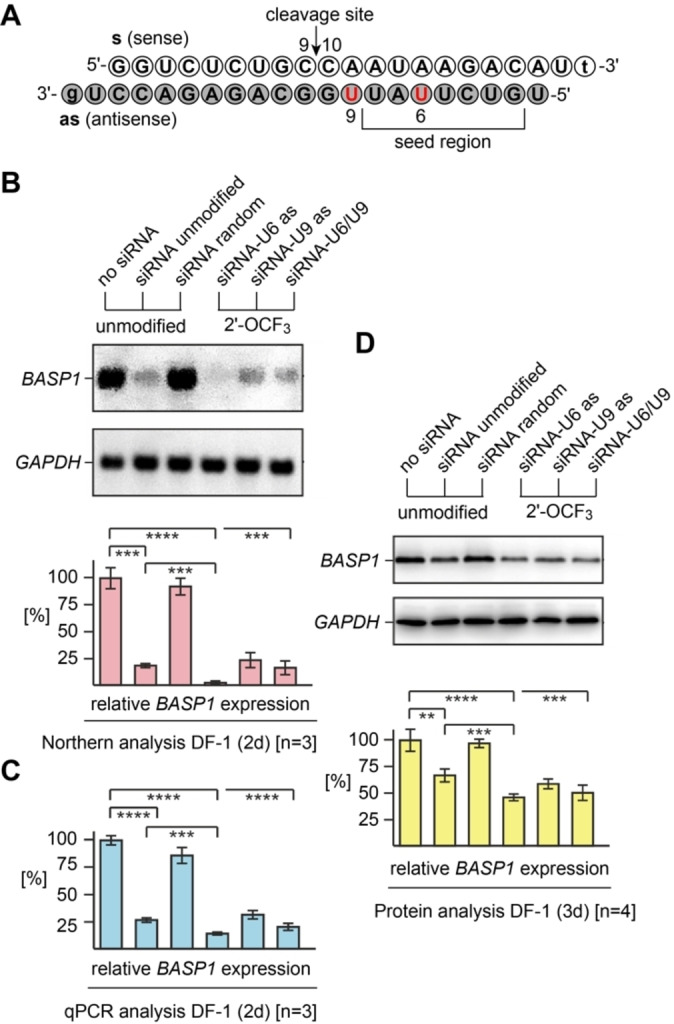
Gene silencing by 2′‐OCF_3_ modified siRNAs. (**A**) Sequence of the brain acid soluble protein 1 gene (*BASP1*)^[76]^ targeting siRNA duplex used in this study; nucleosides in red indicate positions for the modification tested. (**B**) Biological activities of 2’‐OCF3 modified siRNAs, directed against *BASP1* mRNA. Chicken DF‐1 cells grown on 60 mm dishes were transiently nucleofected with 0.24 nmol (∼3.0 μg) aliquots of the individual siRNAs. An equal aliquot of siRNA with a shuffled (random) nucleotide sequence was used as a control. Total RNAs were isolated 2 days after siRNA delivery, and 5 μg of aliquots were analyzed by Northern hybridization using a digoxigenin‐labeled DNA probe specific for the chicken *BASP1* gene, and subsequently with a digoxigenin‐labeled probe specific for the housekeeping chicken *GAPDH* gene. Sizes for the mRNAs are: *BASP1*, 2.0 kb; *GAPDH*, 1.4 kb. The levels (%) of *BASP1* expression were determined using the program ImageQuant TL and are depicted as bars in relation to mock transfections (no siRNA, 100 %). Vertical bars show standard deviations (SD) from independent experiments (n=3). Statistical significance was assessed by using a paired Student *t*‐test (***P<0.001, ****P <0.0001). (**C**) The same as (B) but analyzed by quantitative polymerase chain reaction (qPCR) using each 2.5 ng cDNA template reverse transcribed from total RNA, and primers specific for chicken *BASP1* or *GAPDH*. All siRNAs depicted contain overhangs of 2′‐deoxynucleosides (lower case letters). (**D**) Immunoblot analysis using cell extracts prepared 3 days after siRNA delivery and antibodies specific for the BASP1 or GAPDH proteins. The levels (%) of BASP1 expression were determined using the program ImageQuant TL and are depicted as bars in relation to mock transfections (no siRNA, 100 %). Vertical bars show standard deviations (SD) from independent experiments (n=4). Statistical significance was assessed by using a paired Student *t*‐test (**P<0.01, ***P<0.001, ****P<0.0001).

We synthesized three siRNA duplexes for the *BASP1* target gene with the sequence organization depicted in Figure 6A (Supporting Information, Supporting Table 2). The modifications were placed in the antisense strands, two of the siRNA contained a single modification (U6 as; U9 as), and the third one contained both modifications (U6/U9 as).

Expression of the *BASP1* gene and of its protein product BASP1 were monitored by Northern and qPCR analysis, and by immunoblotting, respectively. The modified siRNAs U9 and U6/9 caused comparable gene silencing as observed for the unmodified reference siRNA. The siRNA U6 – with the modification residing inside the seed region – displayed even slightly increased repression compared to the unmodified siRNA duplex (Figure 6B–D). These results point at the potential of 2′‐OCF_3_ modifications to tailor siRNAs with advanced performance. In particular, our observation for improved repression of the *BASP1* gene with an siRNA carrying the 2′‐OCF_3_ group in the seed region warrants more comprehensive studies along these lines in the future.^[72]^

## Conclusions

Numerous ^19^F labels for NMR spectroscopy of nucleic acids have been developed previously. These include single fluorine labels, such as pyrimidine 5‐F,[^16^, ^17^, ^18^, ^19^, ^20^] ribose 2′‐F[^21^, ^22^, ^23^, ^24^, ^25^] and 4′‐F,^[3]^ as well as trifluoromethyl labels like pyrimidine 5‐CF_3_,^[26]^ guanine 8‐CF_3_,^[31]^ ribose 4′‐C‐3[(4‐trifluoromethyl‐1*H*‐1,2,3‐triazol‐1‐yl)methyl]^27^ and 2′‐SCF_3_.[^28^, ^29^, ^30^] Furthermore, a nine‐fluorine‐atom label in the form of 5‐[4,4,4‐trifluoro‐3,3‐bis(trifluoromethyl)but‐1‐ynyl] 2′‐deoxyuridine has also been utilized.^[4]^ Among these options, the *ribose* trifluoromethyl labels stand out because they meet several important criteria for the practicability of ^19^F NMR approaches at the same time. These are their relatively small size, no need for proton decoupling, high sensitivity, large chemical shift dispersion, and equivalent labeling position for all of the four standard nucleosides. Towards this end, we introduced 2′‐OCF_3_ cytidine and ‐adenosine labeled RNA recently,^[45]^ and demonstrated that their thermodynamic base pairing properties are superior compared to the thus far more established 2′‐trifluoromethyl*thio* RNA labeling concept.[^28^, ^29^, ^30^, ^45^] In the present study, we extend the 2′‐OCF_3_ labeling concept towards guanosine and ‐uridine and hence ensure utmost flexibility for labeling any nucleotide within an RNA target sequence. Additionally, we show first biochemical applications. These advancements hold great potential to accelerate the adoption and utilization of the 2′‐OCF_3_ RNA approach on a much broader scale.

## Experimental Section

For the syntheses and characterization data of compounds **G2** to **G9** and **U1** to **U3** see the Supporting Information.

### RNA solid‐phase synthesis

Standard phosphoramidite chemistry was applied for RNA strand elongation and incorporation of 2′‐OCF_3_ modified nucleoside phosphoramidites: 2′‐*O*‐TOM standard RNA nucleoside phosphoramidite building blocks and 2’‐*O*‐TBDMS 1000 Å CPG solid support were purchased from ChemGenes. All oligonucleotides were synthesized on ABI 391/392 or a K&A H‐6/H‐8 nucleic acid synthesizers following standard methods: detritylation (90 sec) with dichloroacetic acid/1,2‐dichloroethane (4/96); coupling (5.0 min) with phosphoramidites/acetonitrile (100 mM, 200 μL) and benzylthiotetrazole/acetonitrile (300 mM, 500 μL); capping (2×25 sec) with Cap A/Cap B (1/1), Cap A: 4‐(dimethylamino)pyridine/acetonitrile (500 mM), Cap B: acetic anhydride/*sym*‐collidine/acetonitrile (2/3/5); oxidation (60 sec) with iodine (20 mM) in tetrahydrofuran/pyridine/H_2_O (35/10/5). Solutions of phosphoramidites and tetrazole were dried over activated molecular sieves (3 Å) overnight.

### Deprotection of 2′‐OCF_3_ modified RNA

Solid support was treated with methylamine/ethanol (33 %, 0.7 mL) and methylamine/H_2_O (40 %, 0.7 mL) for 6 h at 37 °C. Supernatant was removed and solid support was washed thrice with tetrahydrofuran/H_2_O (1/1). Combined supernatant and washings were evaporated to dryness and the residue was dissolved in a solution of tetrabutylammonium fluoride in tetrahydrofuran (1.0 M, 1.5 mL) and incubated for 16 h at 37 °C for removal of 2’‐O‐silyl protecting groups. The reaction was quenched by addition of tetraethylammonium acetate/H_2_O (1.0 M, 1.5 mL, pH 7.4). The solution was reduced to one third of the original volume and desalted with size‐exclusion column chromatography (GE Healthcare, HiPrep^TM^ 26/10 Desalting; Sephadex G25) eluting with H_2_O; collected fractions were evaporated and the RNA dissolved in H_2_O (1 mL) for immediate use or storage at −20 °C.

### Purification of 2′‐OCF_3_ modified RNA

Crude RNA was purified by anion exchange chromatography on a semipreparative Dionex DNAPac® PA‐100 column (9 mm×250 mm) at 80 °C with 2 mL/min flow rate (Eluent A: 20 mM NaClO_4_ and 25 mM Tris‐HCl (pH 8.0) in 20 % aqueous acetonitrile ; Eluent B: 0.6 M NaClO_4_ and 25 mM Tris‐HCl (pH 8.0) in 20 % aqueous acetonitrile. Fractions containing RNA were diluted with 0.1 M triethylammonium bicarbonate solution, loaded on a C18 SepPak Plus® cartridge (Waters/Millipore), washed with H_2_O and eluted with acetonitrile/H_2_O (1/1).

### HPLC analysis and quantification of 2′‐OCF_3_ modified RNA

Analysis of crude and purified RNA was performed by anion exchange chromatography on a Dionex DNAPac® PA‐100 column (4 mm×250 mm) at 80 °C with flow rate of 1 mL/min. For RNA shorter or equal to 15 nucleotides, a gradient of 0–40 % B in 30 min and for RNA longer than 15 nucleotides a gradient of 0–60 % B was used; Eluent A: 20 mM NaClO_4_ and 25 mM Tris‐HCl (pH 8.0) in 20 % aqueous acetonitrile; Eluent B: 0.6 M NaClO_4_ and 25 mM Tris‐HCl (pH 8.0) in 20 % aqueous acetonitrile. HPLC traces were recorded at UV absorption at 260 nm. The RNA was quantified on an Implen P300 Nanophotometer.

### Mass spectrometry of 2′‐OCF_3_ modified RNA

RNA samples (3 μL) were diluted with 40 mM Na_2_H_2_(EDTA)/H_2_O (5/4) for a total volume of 30 μL, injected onto C18 XBridge 2.5 μm (2.1 mm×50 mm) at a flow rate of 0.1 mL/min and eluted with 0–100 % B gradient at 30 °C (Eluent A: 8.6 mM triethylamine, 100 mM 1,1,1,3,3,3‐hexafluoroisopropanol in H_2_O; Eluent B: methanol). RNA traces were analyzed on a Finnigan LCQ Advantage Max electrospray ionization mass spectrometer with 4.0 kV spray voltage in negative mode.

### NMR measurements of 2′‐OCF_3_ modified RNA

RNA samples were lyophilized as triethylammonium salts and dissolved either in 280 μL or 400 μL NMR buffer (15 mM Na[AsO_2_(CH_3_)_2_] ⋅ 3H_2_O, 25 mM NaCl, 3 mM NaN_3_, in D_2_O or 9/1 H_2_O/D_2_O, pH 6.5) and transferred into restricted volume Shigemi tubes or standard 5 mm NMR tubes. Sample concentrations varied between 0.1 and 1 mM and experiments were run at 298 K unless otherwise stated. All NMR experiments were conducted on a Bruker 600 MHz Avance II+ NMR or a 700 MHz Avance Neo NMR both equipped with a Prodigy TCI probe.

1D ^19^F NMR spectra were typically acquired using the following parameters: spectral width 10 ppm, o1p −60 ppm, 32k complex data points. 128 scans were collected with a recycling delay of 1 s resulting in an experimental time of 4 min.

For the 2D ^19^F‐^13^C HMQC experiments at natural ^13^C abundance the following parameters were used: spectral width in the indirect ^13^C dimension was set to 10 ppm, and the spectral width in the direct ^19^F dimension was set to 10 ppm. A total of 64 complex points was collected in the indirect ^13^C dimension (acquisition time=21 ms) and 1024 complex points were collected in the direct ^19^F dimension (acquisition time=91 ms). 768 scans were collected with a recycling delay of 1 s resulting in an experimental time of 16 h. The carrier frequency was placed at −58 ppm in the ^19^F dimension and in the ^13^C dimension at 120 ppm. The ^1^J_CF_ coupling constant was set to 270 Hz.

The pulse sequence for the ^19^F CPMG relaxation dispersion experiment was previously published.^[78]^ Per CPMG field strength 256 scans were collected and a CPMG relaxation delay of 16 ms was used. Data was acquired at twenty‐four CPMG field strengths (0, 125, 250 (2×), 500, 750, 1000 (2×), 1250 (2×), 1500, 1875, 2250, 2500, 2750 (2×), 3125, 3375, 3750 (2×), 4000, 4250, 4500, and 5000 Hz. The ^19^F CPMG relaxation dispersion experiments were run at 565 and 659 KHz and each spectrum took ca. 3 h measurement time. The spectra were processed using Topspin 4.2.0 and the dispersion profile was fitted to the Carver‐Richard equation by using an in‐house written MATLAB script.

For experimental details concerning RNA interference and analysis of gene silencing see the Supporting Information.

## Supporting Information

The authors have cited additional references within the Supporting Information.[^79^, ^80^]

## Conflict of interest

The authors declare no conflict of interest.

1

## Supporting information

As a service to our authors and readers, this journal provides supporting information supplied by the authors. Such materials are peer reviewed and may be re‐organized for online delivery, but are not copy‐edited or typeset. Technical support issues arising from supporting information (other than missing files) should be addressed to the authors.

Supporting Information

## Data Availability

The data that support the findings of this study are available from the corresponding author upon reasonable request.
