# Supplementary material for: Advances in RNA Labeling with Trifluoromethyl Groups
Source: Chemistry. 2023 Sep 20;29(60):e202302220. doi: 10.1002/chem.202302220 (PMC10947337; doi:10.1002/chem.202302220)
Supplement: Supplementary file 1 — Supporting Information [file CHEM-29-0-s001.pdf]

Chemistry–A European Journal

Supporting Information

Advances in RNA Labeling with Trifluoromethyl Groups

Clemens Eichler, Maximilian Himmelstoß, Raphael Plangger, Leonie I. Weber, Markus Hartl, Christoph Kreutz,* and Ronald Micura*

Contents

Supporting Methods

General 2
NMR measurements of compounds 2
High-resolution mass spectrometry of compounds 2
RNA interference and analysis of gene silencing 2

Synthesis of 2'-OCF_3 guanosine phosphoramidite **G9** 4

Synthesis of 2'-OCF_3 uridine phosphoramidite **U3** 23

Supporting Tables

Table S1 32
Table S2 33

References 34

Supporting Methods

General

^{1}H, ^{13}C, ^{31}P and ^{19}F NMR measurements were performed on Bruker Avance 4 Neo 400 MHz or 700 MHz NMR spectrometers. Chemical shifts are referenced to deuterated solvents (DMSO-d_6 (2.50 ppm – ^{1}H; 39.5 ppm – ^{13}C; $CDCl_3$ (7.26 ppm – ^{1}H; 77.1 – ^{13}C); ^{31}P shifts are relative to external 85% phosphoric acid, ^{19}F shifts are relative to external CCl_3F). Assignment of ^{1}H and ^{13}C signals was carried out using COSY, HSQC and HMBC experiments. Mass spectra were recorded on a Finnigan LCQ Advantage MAX ion trap instrument connected to a Thermo Fisher Ultimate 3000 HPLC system. RNAs were analyzed in negative-ion mode with a potential of -4kV applied to the spray needle. Sample preparation: 200 pmol RNA dissolved in 30 µL of aqueous 20 mM ethylendiamine tetraacetic acid solution; injection volume: 30 µL; column: Waters XTerraMS, C18, 2.5 µm, 1.0 × 50 mm, 21 °C; flow rate: 0.1 mL/min; eluent A: 8.6 mM triethylamine, 100 mM 1,1,1,3,3,3-hexafluoroisopropanol in H_2O (pH 8.0); eluent B: methanol; gradient 0-100% B in A within 30 min, UV detection at 254 nm. Thin layer chromatography was performed using POLYGRAM® SIL G/UV_{254} pre-coated polyester sheets (0.2 mm silica gel with fluorescent indicator). Preparative column chromatography was carried out using silica gel (60 Å, 70-230 mesh, 63-200 µm). Chemical reagents and solvents were purchased from commercial suppliers and used without further purification.

NMR measurements of compounds

^{1}H, ^{13}C, ^{19}F and ^{31}P spectra were recorded on a Bruker Ultrashield™ 400 Plus spectrometer. Chemical shifts (δ) are reported relative to tetramethylsilane (TMS), referenced to the residual solvent signal (DMSO-d_6: 2.50 ppm for ^{1}H and 39.52 ppm for ^{13}C spectra; $CDCl_3$: 7.26 ppm for ^{1}H and 77.16 ppm for ^{13}C spectra). The following abbreviations were used to denote multiplicities: s = singulet, d = doublet, t = triplet, q = quadruplet, m = multiplet, b = broad. Signal assignments are based on ^{1}H-^{1}H-COSY, ^{1}H-^{13}C-HSQC, ^{1}H-^{13}C-HMBC experiments.

High-resolution mass spectrometry of compounds

High resolution mass spectra were recorded in positive ion mode on a Thermo Scientific Q Exactive Orbitrap, ionized via electrospray at 3.7 kV spray voltage.

RNA interference and analysis of gene silencing

Lyophilized synthetic siRNA duplexes were dissolved, annealed, and delivered into DF-1 cells by electroporation as described previously.[73] Total RNA isolation, gel electrophoresis, and Northern blotting was done as described.[73] To prepare digoxigenin (DIG)-dUTP labeled probes specific for chicken *BASP1* or *GAPDH*, 300-bp segments from the respective coding regions were amplified by polymerase chain reaction (PCR) using the PCR DIG Probe Synthesis Kit (Roche, Cat. No. 11636090910), and *BASP1* (5'-GTTGAGAAGGATGCTCAGGTC-3'/5'-TTTGCTCTTGTCATCTGCTTTG-3') or *GAPDH* (5'-GCAGGTGCTGAGTATGTTG-3'/5'-ATCCACCGTCTTCTGTG-TG-3') specific primer pairs. PCR was performed with 30 cycles (95°C,

30 s; 50°C, 30 s, 72°C, 40 s) using 2 ng of DNA template obtained from a preceding PCR performed under standard conditions. The total yield of the DIG-labeled probes was 4 µg each. Filters were hybridized at 40 °C as described in ref. [73] in a buffer containing 50% (v/v) formamide, 5x SSC, 0.1% (w/v) lauroylsarcosine, 0.02% (w/v) SDS, and 2% (v/v) Blocking Solution (Roche, Cat. No. 11585762001). After prehybridization for 24 h, filters were hybridized for another 24 h in presence of the DIG-labeled probes (50 ng/ml). Filters were finally washed in 0.2x SSC, 0.1% (w/v) SDS at 60 °C. Afterwards, filters were rinsed in Washing Solution (Roche, Cat. No. 11585762001), and then incubated for 2 h in 1% (w/v) Blocking Reagent (Roche, Cat. No. 11363514910). Filters were subsequently incubated for 60 min in 1% (w/v) Blocking Reagent containing 1:10,000 diluted anti-Digoxigenin-AP (75 mU/µl) (Roche, Cat. No.11363514910), and then washed three times in Washing Solution. Alkaline phosphatase (AP) activity was detected in a buffer containing 0.1 M NaCl, 0.1 M Tris-HCl pH 9.5 and the 1:100 diluted chemoluminescent substrate disodium 3-(4-methoxy-spiro{1,2-dioxetane-3,2'-(5'-chloro) tricyclo [3.3.1.13,7]decan}-4-yl)phenyl phosphate (CSPD) (Roche, Cat. No. 11363514910). Signals were detected on a Fusion FX imaging system (Vilber) with an exposure time of 10 min, and quantified using the program ImageQuant TL (GE Healthcare). Quantitative PCR (qPCR) was performed as described in ref. [63] using specific chicken *BASP1* (5'- ACTTTCTGCCAACTTGACAC-3'/5'-TCACTCCCAAACCCCATTAC-3') and *GAPDH* (5'-TCTTCACCACCGCTCAGT-TC-3'/5'-TCAGTTTCTATCAGCCTCTCCC-3') primer pairs. qPCR was performed on a Step One Real-Time PCR System (Applied Biosystems) with 45 cycles (95°C, 15 s; 60°C, 30 s) using 2.5 ng of cDNA template and the Luna® Universal qPCR Master kit (New England BioLabs). Melting curve analyses of the PCR products were performed at the end of each run. The cDNA templates were generated by reverse transcription using the LunaScript® RT SuperMix kit (New England BioLabs). Thereby, each 1 µg of total RNA was mixed with random hexamer and oligo-dT primers, dNTPs, murine RNase inhibitor, and Luna® reverse transcriptase in a final volume of 20 µl. The reaction was incubated in a thermocycler for 2 min at 25 °C, 10 min at 55 °C, 1 min at 95 °C, and then cooled down to 4 °C. Quantification of the signals was performed using the OneStep software (Applied Biosystems).

Immunoblot analysis was done as described.[74] To generate the extracts, cells grown on 60-mm dishes were washed with phosphate buffered saline (PBS) followed by addition of 200 µl of RIPA lysis buffer supplemented with protease inhibitors. Scraped cells were incubated on ice for 20 min and then centrifuged at 6,000 × g for 10 min at 4 °C. Clarified lysates were quantified by the Bradford assay and protein gelelectrophoresis carried out using each 20 µg of total protein. The polyclonal chicken BASP1-specific antibody (anti-BASP1) directed against a C-terminal BASP1 peptide has been described.[69] The monoclonal anti-GAPDH (#AB8245, Abcam, Cambridge, UK) was applied in a 1:10,000 dilution.

Synthesis of 2'-OCF$_3$ guanosine phosphoramidite G9

2',3',5',*N*[2]-Tetra-*O*-acetylguanosine (G2)

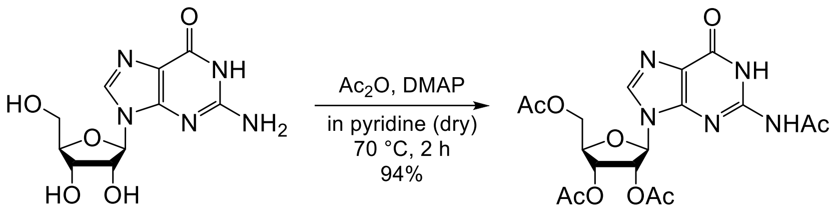

G1 **G2**

To a suspension of guanosine (5.00 g, 17.7 mmol) and dimethylaminopyridine (DMAP, 1.08 g, 8.83 mmol) in dry pyridine (40 mL), acetic anhydride (10.8 g, 10.0 mL, 106 mmol) was added. The reaction mixture was stirred at 70 °C for 2 hours, allowed to cool to ambient temperature, quenched with methanol (2 mL) and concentrated under reduced pressure. The residue was dissolved in dichloromethane (200 mL), washed with 1 M hydrochloric acid and saturated sodium bicarbonate solution (each 50 mL), dried over sodium sulfate and the solvent evaporated. The Crude product was purified by column chromatography on silica gel using 1 - 5% methanol in dichloromethane as eluent. Yield: 7.50 g of compound **G2** as white foam (94%). TLC (dichloromethane/methanol, 9/1): R_F = 0.43. ESI-MS: (m/z) $[M+H]^+$ calcd. 452.1412; found: 452.1408. ^{1}H-NMR (400 MHz, DMSO-*d$_6$*, 25 °C, δ [ppm]): δ = 2.04 (6H, d, J=4.92 Hz, ***H$_3$***C-(2',3'-*O*-Ac)), 2.12 (3H, s, ***H$_3$***C-(5'-*O*-Ac)), 2.19 (3H, s, ***H$_3$***C-(*N*-Ac)), 4.34 (3H, m, J=5.15 Hz, ***H***-C(4'), -***H$_2$***C-(5')), 5.48 (1H, dd, J=3.53, 5.84 Hz, ***H***-C(3')), 5.82 (1H, t, J=6.14 Hz, ***H***-C(2')), 6.09 (1H, d, J=6.36 Hz, ***H***-C(1')), 8.25 (1H, s, ***H***-C(8)), 11.66 (1H, s, -***H***N-C(2)), 12.09 (1H, s, ***H***-N(1)). ^{13}C-NMR (100 MHz, DMSO-*d$_6$*, 25 °C, δ [ppm]): δ = 20.35 (3C, t, J=13.51 Hz, -***C***H$_3$(*O*-Ac)), 23.87 (1C, s, -***C***H$_3$(*N*-Ac)), 63.11 (1C, s, ***C***(5')), 70.35 (1C, s, ***C***(3')), 72.22 (1C, s, ***C***(2')), 79.88 (1C, s, ***C***(4')), 84.59 (1C, s, ***C***(1')), 120.43 (1C, s, ***C***(5)), 137.87 (1C, s, ***C***(8)), 148.29 (1C, s, ***C***(2)), 148.64 (1C, s, ***C***(4)), 154.75 (1C, s, ***C***(6)), 169.37 (2C, d, J=14.63 Hz, -***C***O-(2',3'-*O*-Ac)), 170.12 (1C, s, -***C***O-(5'-*O*-Ac)), 173.58 (1C, s, -***C***O-(*N*-Ac)).

^{1}H-NMR (400 MHz, DMSO-d_6, 25 °C) of compound **G2**

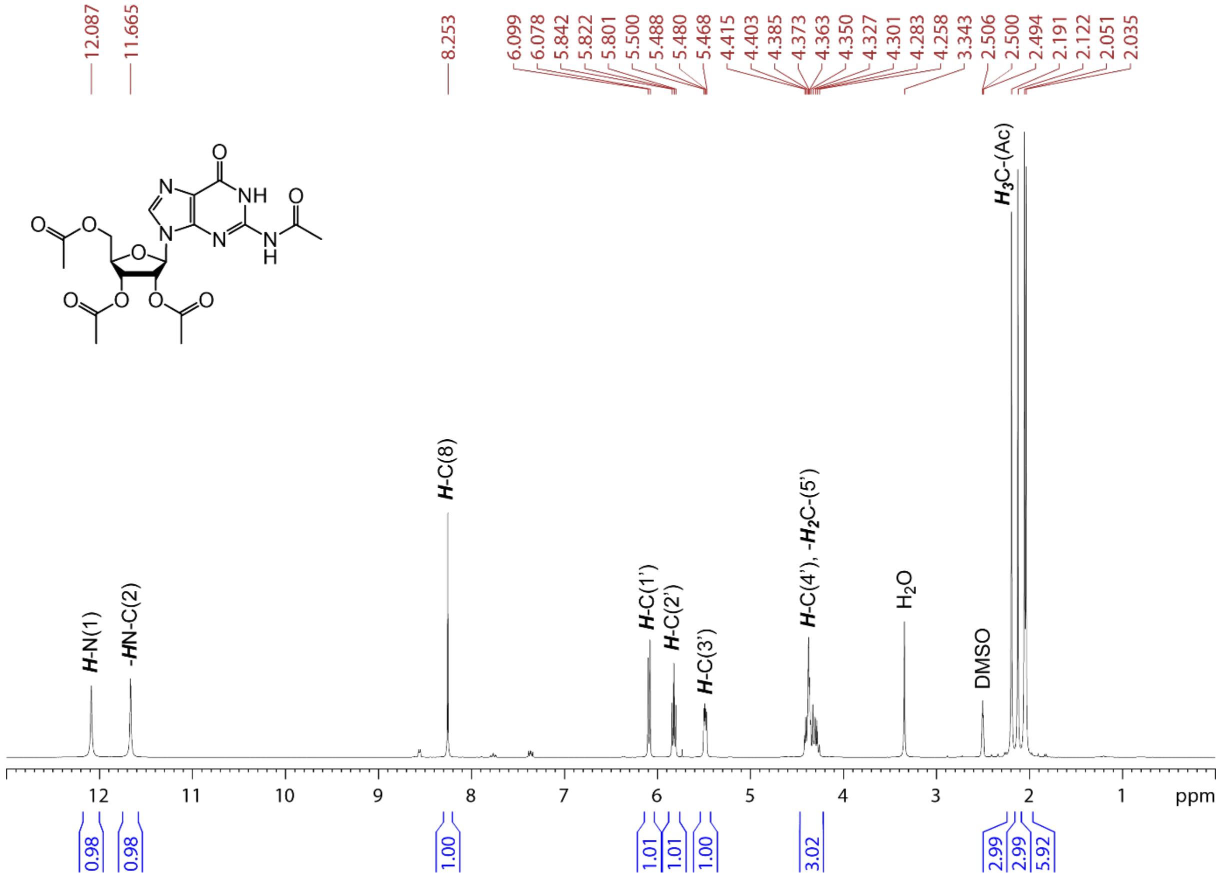

^{13}C-NMR (100 MHz, DMSO-d_6, 25 °C) of compound **G2**

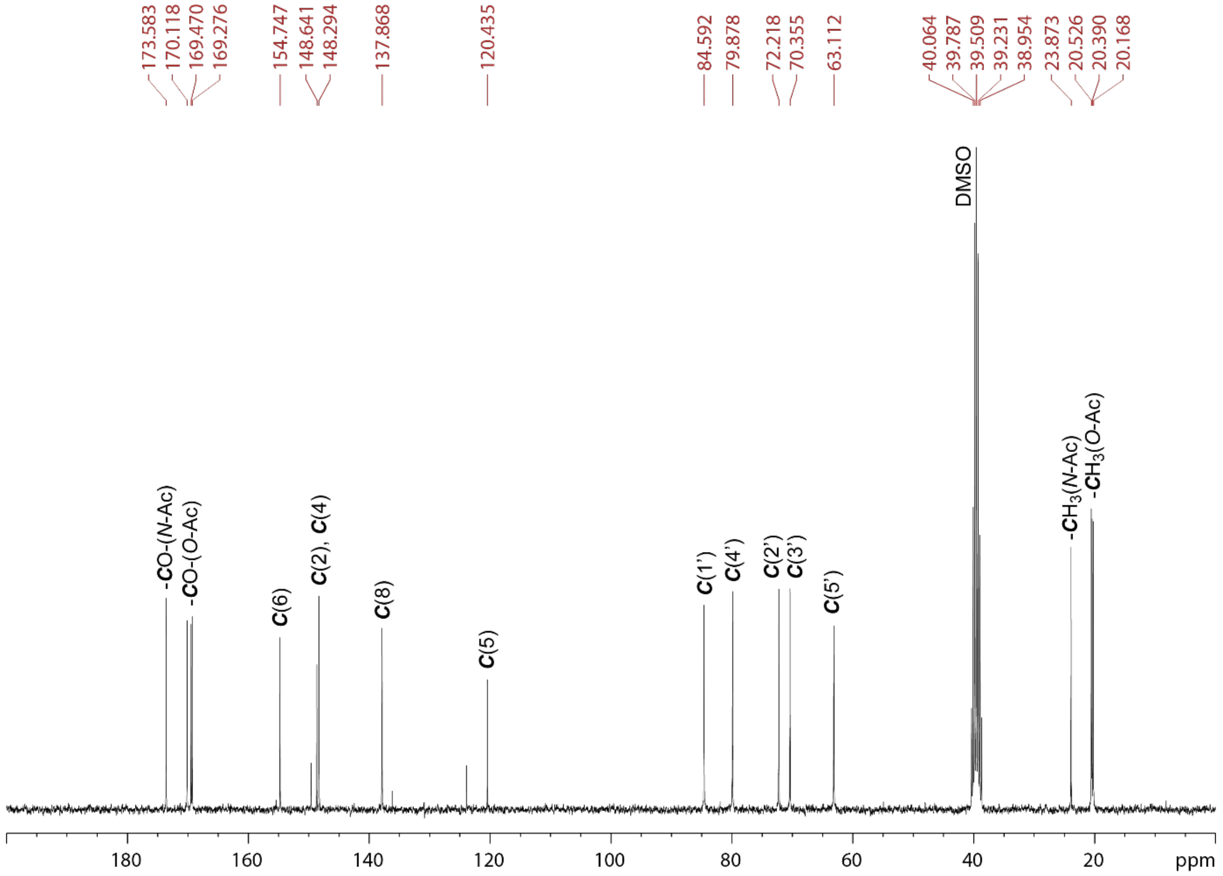

2',3',5',*N*²-Tetra-*O*-acetyl-*O*⁶-[2-(4-nitrophenyl)ethyl]guanosine (G3)

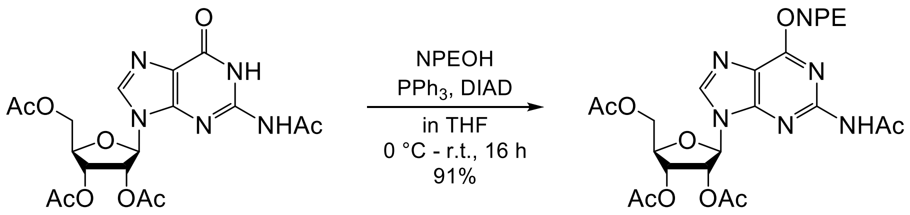

G2 **G3**

To a solution of compound **G2** (7.50 g, 16.6 mmol), triphenylphosphine (6.54 g, 24.9 mmol) and 4-nitrophenylethanol (NPEOH, 4.17 g, 24.9 mmol) in dry tetrahydrofuran (130 mL) at 0 °C was added dropwise diisopropyl azodicarboxylate (DIAD, 4.91 mL, 5.04 g, 24.9 mmol). The solution was allowed to warm up to room temperature and stirred for 16 hours. Upon complete conversion of starting material, the reaction was quenched by addition of methanol (3 mL) and concentrated. The residual oil was dissolved in ethyl acetate and washed with saturated sodium bicarbonate solution, 5% hydrogen peroxide solution and brine. Organic phases were combined, treated with zinc chloride (6.75 g, 50.0 mmol), stirred until complete dissolvement, left to stand for 2 h at room temperature and filtered. All volatiles were evaporated and the crude product was purified by column chromatography on silica gel using 0 - 4% methanol in dichloromethane as eluent. Yield: 7.49 g of compound **G3** as a white foam (91%). TLC (dichloromethane/methanol, 9/1): R_F = 0.55. ESI-MS: (m/z) $[M+H]^+$ calcd. 601.1889; found: 601.1879. ^{1}H-NMR (400 MHz, $CDCl_3$, 25 °C, δ [ppm]): δ = 2.03 (6H, d, J=5.56 Hz, ***H*3**C-(2',3'-*O*-Ac)), 2.08 (3H, s, ***H*3**C-(5'-*O*-Ac)), 2.43 (3H, s, ***H*3**C-(*N*-Ac)), 3.24 (2H, t, J=6.68 Hz, -***H*2**C-(NPE)), 4.39 (3H, m, J=5.68 Hz, ***H***-C(4'), -***H*2**C-(5')), 4.72 (2H, t, J=6.70 Hz, -***H*2**C-(NPE)), 5.67 (1H, t, J=5.20 Hz, ***H***-C(3')), 5.88 (1H, t, J=5.00 Hz, ***H***-C(2')), 6.02 (1H, d, J=4.44 Hz, ***H***-C(1')), 7.44 (2H, d, J=8.60 Hz, ***H***-C(NPE)), 7.92 (1H, s, ***H***-C(8)), 8.08 (2H, d, J=8.52 Hz, ***H***-C(NPE)), 8.34 (1H, s, -***H***N-C(2)). ^{13}C-NMR (100 MHz, $CDCl_3$, 25 °C, δ [ppm]): δ = 20.51 (3C, t, J=17.11 Hz, -***C***H3(*O*-Ac)), 25.12 (1C, s, -***C***H3(*N*-Ac)), 34.92 (1C, s, -***C***H2-(NPE)), 63.02 (1C, s, ***C***(5')), 66.94 (1C, s, -***C***H2-(NPE)), 70.37 (1C, s, ***C***(3')), 73.07 (1C, s, ***C***(2')), 79.90 (1C, s, ***C***(4')), 86.98 (1C, s, ***C***(1')), 118.39 (1C, s, ***C***(5)), 123.69 (2C, s, ***C***H-(NPE)), 129.92 (2C, s, ***C***H-(NPE)), 140.24 (1C, s, ***C***(8)), 145.60 (1C, s, ***C***(NPE)), 146.80 (1C, s, ***C***(NPE)), 152.27 (2C, d, J=13.79 Hz, ***C***(2), ***C***(4)), 160.63 (1C, s, ***C***(6)), 169.46 (2C, d, J=18.67 Hz, -***C***O-(2',3'-*O*-Ac)), 170.43 (1C, s, -***C***O-(5'-*O*-Ac)).

^{1}H-NMR (400 MHz, $CDCl_3$, 25 °C) of compound **G3**

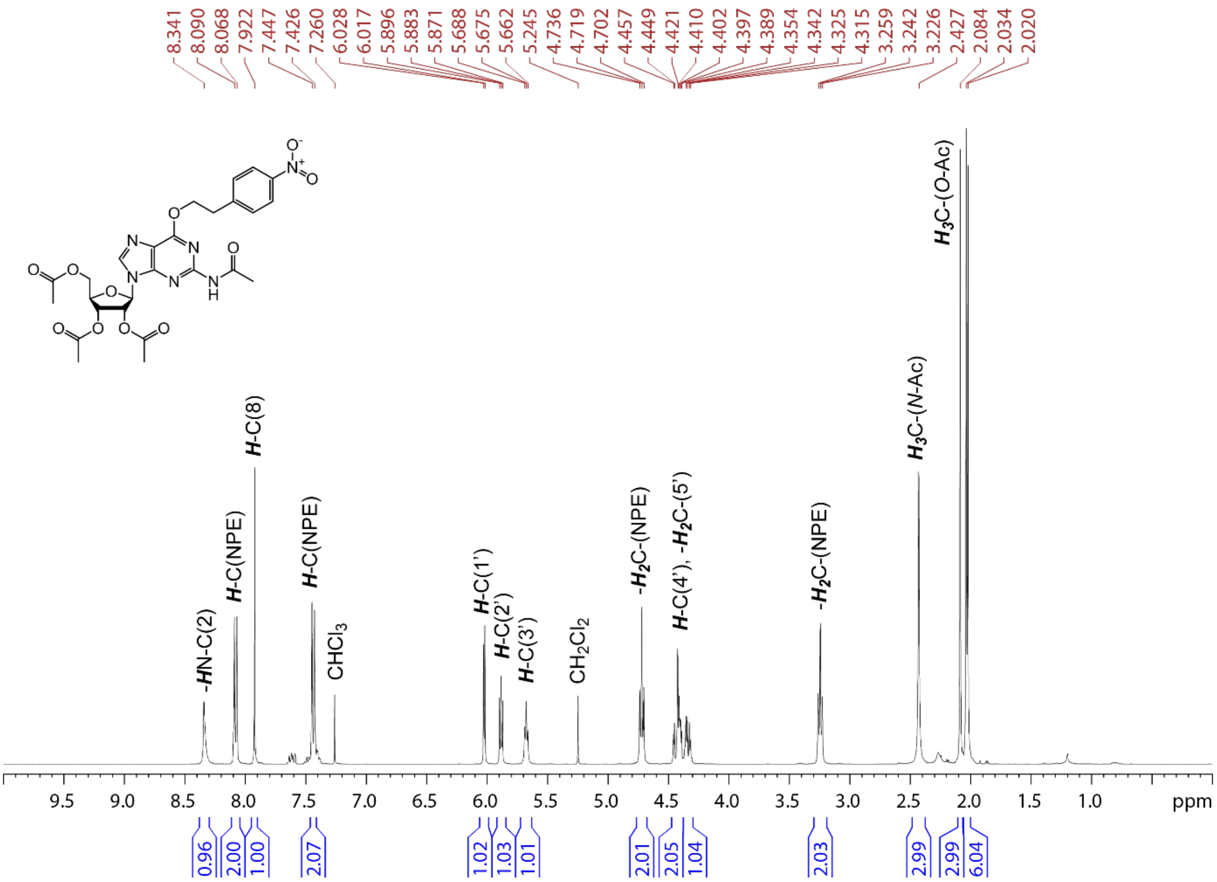

^{13}C-NMR (100 MHz, $CDCl_3$, 25 °C) of compound **G3**

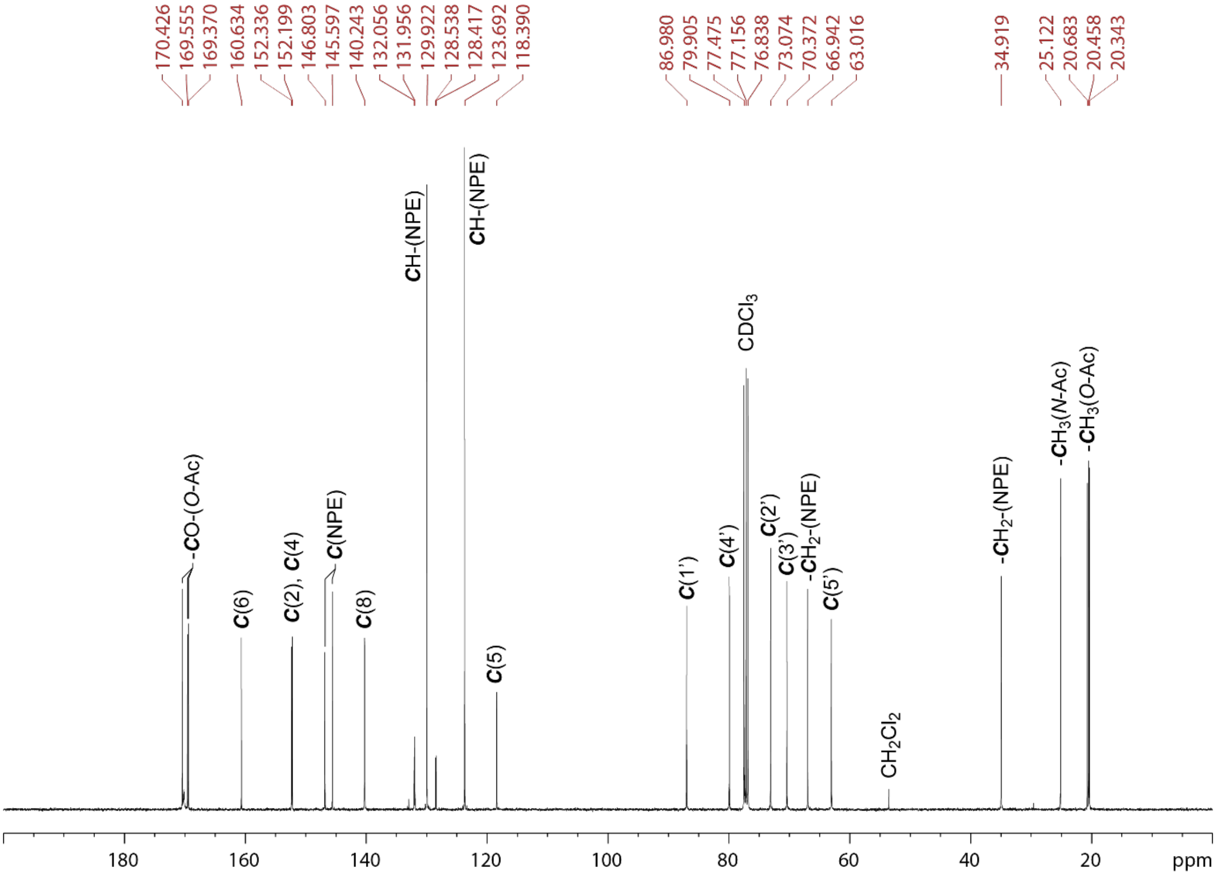

N^{2}**-Acetyl-*O***^{6}**-[2-(4-nitrophenyl)ethyl]guanosine (G4)**

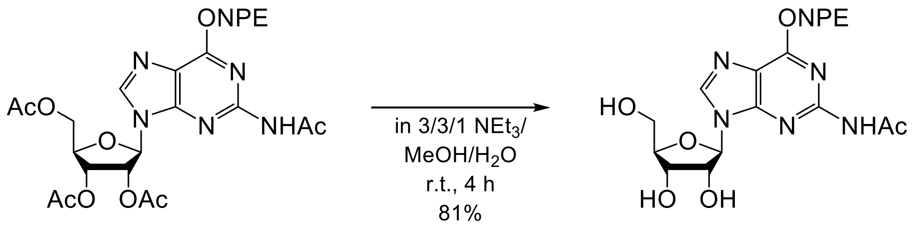

G3 **G4**

A solution of compound **G3** (7.51 g, 12.5 mmol) in triethylamine (150 mL), methanol (150 mL) and water (50 mL) was stirred for 4 hours at ambient temperatures. Upon completion, all volatiles were evaporated and the residue was charged on silica gel. Crude product was purified by column chromatography on silica gel using 0 - 10% methanol in dichloromethane as eluent. Yield: 4.81 g of compound **G4** as a white solid (81%). TLC (dichloromethane/ methanol, 85/15): R_F = 0.54. ESI-MS: (m/z) $[M+H]^+$ calcd. 475.1572; found: 475.1568. ^{1}H-NMR (400 MHz, DMSO-d_6, 25 °C, δ [ppm]): δ = 2.23 (3H, s, ***H*****3**C-(Ac)), 3.33 (2H, t, J=6.80 Hz, -***H*****2**C-(NPE)), 3.60 (2H, md, J=4.35, 42.07 Hz, -***H*****2**C-(5')), 3.93 (1H, q, J=3.97 Hz, ***H***-C(4')), 4.18 (1H, q, J=4.40 Hz, ***H***-C(3')), 4.58 (1H, q, J=5.60 Hz, ***H***-C(2')), 4.79 (2H, t, J=6.82 Hz, -***H*****2**C-(NPE)), 4.97 (1H, t, J=5.52 Hz, ***H***O-C(5')), 5.18 (1H, d, J=4.76 Hz, ***H***O-C(3')), 5.47 (1H, d, J=5.92 Hz, ***H***O-C(2')), 5.89 (1H, d, J=5.84 Hz, ***H***-C(1')), 7.66 (2H, d, J=8.76 Hz, ***H***-C(NPE)), 8.19 (2H, d, J=8.76 Hz, ***H***-C(NPE)), 8.45 (1H, s, ***H***-C(8)), 10.45 (1H, s, -***H***N-C(2)). ^{13}C-NMR (100 MHz, DMSO-d_6, 25 °C, δ [ppm]): δ = 24.70 (1C, s, -***C***H3(Ac)), 34.18 (1C, s, -***C***H2-(NPE)), 61.33 (1C, s, ***C***(5')), 66.44 (1C, s, -***C***H2-(NPE)), 70.36 (1C, s, ***C***(3')), 73.60 (1C, s, ***C***(2')), 85.59 (1C, s, ***C***(4')), 87.13 (1C, s, ***C***(1')), 117.24 (1C, s, ***C***(5)), 123.44 (2C, s, ***C***H-(NPE)), 130.35 (2C, s, ***C***H-(NPE)), 141.31 (1C, s, ***C***(8)), 146.39 (2C, d, J=18.21 Hz, ***C***(NPE)), 152.06 (1C, s, ***C***(2)), 153.01 (1C, s, ***C***(4)), 159.74 (1C, s, ***C***(6)), 168.98 (1C, s, -***C***O-(Ac)).

^{1}H-NMR (400 MHz, DMSO-d_6, 25 °C) of compound **G4**

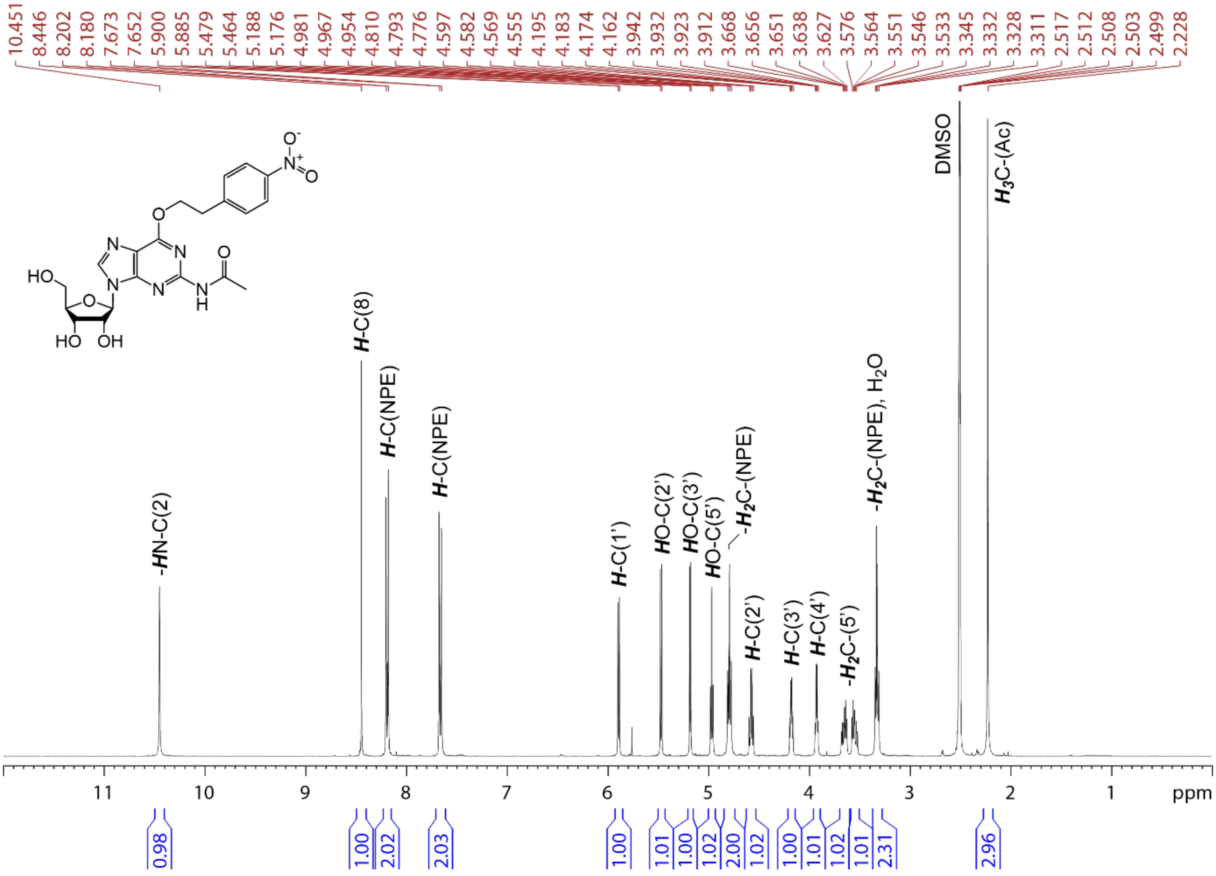

^{13}C-NMR (100 MHz, DMSO-d_6, 25 °C) of compound **G4**

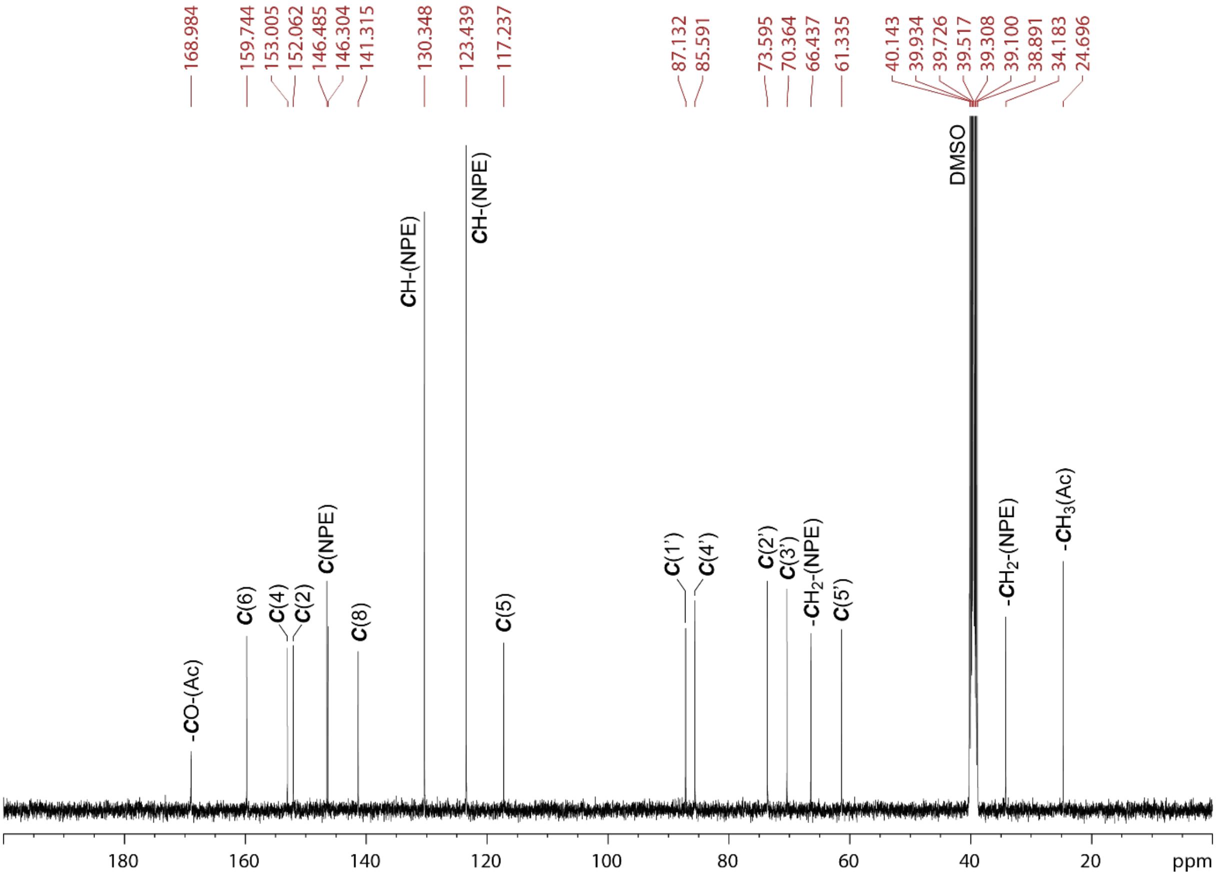

***N*²-Acetyl-*O*⁶-[2-(4-nitrophenyl)ethyl]-3',5'-*O*-(1,1,3,3-tetraisopropyldisiloxane-1,3-diyl)guanosine (G5)**

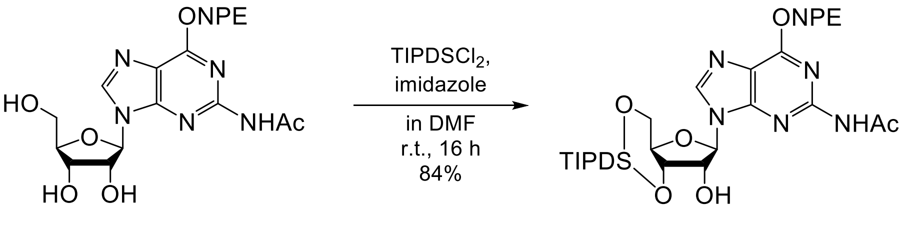

G4 **G5**

To a solution of compound **G4** (3.60 g, 7.60 mmol) and imidazole (1.29 g, 19.0 mmol) in dry dimethylformamide (22 mL) was added 1,3-dichloro-1,1,3,3-tetraisopropyl disiloxane ($TIPDSCl_2$, 2.55 mL, 2.52 g, 7.98 mmol) dropwise while stirring at ambient temperatures. After 16 hours, TLC showed complete conversion of starting material and the solution was triturated with water (110 mL). The precipitate was filtered off, washed with water and dissolved in ethyl acetate. Organic phases were washed twice with brine, dried over sodium sulfate and evaporated. Crude product was purified by column chromatography on silica gel and eluted with 25 - 75 % ethyl acetate in hexanes. Yield: 4.55 g of compound **G5** as white foam (84 %). TLC (hexanes/ethyl acetate, 1/3): R_F = 0.39. ESI-MS: (m/z) $[M+H]^+$ calcd. 717.3094; found: 717.3088. ^{1}H-NMR (400 MHz, $CDCl_3$, 25 °C, δ [ppm]): δ = 1.07 (28H, m, J=3.15 Hz, ***H*₃**C-, -***H***C-(TIPDS)), 2.55 (3H, s, ***H*₃**C-(Ac)), 3.07 (1H, d, J=1.72 Hz, ***H***O-C(2')), 3.31 (2H, t, J=6.76 Hz, -***H*₂**C-(NPE)), 4.10 (3H, m, J=4.53 Hz, ***H***-C(4'), -***H*₂**C-(5')), 4.44 (1H, td, J=1.70, 5.40 Hz, ***H***-C(2')), 4.68 (1H, dd, J=5.60, 7.44 Hz, ***H***-C(3')), 4.76 (2H, dq, J=3.81, 11.45 Hz, -***H*₂**C-(NPE)), 5.97 (1H, d, J=1.60 Hz, ***H***-C(1')), 7.50 (2H, d, J=8.68 Hz, ***H***-C(NPE)), 7.81 (1H, s, -***H***N-C(2)), 8.01 (1H, s, ***H***-C(8)), 8.17 (2H, dd, J=1.88, 6.84 Hz, ***H***-C(NPE)). ^{13}C-NMR (100 MHz, $CDCl_3$, 25 °C, δ [ppm]): δ = 13.13 (4C, q, J=25.47 Hz, -***C***H-(TIPDS)), 17.29 (8C, m, J=7.58 Hz, -***C***H_3(TIPDS)), 25.35 (1C, s, -***C***H_3(Ac)), 35.18 (1C, s, -***C***H_2-(NPE)), 61.39 (1C, s, ***C***(5')), 67.12 (1C, s, -***C***H_2-(NPE)), 70.39 (1C, s, ***C***(3')), 74.97 (1C, s, ***C***(2')), 82.26 (1C, s, ***C***(4')), 89.25 (1C, s, ***C***(1')), 118.40 (1C, s, ***C***(5)), 123.97 (2C, s, ***C***H-(NPE)), 130.09 (2C, s, ***C***H-(NPE)), 139.98 (1C, s, ***C***(8)), 145.65 (1C, s, ***C***(NPE)), 147.09 (1C, s, ***C***(NPE)), 152.26 (2C, d, J=3.58 Hz, ***C***(2), ***C***(4)), 160.72 (1C, s, ***C***(6)), 170.84 (1C, s, -***C***O-(Ac)).

^{1}H-NMR (400 MHz, $CDCl_3$, 25 °C) of compound **G5**

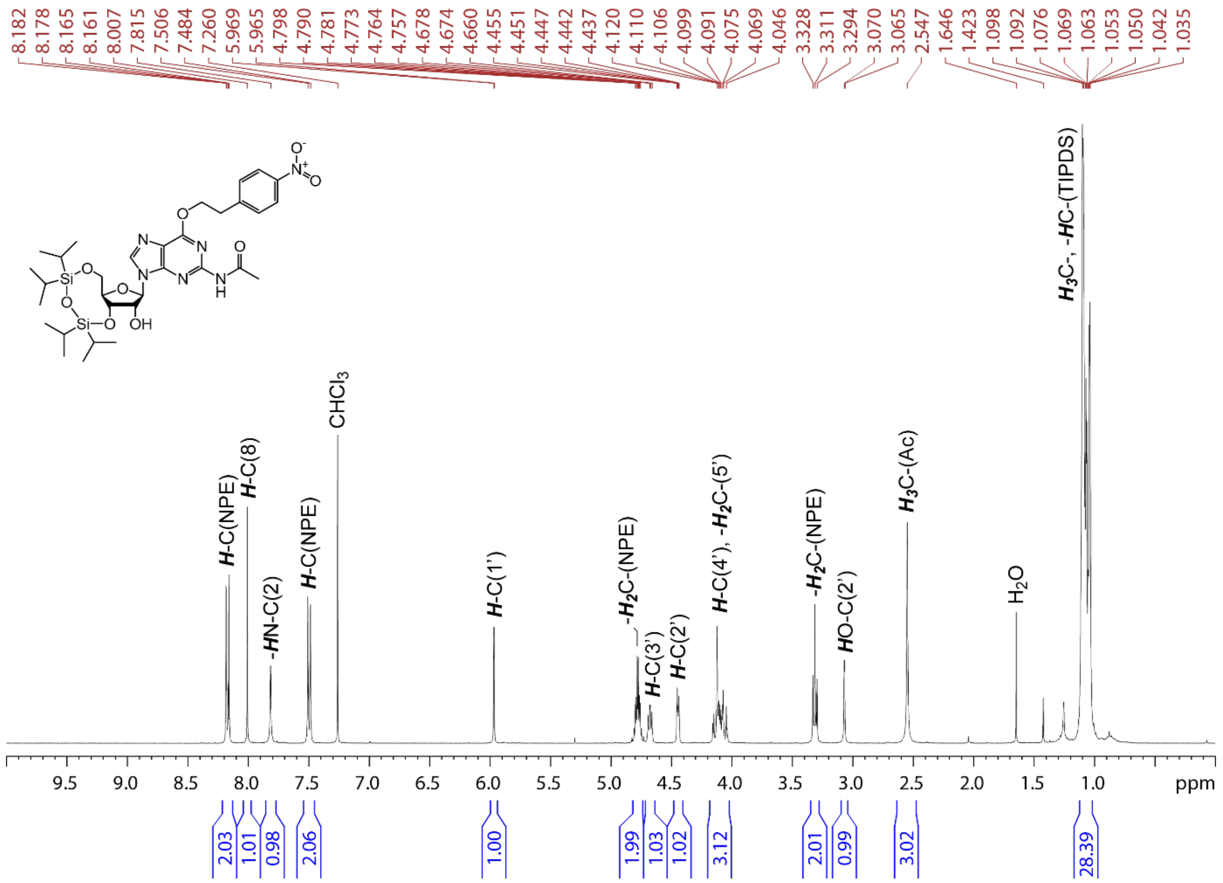

^{13}C-NMR (100 MHz, $CDCl_3$, 25 °C) of compound **G5**

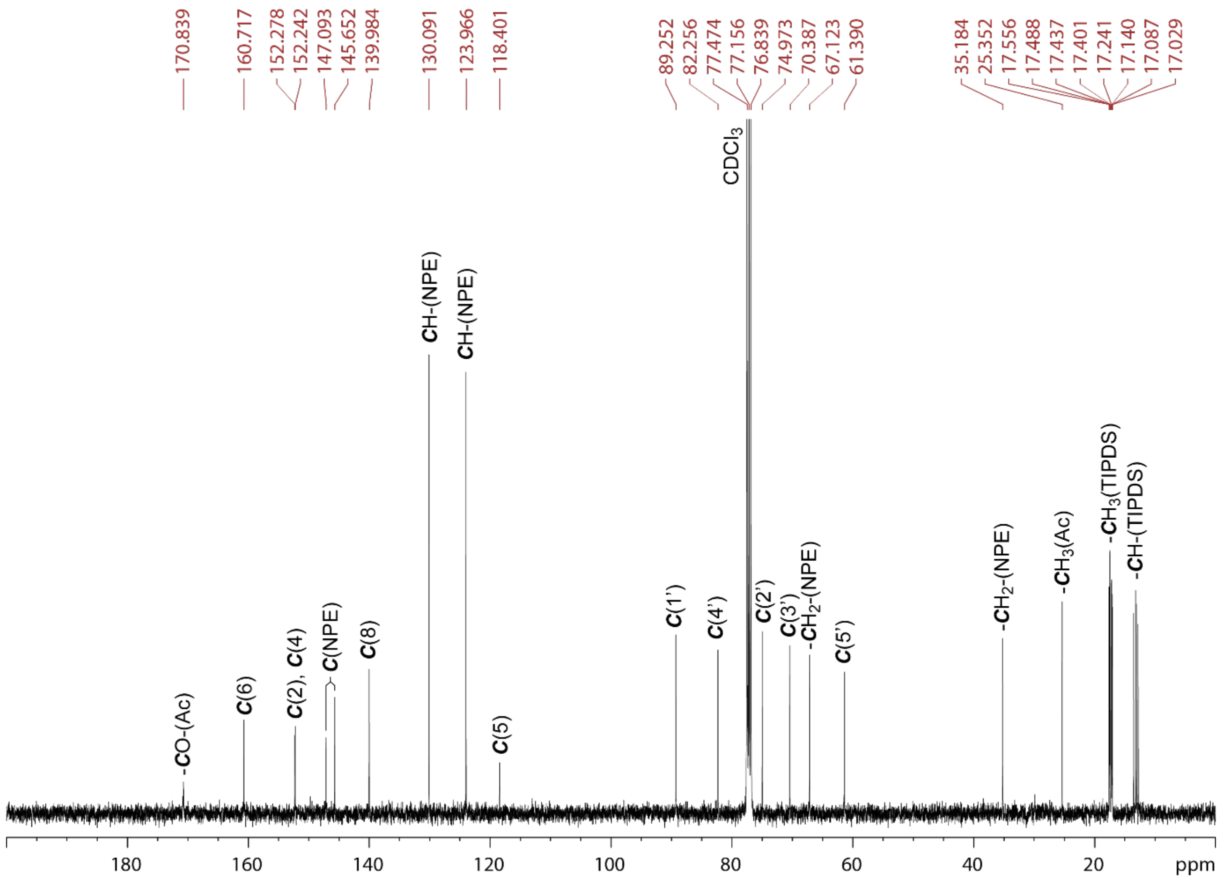

N^2-Acetyl-O^6-[2-(4-nitrophenyl)ethyl]-2'-*O*-[(methylthio)thiocarbonyl]-3',5'-*O*-(1,1,3,3-tetraisoproyldisiloxane-1,3-diyl)guanosine (G6)

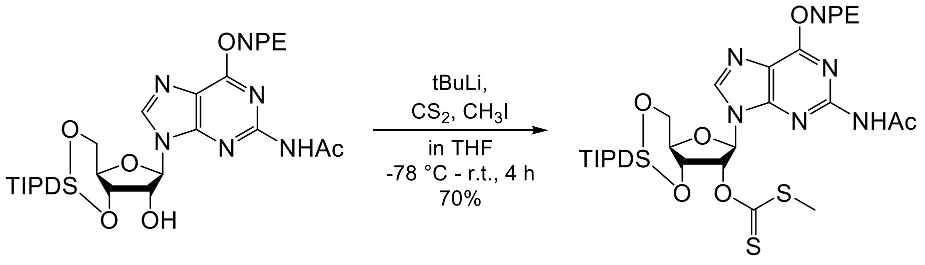

G5 **G6**

To a solution of compound **G5** (4.50 g, 6.28 mmol) in dry tetrahydrofuran (50 mL) was added *tert*-butyllithium solution (1.7 M in pentane, 4.62 mL, 7.85 mmol) at –78 °C. After 10 minutes carbon disulfide (3.39 mL, 4.30 g, 56.5 mmol) was added and stirred for 1 hour at –78 °C. To the reaction mixture was then added methyl iodide (488 µL, 1.11 g, 7.85 mmol) and the solution was allowed to warm to ambient temperatures for 3 hours. Upon completion, the reaction was quenched by addition of 5% citric acid solution (10 mL), concentrated and partitioned between ethyl acetate and saturated sodium bicarbonate solution. Organic phases were washed with saturated sodium bicarbonate solution and brine, dried over sodium sulfate and evaporated. Crude product was purified by column chromatography on silica gel using 20 - 50% ethyl acetate in hexanes as eluent. Yield: 3.55 g of compound **G6** as a white foam (70%). TLC (hexanes/ethyl acetate, 1/2): R_F = 0.63. ESI-MS: (m/z) $[M+H]^+$ calcd. 807.2692; found: 807.2685. ^{1}H-NMR (400 MHz, $CDCl_3$, 25 °C, δ [ppm]): δ = 1.05 (28H, m, J=7.43 Hz, ***H**₃*C-, -***H***C-(TIPDS)), 2.53 (3H, s, $\boldsymbol{H_3}$C-(Ac)), 2.61 (3H, s, $\boldsymbol{H_3}$C-S), 3.31 (2H, t, J=6.74 Hz, -$\boldsymbol{H_2}$C-(NPE)), 4.06 (1H, dd, J=2.36, 12.64 Hz, ***H(a)***-C(5')), 4.14 (1H, m, J=2.78 Hz, ***H***-C(4')), 4.19 (1H, dd, J=2.72, 12.76 Hz, ***H(b)***-C(5')), 4.78 (2H, t, J=6.72 Hz, -$\boldsymbol{H_2}$C-(NPE)), 4.87 (1H, dd, J=5.38, 8.34 Hz, ***H***-C(3')), 6.09 (1H, d, J=0.96 Hz, ***H***-C(1')), 6.48 (1H, d, J=5.16 Hz, ***H***-C(2')), 7.50 (2H, d, J=8.60 Hz, ***H***-C(NPE)), 7.77 (1H, d, J=3.72 Hz, -***H***N-C(2)), 8.02 (1H, s, ***H***-C(8)), 8.18 (2H, d, J=8.52 Hz, ***H***-C(NPE)). ^{13}C-NMR (100 MHz, $CDCl_3$, 25 °C, δ [ppm]): δ = 13.13 (4C, q, J=21.26 Hz, -***C***H-(TIPDS)), 17.20 (8C, m, J=10.03 Hz, -***C***H_3(TIPDS)), 19.38 (1C, s, ***C***H_3-S), 25.31 (1C, s, -***C***H_3(Ac)), 35.10 (1C, s, -***C***H_2-(NPE)), 60.45 (1C, s, ***C***(5')), 67.06 (1C, s, -***C***H_2-(NPE)), 69.37 (1C, s, ***C***(3')), 82.53 (2C, d, J=7.89 Hz, ***C***(2'), ***C***(4')), 87.04 (1C, s, ***C***(1')), 118.35 (1C, s, ***C***(5')), 123.89 (2C, s, ***C***H-(NPE)), 130.02 (2C, s, ***C***H-(NPE)), 139.81 (1C, s, ***C***(8)), 145.60 (1C, s, ***C***(NPE)), 147.01 (1C, s, ***C***(NPE)), 152.25 (2C, d, J=15.90 Hz, ***C***(2), ***C***(4)), 160.72 (1C, s, ***C***(6)), 171.19 (1C, s, -***C***O-(Ac)), 215.19 (1C, s, -***C***S_2-).

^{1}H-NMR (400 MHz, $CDCl_3$, 25 °C) of compound **G6**

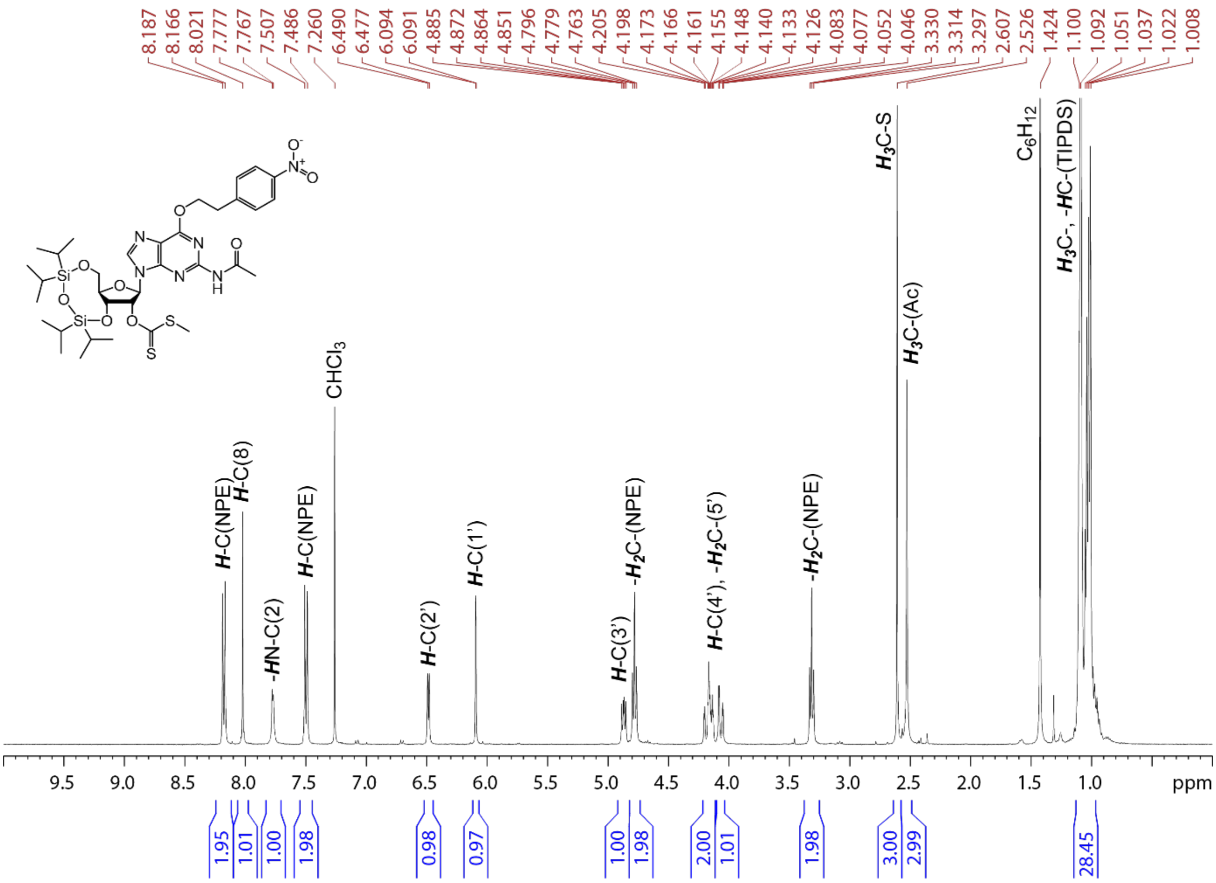

^{13}C-NMR (100 MHz, $CDCl_3$, 25 °C) of compound **G6**

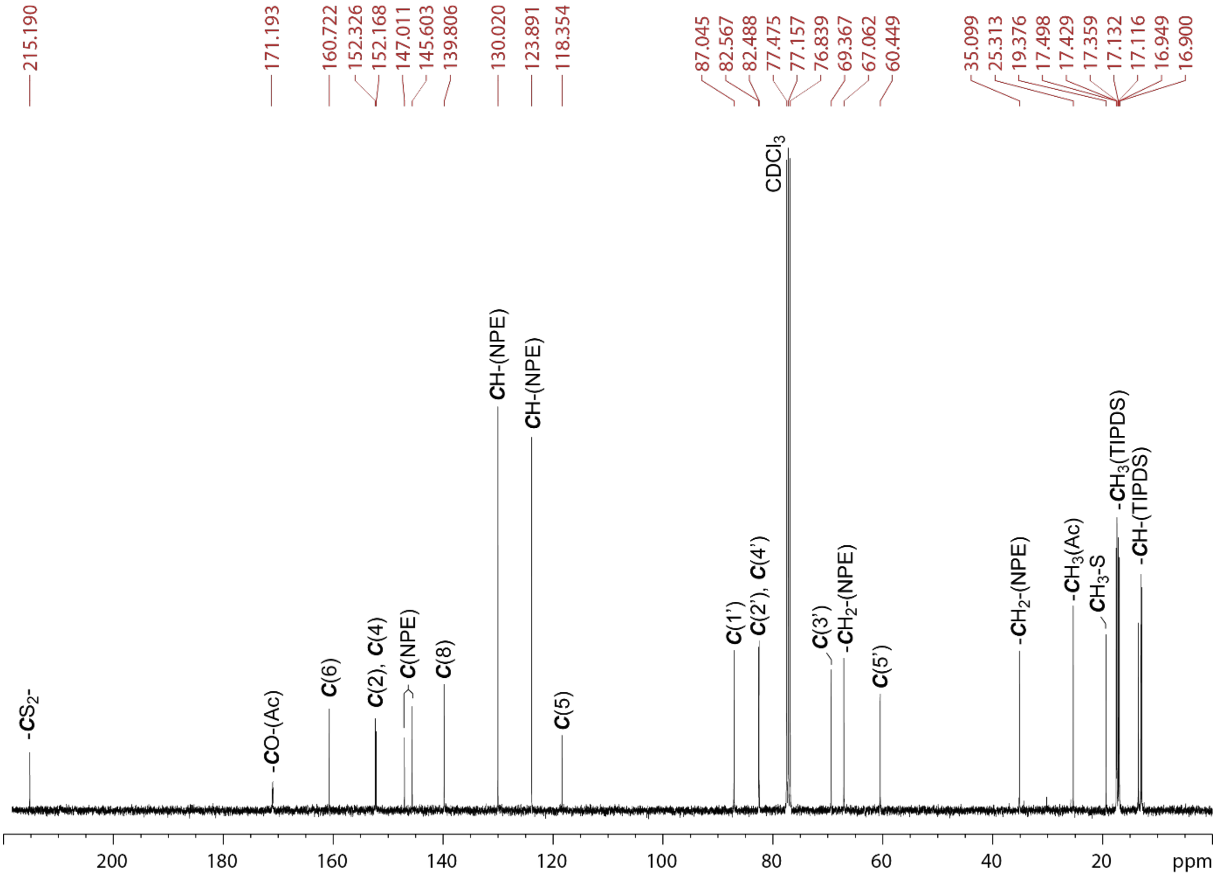

***N*^{2}-Acetyl-*O*^{6}-[2-(4-nitrophenyl)ethyl]-2'-*O*-(trifluoromethyl)guanosine (G7)**

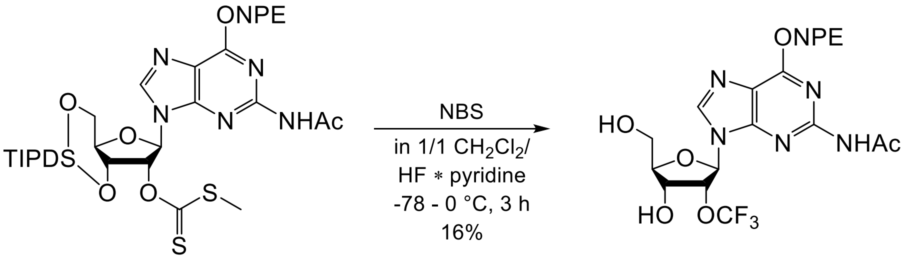

G6 **G7**

To a suspension of *N*-bromosuccinimide (NBS, 3.91 g, 22.0 mmol) in dry dichloromethane (8 mL) was added HF pyridine complex (70%, 24.0 mL, 26.40 g, 1.32 mol) at -78 °C. After stirring for 5 minutes a solution of compound **G6** (3.55 g, 4.40 mmol) in dichloromethane (13 mL) was added and the reaction was stirred for 3 h at 0 °C. Upon completion, the reaction mixture was carefully added to a stirring solution of saturated sodium bicarbonate solution (500 mL) and saturated sodium thiosulfate solution (200 mL). The aqueous phase was extracted 4 times with chloroform/isopropanol (3/1, 250 mL), organic phases were combined, washed with water, dried over sodium sulfate, filtered and evaporated. Residues were purified by column chromatography on silica gel using 2 – 10% methanol in dichloromethane as eluent. Yield: 377 mg of compound **G7** as a white solid (16%). TLC (dichloromethane/methanol, 9/1): R_F = 0.48. ESI-MS: (m/z) $[M+H]^+$ calcd. 543.1446; found: 543.1442. ^{1}H-NMR (400 MHz, DMSO-*d*$_6$, 25 °C, δ [ppm]): δ = 2.22 (3H, s, ***H*$_3$**C-(Ac)), 3.31 (2H, d, J=6.80 Hz, -***H*$_2$**C-(NPE)), 3.66 (2H, tdd, J=4.97, 11.96, 52.42 Hz, -***H*$_2$**C-(5')), 4.02 (1H, q, J=3.93 Hz, ***H***-C(4')), 4.48 (1H, q, J=4.52 Hz, ***H***-C(3')), 4.79 (2H, t, J=6.82 Hz, -***H*$_2$**C-(NPE)), 5.13 (1H, t, J=5.48 Hz, ***H***O-C(5')), 5.53 (1H, t, J=5.52 Hz, ***H***-C(2')), 5.97 (1H, d, J=5.40 Hz, ***H***O-C(3')), 6.22 (1H, d, J=6.00 Hz, ***H***-C(1')), 7.65 (2H, d, J=8.72 Hz, ***H***-C(NPE)), 8.18 (2H, d, J=8.76 Hz, ***H***-C(NPE)), 8.49 (1H, s, ***H***-C(8)), 10.47 (1H, s, -***H***N-C(2)). ^{13}C-NMR (100 MHz, DMSO-*d*$_6$, 25 °C, δ [ppm]): δ = 24.69 (1C, s, -***C***H$_3$(Ac)), 34.18 (1C, s, -***C***H$_2$-(NPE)), 60.83 (1C, s, ***C***(5')), 66.55 (1C, s, -***C***H$_2$-(NPE)), 68.88 (1C, s, ***C***(3')), 78.36 (1C, s, ***C***(2')), 84.33 (1C, s, ***C***(1')), 85.94 (1C, s, ***C***(4')), 117.21 (1C, s, ***C***(5)), 121.09 (1C, d, J=255.68 Hz, -***C***F$_3$), 123.43 (2C, s, ***C***H-(NPE)), 130.34 (2C, s, ***C***H-(NPE)), 141.30 (1C, s, ***C***(8)), 146.37 (2C, d, J=11.90 Hz, ***C***(NPE)), 152.27 (1C, s, ***C***(2)), 152.74 (1C, s, ***C***(4)), 159.91 (1C, s, ***C***(6)), 168.98 (1C, s, -***C***O-(Ac)). ^{19}F-NMR (377 MHz, DMSO-*d*$_6$, 25 °C, δ [ppm]): δ = -57.56 (3F, s, ***F*$_3$**C-).

^{1}H-NMR (400 MHz, DMSO-d_6, 25 °C) of compound **G7**

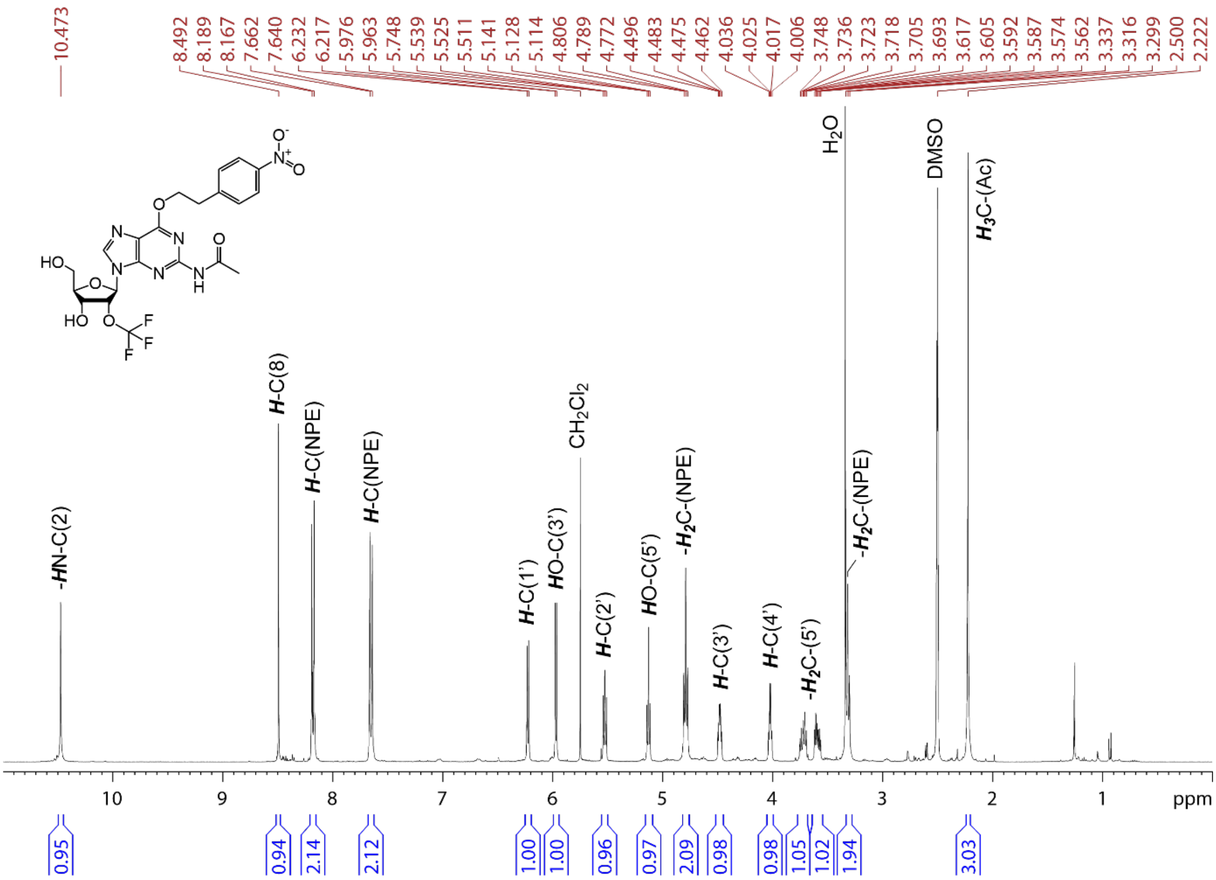

^{13}C-NMR (100 MHz, DMSO-d_6, 25 °C) of compound **G7**

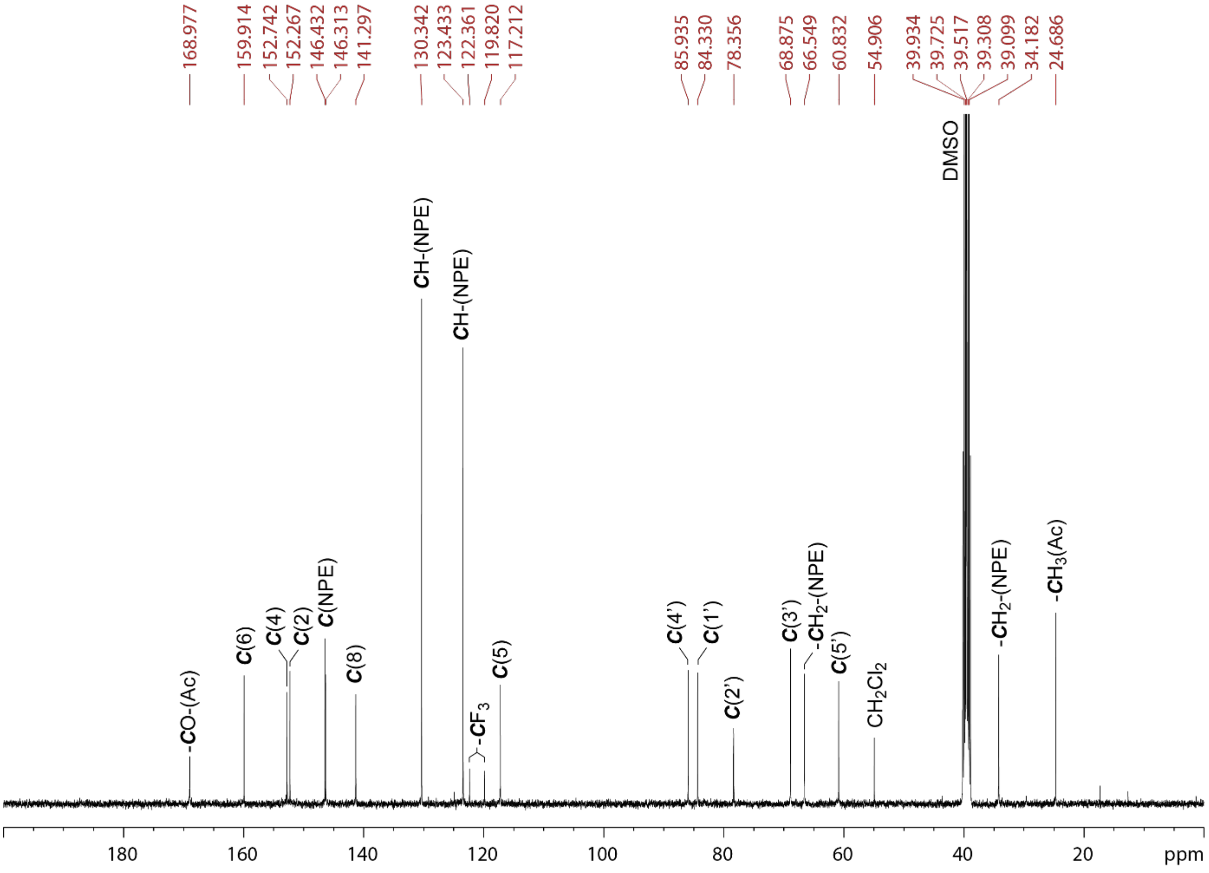

^{19}F-NMR (377 MHz, DMSO-d_6, 25 °C) of compound **G7**

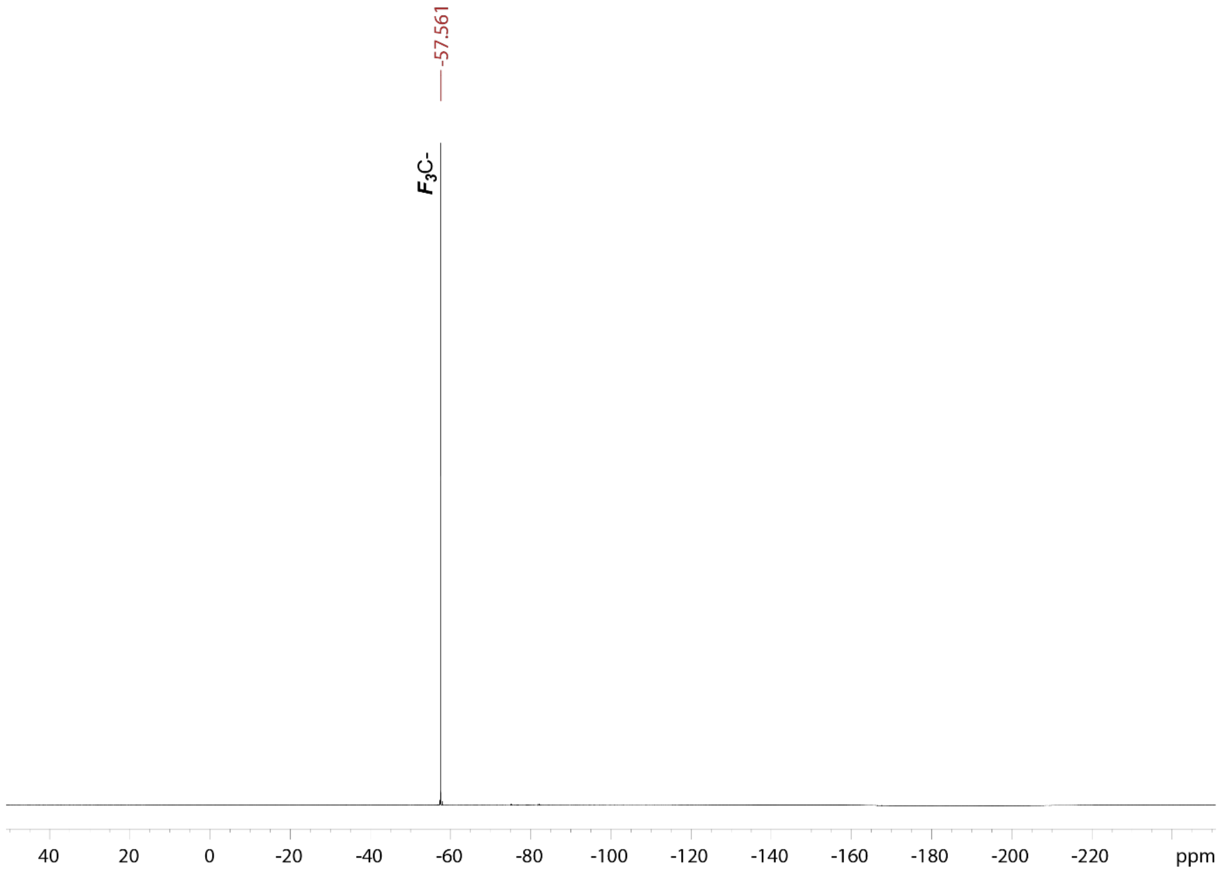

***N*²-Acetyl-*O*⁶-[2-(4-nitrophenyl)ethyl]-5'-*O*-(4,4'-dimethoxytrityl)-2'-*O*-(trifluoromethyl) guanosine (G8)**

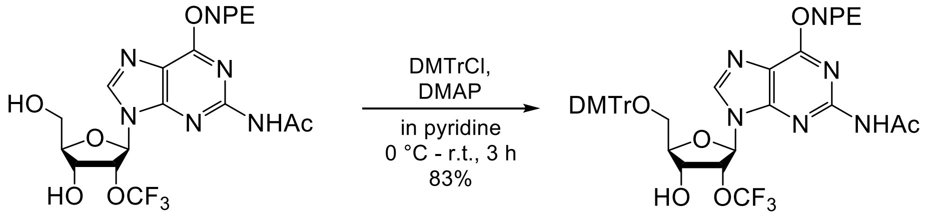

G7 **G8**

To a solution of compound **G7** (361 mg, 666 µmol) in dry pyridine (2.1 mL) was added 4,4'-dimethoxytrityl chloride (DMTrCl, 271 mg, 799 µmol) and dimethylaminopyridine (DMAP, 24.4 mg, 200 µmol) in three portions at 0 °C. The reaction was allowed to proceed at ambient temperatures for 3 hours while stirring. Upon complete conversion of starting material, the reaction was quenched by addition of methanol (0.1 mL) partitioned between ethyl acetate and water, washed with saturated sodium bicarbonate solution and brine. Organic phases were combined, dried over sodium sulfate, filtered, evaporated and coevaporated twice with toluene. Crude product was purified by column chromatography on silica gel using 30 - 60% ethyl acetate in hexanes as eluent. Yield: 466 mg of compound **G8** as a white foam (83%). TLC (hexanes/ethyl acetate, 1/2): R_F = 0.40. ESI-MS: (m/z) $[M+H]^+$ calcd. 845.2753; found: 845.2760. ^{1}H-NMR (400 MHz, $CDCl_3$, 25 °C, δ [ppm]): δ = 2.13 (3H, s, ***H*₃**C-(Ac)), 2.49 (1H, s, ***H***O-C(3')), 3.32 (2H, t, J=6.72 Hz, -***H*₂**C-(NPE)), 3.36 (1H, t, J=5.52 Hz, ***H(a)***-C(5')), 3.53 (1H, dd, J=2.44, 10.80 Hz, ***H(b)***-C(5')), 3.77 (6H, d, J=1.08 Hz, ***H*₃**C-O(DMTr)), 4.27 (1H, q, J=2.89 Hz, ***H***-C(4')), 4.75 (1H, d, J=3.64 Hz, ***H***-C(3')), 4.79 (2H, t, J=6.78 Hz, -***H*₂**C-(NPE)), 5.70 (1H, t, J=5.20 Hz, ***H***-C(2')), 6.17 (1H, d, J=6.04 Hz, ***H***-C(1')), 6.79 (4H, dd, J=1.08, 8.68 Hz, ***H***-C(DMTr)), 7.27 (7H, m, J=7.76 Hz), 7.41 (2H, d, J=6.80 Hz, ***H***-C(DMTr)), 7.51 (2H, d, J=8.64 Hz, ***H***-C(NPE)), 7.58 (1H, b, -***H***N-C(2)), 7.92 (1H, s, ***H***-C(8)), 8.17 (2H, d, J=8.64 Hz, ***H***-C(NPE)). ^{13}C-NMR (100 MHz, $CDCl_3$, 25 °C, δ [ppm]): δ = 24.80 (1C, s, -***C***H₃(Ac)), 35.02 (1C, s, -***C***H₂-(NPE)), 55.24 (2C, s, ***C***H₃-O(DMTr)), 63.17 (1C, s, ***C***(5')), 67.06 (1C, s, -***C***H₂-(NPE)), 70.38 (1C, s, ***C***(3')), 77.92 (1C, s, ***C***(2')), 83.92 (1C, s, ***C***(4')), 85.01 (1C, s, ***C***(1')), 86.87 (1C, s, ***C***(DMTr)), 113.29 (4C, s, ***C***H-(DMTr)), 118.38 (1C, s, ***C***(5)), 121.38 (1C, d, J=257.99 Hz, -***C***F₃), 123.81 (2C, s, ***C***H-(NPE)), 127.17 (1C, s, ***C***H-(DMTr)), 128.02 (4C, d, J=1.23 Hz, ***C***H-(DMTr)), 130.01 (6C, d, J=6.31 Hz, ***C***H-(DMTr), ***C***H-(NPE)), 135.39 (2C, d, J=23.09 Hz, ***C***(DMTr)), 140.69 (1C, s, ***C***(8)), 144.36 (1C, s, ***C***(DMTr)), 145.55 (1C, s, ***C***(NPE)), 146.94 (1C, s, ***C***(NPE)), 152.06 (1C, s, ***C***(2)), 152.76 (1C, s, ***C***(4)), 158.72 (2C, s, ***C***(DMTr)), 160.71 (1C, s, ***C***(6)). ^{19}F-NMR (377 MHz, $CDCl_3$, 25 °C, δ [ppm]): δ = -59.08 (3F, s, ***F*₃**C-).

^{1}H-NMR (400 MHz, $CDCl_3$, 25 °C) of compound **G8**

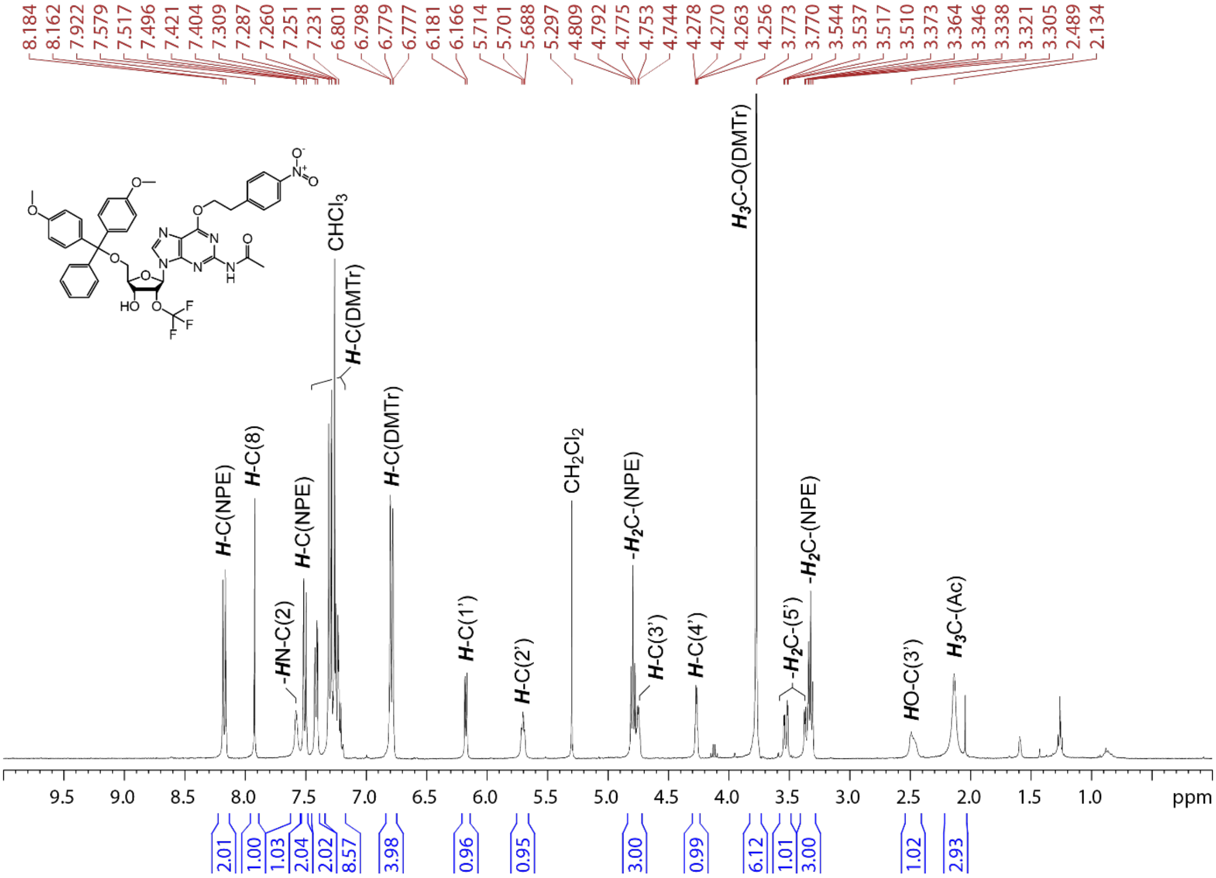

^{13}C-NMR (100 MHz, $CDCl_3$, 25 °C) of compound **G8**

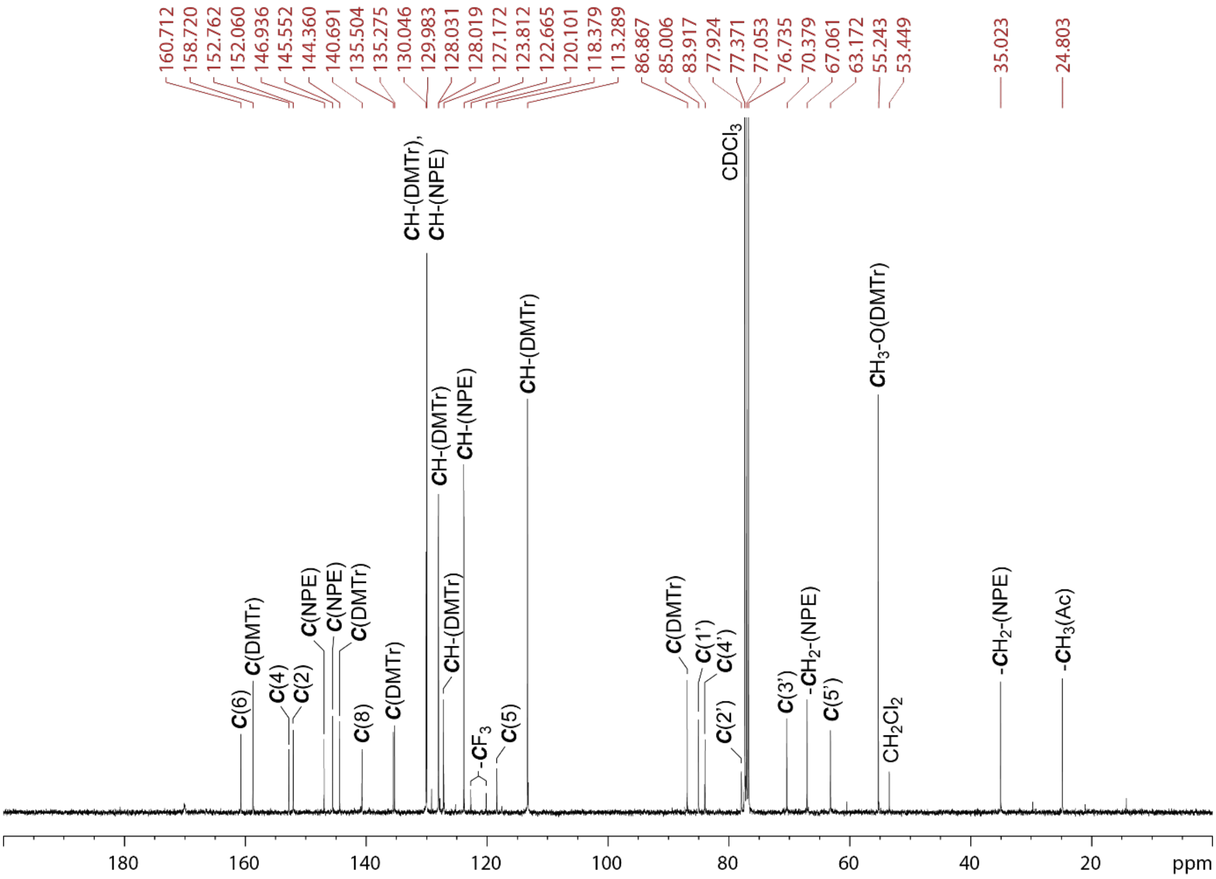

^{19}F-NMR (377 MHz, $CDCl_3$, 25 °C) of compound **G8**

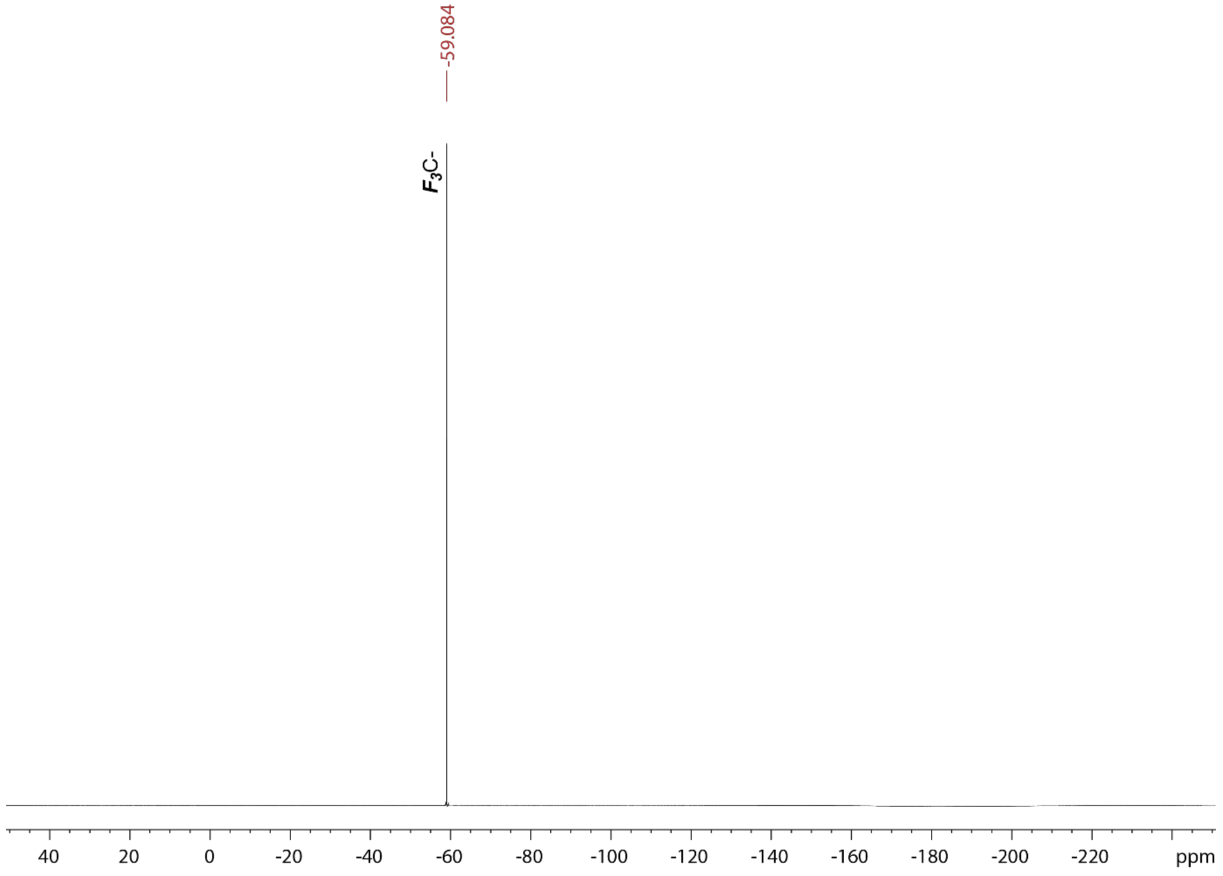

N^2-Acetyl-O^6-[2-(4-nitrophenyl)ethyl]-5'-*O*-(4,4'-dimethoxytrityl)-2'-*O*-(trifluoromethyl) guanosine 3'-(2-cyanoethyl)-*N*,*N*-diisopropylphosphoramidite (G9)

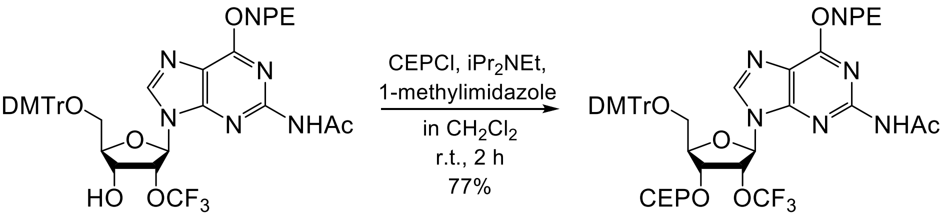

G8 **G9**

To a solution of compound **G8** (447 mg, 529 µmol) in dry dichloromethane (1.7 mL) was added diisopropylethylamine (691 µL, 513 mg, 3.97 mmol), 1-methylimidazole (21.0 µL, 21.7 mg, 265 µmol) and 2-cyanoethyl *N*,*N*-diisopropylchlorophosphoramidite (CEPCl, 295 µL, 313 mg, 1.32 mmol) at ambient temperatures. After stirring for 2 hours, TLC showed complete conversion of the starting material and the reaction was quenched by addition of methanol (0.1 mL), partitioned between dichloromethane and water and washed with saturated sodium bicarbonate solution. Organic phases were combined, dried over sodium sulfate, filtered and evaporated. Crude product was purified by column chromatography on silica gel, eluting with 20 - 60% ethyl acetate in hexane + 2% triethylamine. Yield: 424 mg of compound **G9** as a white foam and a 1/1 mixture of two diastereomers (77%). TLC (hexane/ethyl acetate, 1/1): R_F = 0.32 & 0.36. ESI-MS: (m/z) $[M+H]^+$ calcd. 1045.3831; found: 1045.3832. ^{1}H-NMR (400 MHz, $CDCl_3$, 25 °C, δ [ppm]): δ = 1.03 (3H, d, J=6.76 Hz, ***H*$_3$**C-CNP), 1.19 (9H, q, J=5.31 Hz, ***H*$_3$**C-CNP), 2.07 (3H, b, ***H*$_3$**C-(Ac)), 2.36 (1H, q, J=7.04 Hz, ***H(a)***-CCOP), 2.62 (1H, t, J=6.20 Hz ***H(b)***-CCOP), 3.28 (1H, t, J=5.52 Hz, ***H(a)***-C(5')), 3.33 (2H, t, J=6.70 Hz, -***H*$_2$**C-(NPE)), 3.52 (1H, q, J=4.27 Hz, ***H(b)***-C(5')), 3.60 (2H, m, J=3.56 Hz, -***H***C-NP), 3.69 (1H, m, J=5.02 Hz, ***H(a)***-COP), 3.78 (6H, q, J=1.53 Hz, ***H*$_3$**C-O(DMTr)), 3.86 (1H, m, J=4.22 Hz, ***H(b)***-COP), 4.36 (1H, dd, J=1.76, 40.21 Hz, ***H***-C(4')), 4.71 (1H, m, J=4.61 Hz, ***H***-C(3')), 4.80 (2H, m, J=3.23 Hz, -***H*$_2$**C-(NPE)), 5.72 (1H, td, J=5.23, 22.39 Hz, ***H***-C(2')), 6.16 (1H, dd, J=6.78, 22.83 Hz, ***H***-C(1')), 6.80 (4H, m, J=2.82 Hz, ***H***-C(DMTr)), 7.25 (3H, m, J=7.45 Hz, ***H***-C(DMTr)), 7.33 (4H, q, J=3.80 Hz, ***H***-C(DMTr)), 7.45 (2H, d, J=7.96 Hz, ***H***-C(DMTr)), 7.51 (2H, d, J=8.64 Hz, ***H***-C(NPE)), 7.56 (1H, s, -***H***N-C(2)), 7.94 (1H, d, J=6.40 Hz, ***H***-C(8)), 8.17 (2H, d, J=7.92 Hz, ***H***-C(NPE)). ^{13}C-NMR (100 MHz, $CDCl_3$, 25 °C, δ [ppm]): δ = 20.34 (1C, q, J=8.41 Hz, -***C***H_2-COP), 24.58 (4C, q, J=6.35 Hz, ***C***H_3-CNP), 24.83 (1C, d, J=4.04 Hz, -***C***H_3(Ac)), 35.14 (1C, s, -***C***H_2-(NPE)), 43.44 (1C, t, J=12.97 Hz, -***C***H-NP), 55.36 (2C, d, J=2.41 Hz, ***C***H_3-O(DMTr)), 58.33 (1C, q, J=43.52 Hz, -***C***H_2-OP), 63.06 (1C, d, J=17.35 Hz, ***C***(5')), 67.11 (1C, s, -***C***H_2-(NPE)), 71.56 (1C, dd, J=16.54, 130.88 Hz, ***C***(3')), 84.35 (1C, d, J=71.61 Hz, ***C***(4')), 85.23 (1C, d, J=24.21 Hz, ***C***(1')), 87.00 (1C, d, J=3.81 Hz, ***C***(DMTr)), 113.41 (4C, s, ***C***H-(DMTr)), 117.47 (1C, d, J=25.60 Hz, -***C***N), 118.51 (1C, d, J=5.84 Hz, ***C***(5)), 121.51 (1C, d, J=252.53 Hz, -***C***F_3), 123.90 (2C, s, ***C***H-(NPE)), 127.32 (1C, s, ***C***H-(DMTr)), 128.15 (4C, t, J=4.44 Hz, ***C***H-(DMTr)), 130.13 (6C, t, J=6.47 Hz, ***C***H-(DMTr), ***C***H-(NPE)), 135.48 (2C, q, J=11.82 Hz, ***C***(DMTr)), 140.68 (1C, d, J=10.85 Hz, ***C***(8)), 144.44 (1C, d, J=7.26 Hz, ***C***(DMTr)), 145.71 (1C, s, ***C***(NPE)), 147.02 (1C, s, ***C***(NPE)), 152.23 (1C, d, J=2.38 Hz, ***C***(2)), 152.96 (1C, d, J=6.19 Hz, ***C***(4)), 158.85 (2C, s, ***C***(DMTr)), 160.79 (1C, d, J=2.79 Hz, ***C***(6)). ^{19}F-NMR (377 MHz, $CDCl_3$, 25 °C, δ [ppm]): δ = -59.15 (3F, dd, J=5.05, 90.17 Hz, ***F*$_3$**C-). ^{31}P-NMR (162 MHz, $CDCl_3$, 25 °C, δ [ppm]): δ = 152.00 (1P, dd, J=4.95, 74.56 Hz, ***P***-OC(3')).

^{1}H-NMR (400 MHz, $CDCl_3$, 25 °C) of compound **G9**

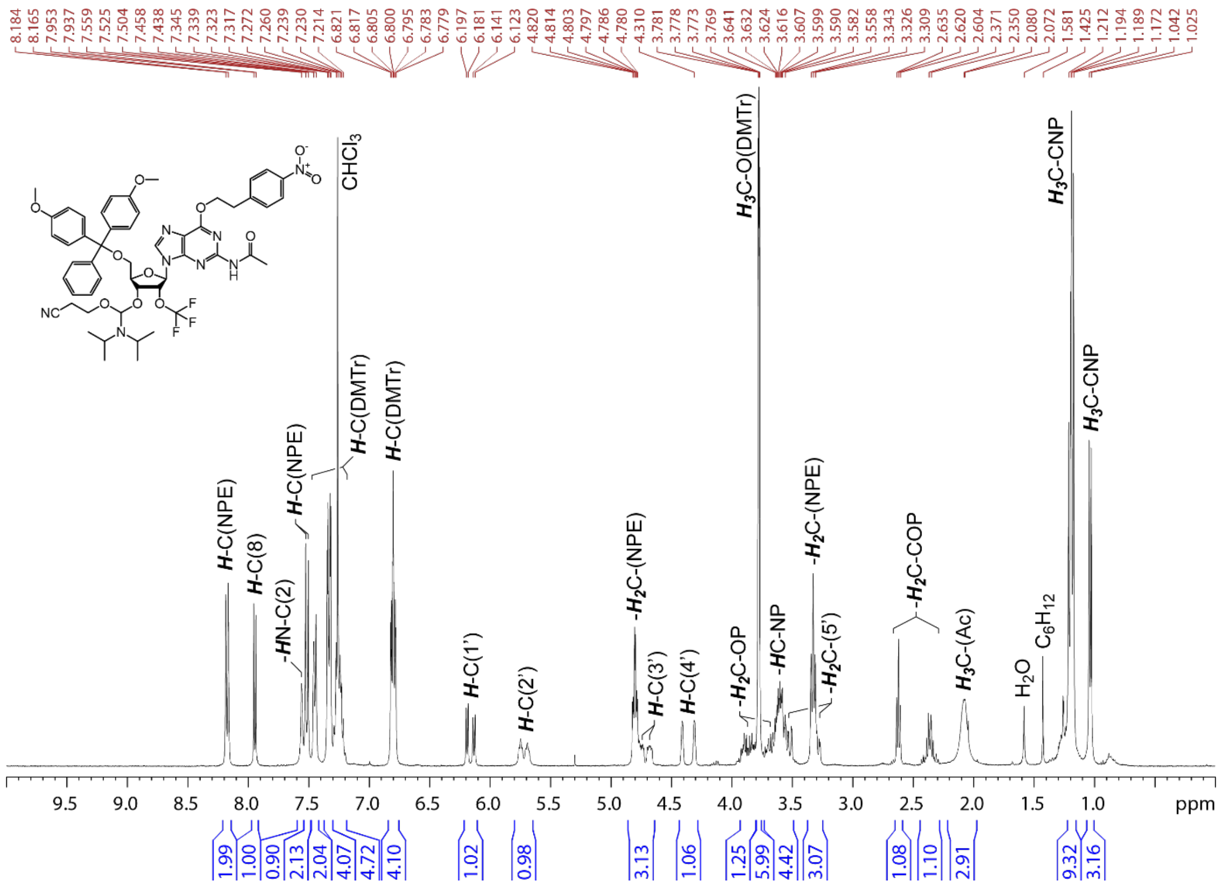

^{13}C-NMR (100 MHz, $CDCl_3$, 25 °C) of compound **G9**

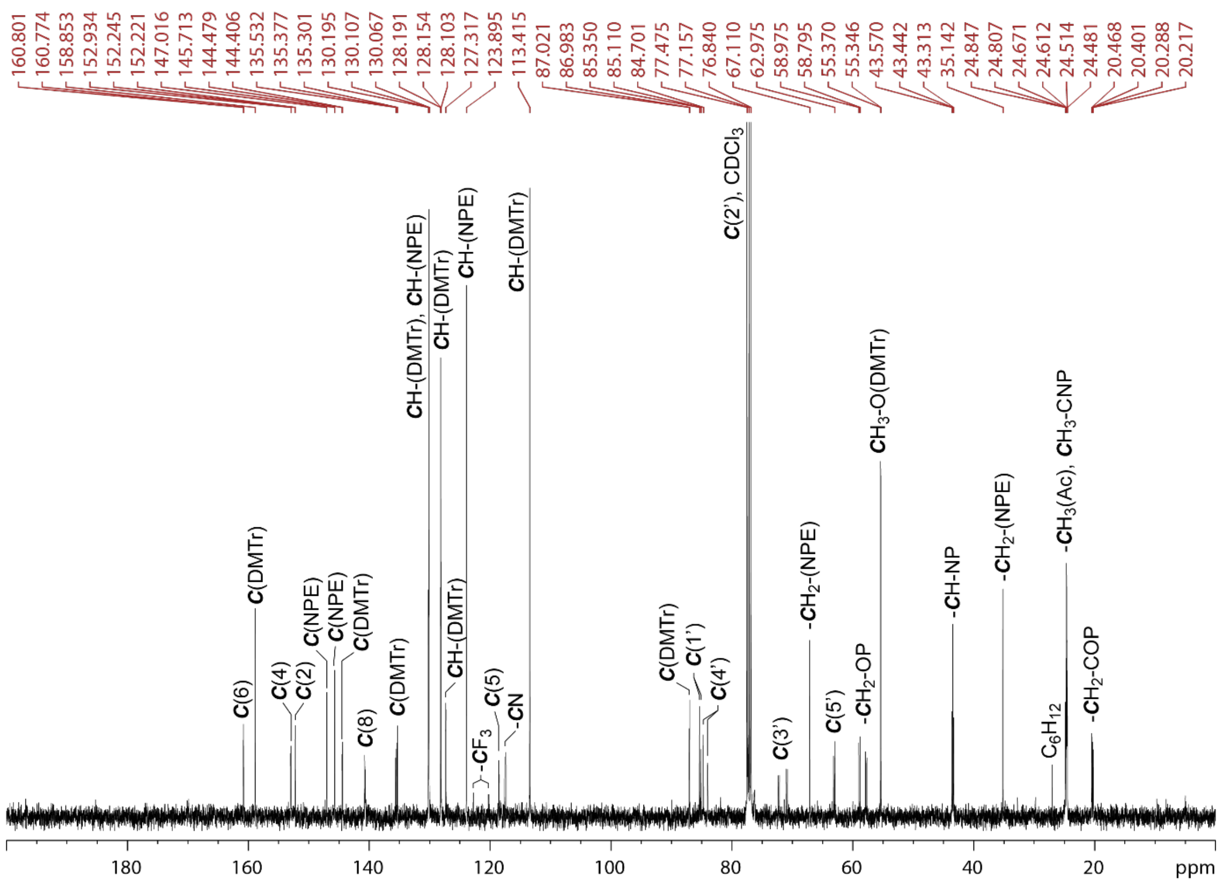

^{19}F-NMR (377 MHz, $CDCl_3$, 25 °C) of compound **G9**

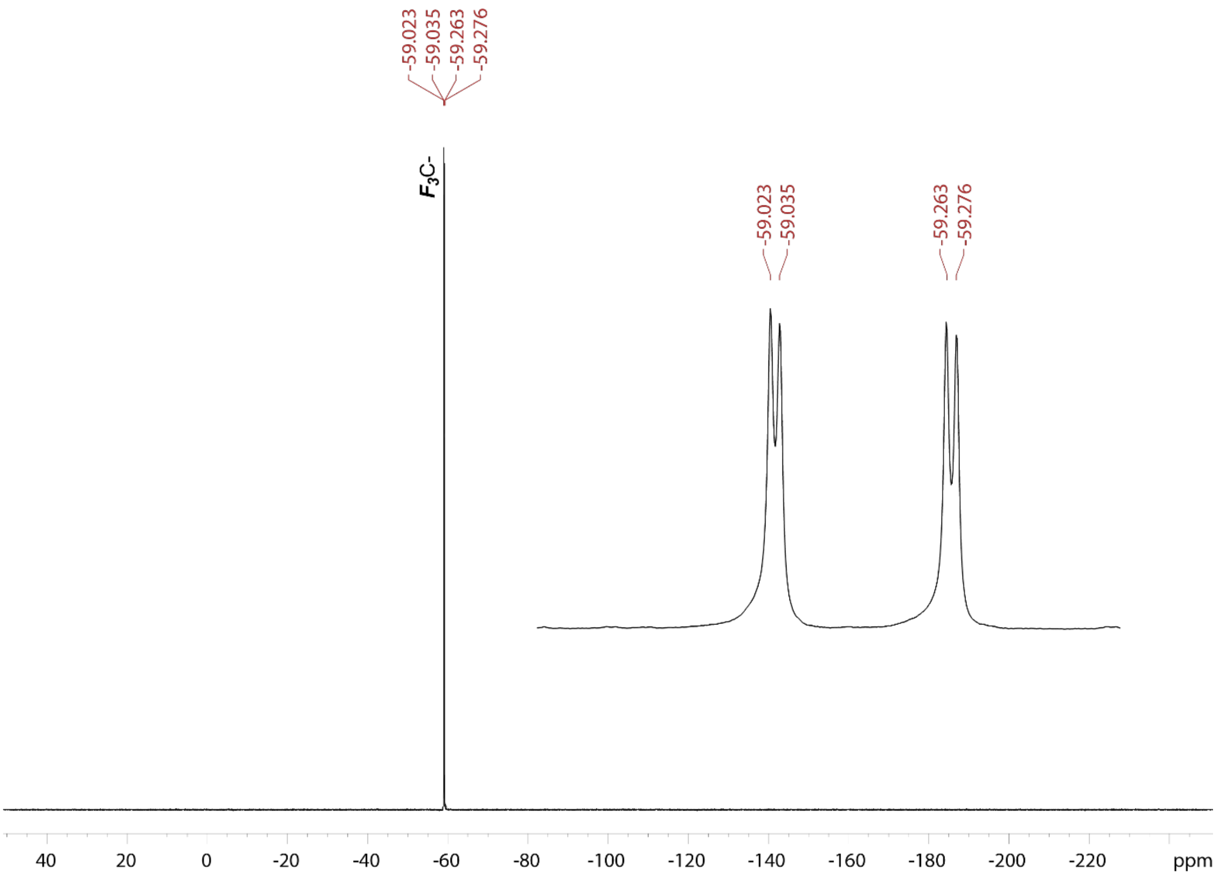

^{31}P-NMR (162 MHz, $CDCl_3$, 25 °C) of compound **G9**

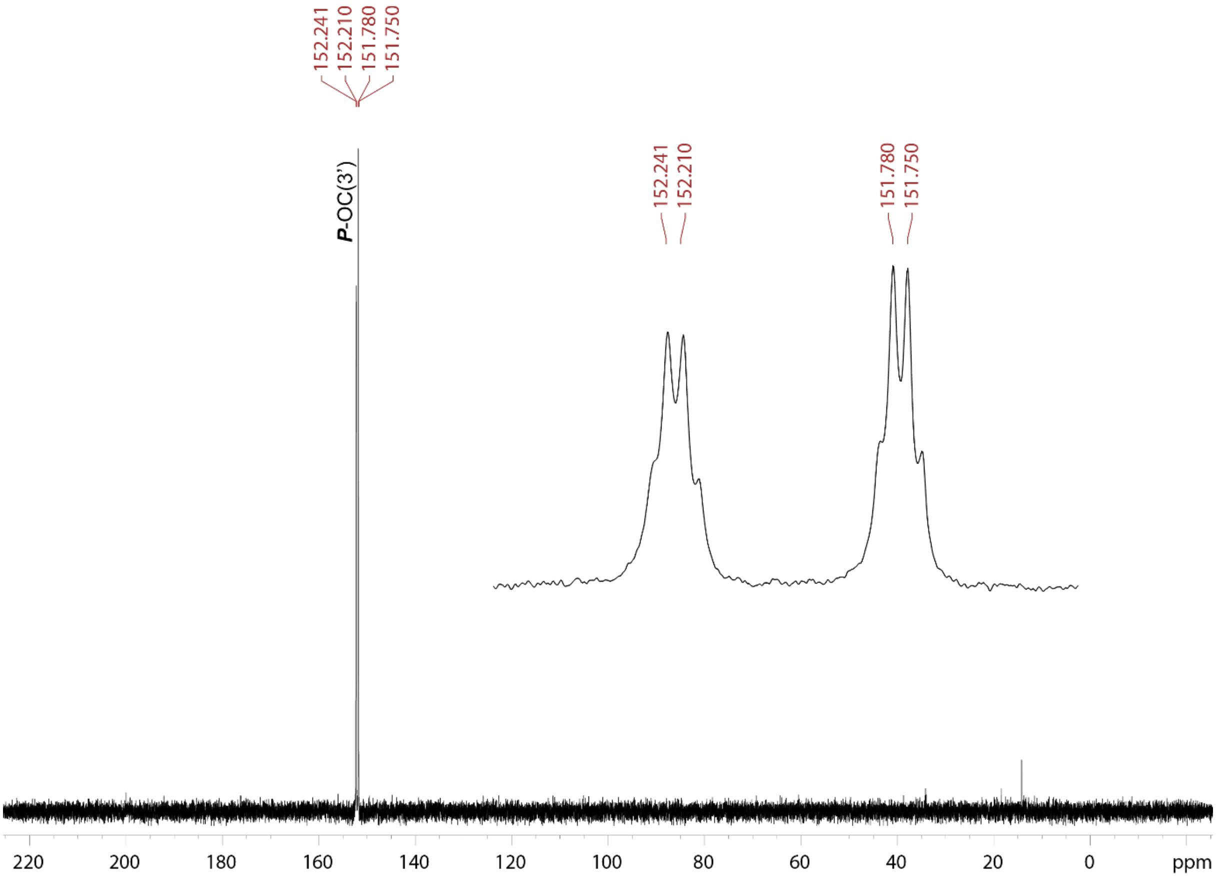

Synthesis of 2'-OCF_3 uridine phosphoramidite U3

2'-*O*-(Trifluoromethyl)uridine (U1)

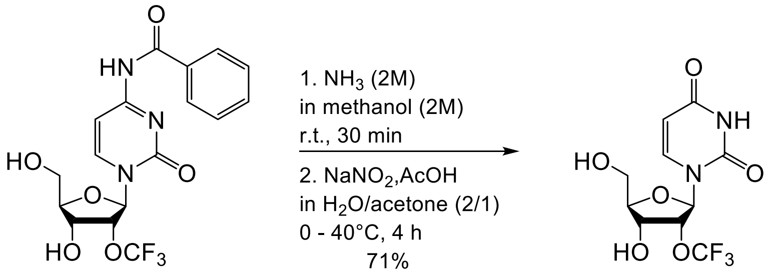

C2 **U1**

Compound **C2** (858 mg, 2.07 mmol) was dissolved in methanolic ammonia (2 M, 21 mL) and stirred for 30 min. before all volatiles were removed. The obtained residue was combined with sodium nitrite (4.72 g, 61.9 mmol) and suspended in 21 mL of water/acetone (2/1) and cooled to 0 °C. Acetic acid (AcOH, 4.72 mL, 61.9 mmol) was added dropwise over a period of 30 min and continuously stirred for 1 h at 0 °C before heating the reaction mixture to 40 °C for 2.5 h. The reaction mixture was neutralized using saturated $NaHCO_3$ solution, diluted with water until all solids were dissolved and extracted several times with chloroform/isopropanol (3/1). The combined organic phases were dried using sodium sulfate, filtered through a cotton plug before removing all volatiles. The crude product was purified by column chromatography on silica gel using 5 - 15% methanol in dichloromethane as eluent. Yield: 459 mg of compound **U1** as a white foam (71%). TLC (dichloromethane/methanol, 9/1): R_F = 0.32. ESI-MS: (m/z) $[M+H]^+$ calcd. 313.0642 found: 313.0624. ^{1}H-NMR (400 MHz, DMSO-*d*$_6$, 25 °C, δ [ppm]): δ = 3.63 (2H, m, ***H*₂**-C(5')), 3.95 (1H, q, J=7.84 Hz, J=3.11 Hz, ***H***-C(4')), 4.23 (1H, q, J=11.04 Hz, J=4.99 Hz, ***H***-C(2')), 4.88 (1H, t, J=5.28 Hz, ***H***-C(2')), 5.30 (1H, t, J=4.90 Hz, ***H***O-C(5'), 5.71 (1H, d J=8.10 Hz, **H**-C(5)), 5.86 (1H, d, J=5.75 Hz, ***H***O-C(3'), 6.06 (1H, d, J=5.39 Hz, H-(C1')), 7.93 (1H, d, J=8.14 Hz, H-C(6)) , 11.43 (1H, s, ***H***-N(3)) .^{13}C-NMR (100 MHz, DMSO-*d*$_6$, 25 °C, δ [ppm]): δ 60.2 (1C, s, ***C***(5')), 68.2 (1C, s, ***C***(3')), 78.8 (1C, s, ***C***(2')), 85.0 (2C, s, ***C***(2'), ***C***(4')), 102.4 (1C, s, ***C***(5)), 121.1 (1C, d, J=255.1 Hz, -***C***F_3), 140.0 (1C, s, ***C***(6)), 150.5 (1C, s, ***C***(2)), 162.9 (1C, s, ***C***(4)). ^{19}F-NMR (376 MHz, DMSO-d$_6$, 25°C, δ = [ppm]): δ -57.32 (3F, s, ***F***$_3$C-).

^{1}H-NMR (400 MHz, DMSO-d_6, 25 °C) of compound **U1**

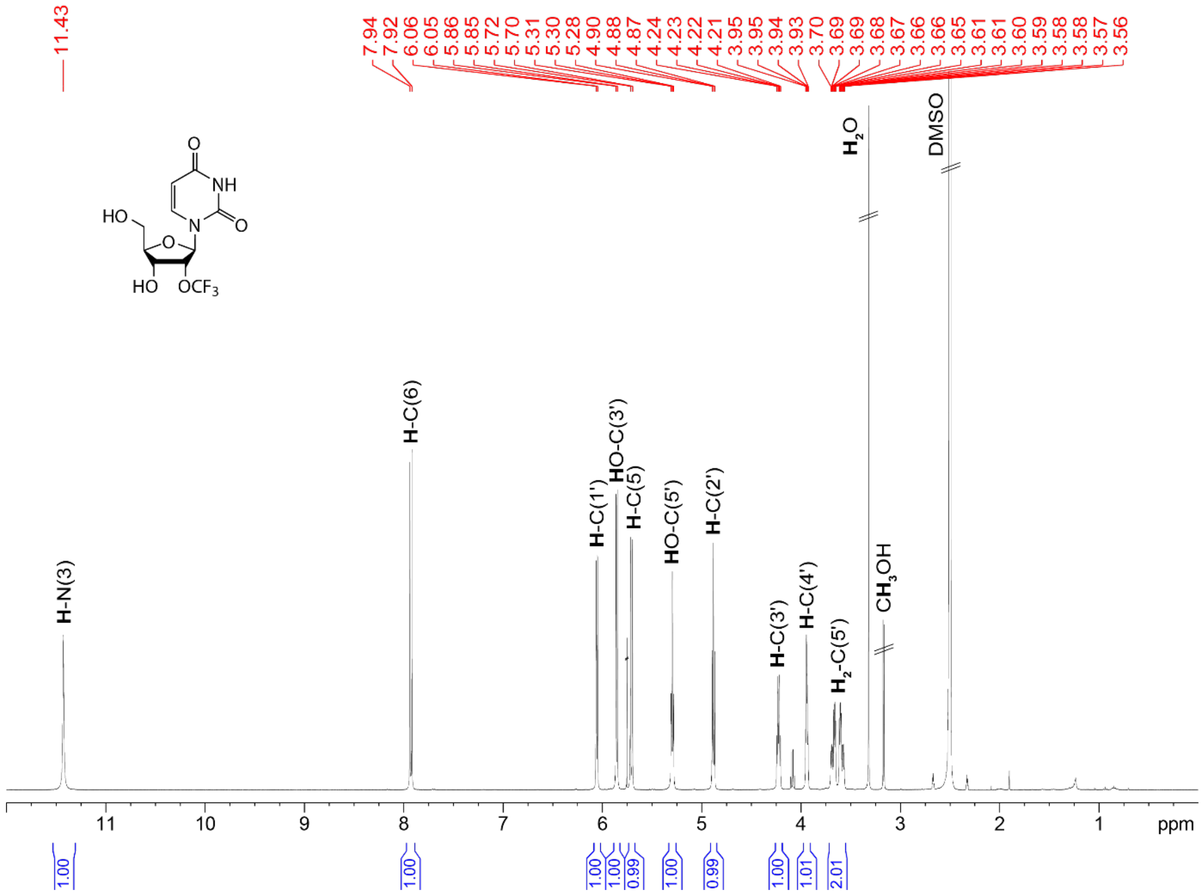

^{13}C-NMR (100 MHz, DMSO-d_6, 25 °C) of compound **U1**

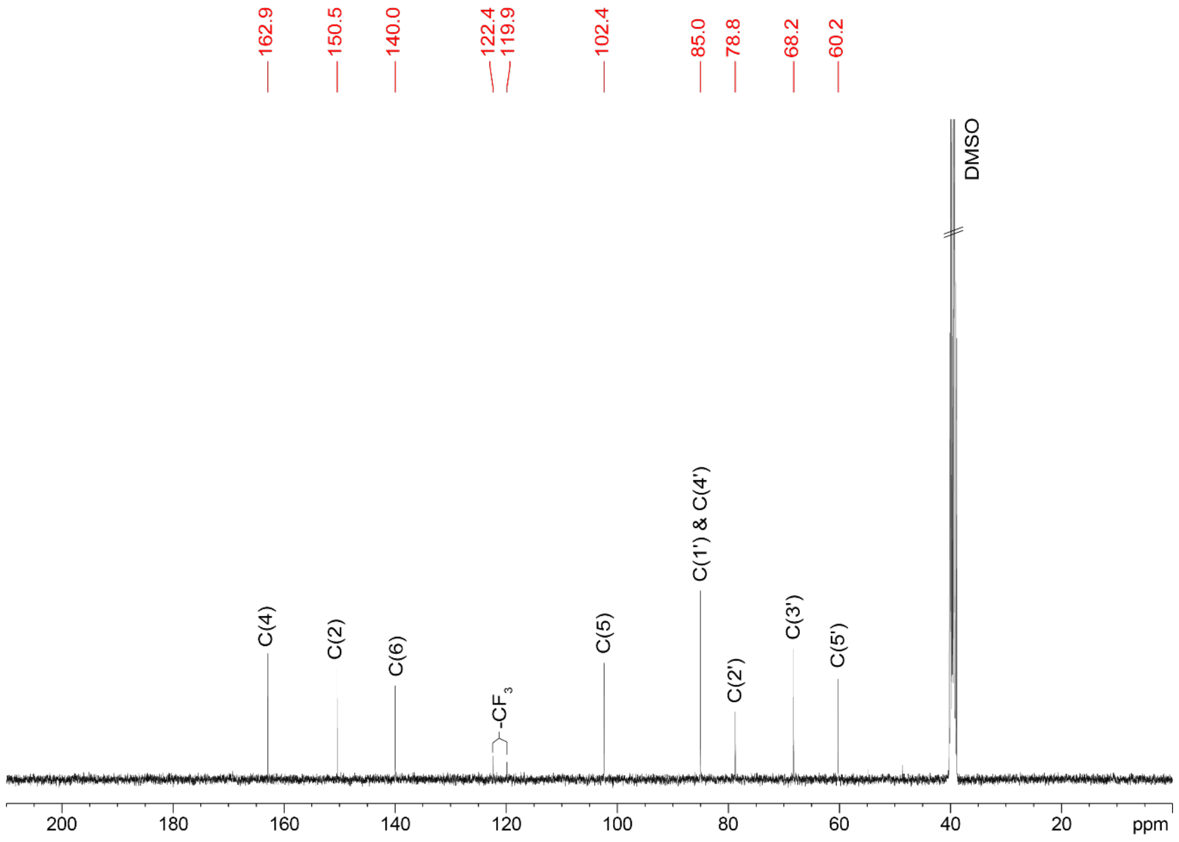

^{19}F-NMR (377 MHz, DMSO-d_6, 25 °C) of compound **U1**

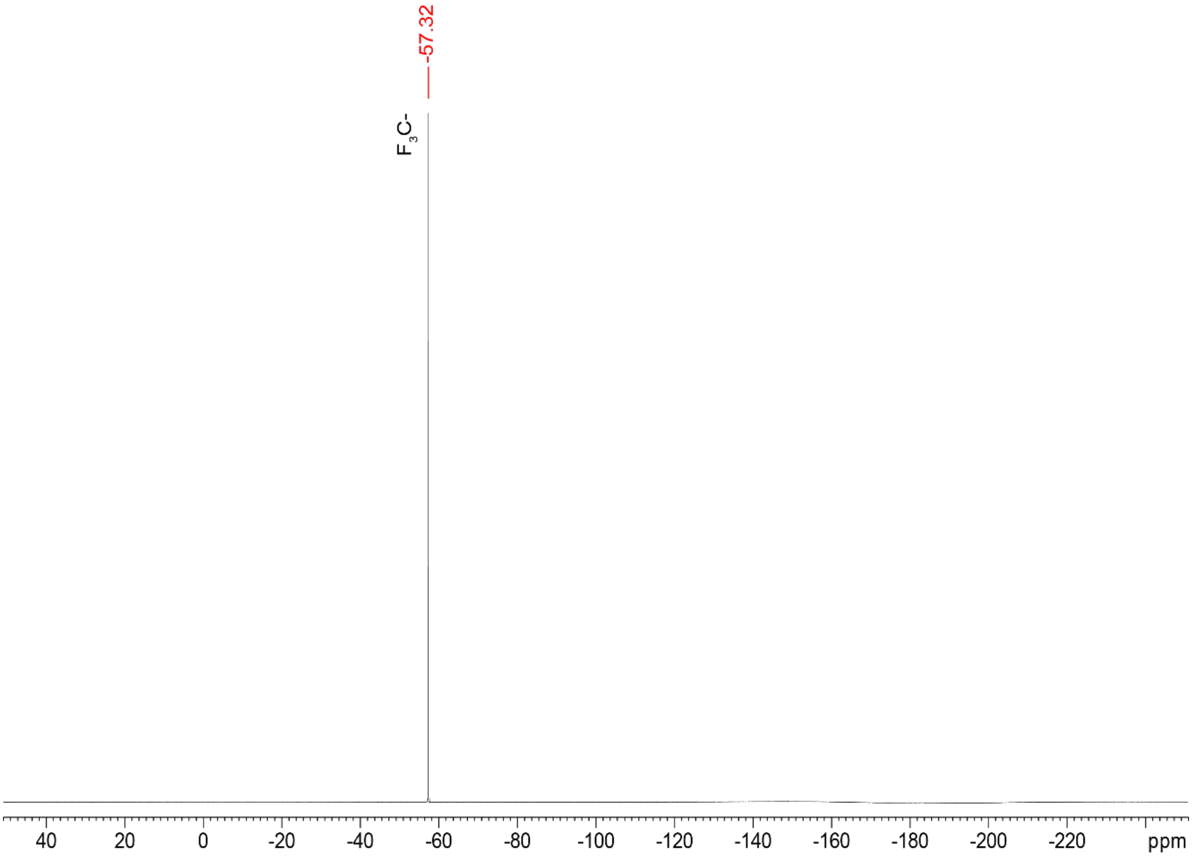

5'-*O*-(4,4'Dimethoxytrityl)-2'-*O*-(trifluoromethyl)uridine (U2)

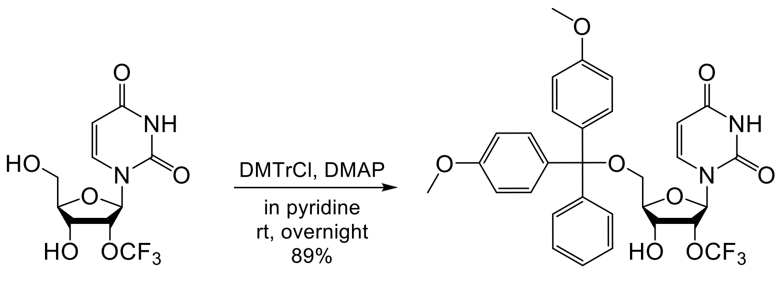

U1 **U2**

Compound **U1** (461 mg, 1.48 mmol) and 4-dimethylaminopyridine (DMAP, 461 mg, 1.48 mmol) were dissolved in pyridine (15 mL). 4,4'-dimethoxytrityl chloride (DMTrCl, 770 mg, 2.27 mmol) was added in portions over a period of 30 min and continuously stirred overnight. The reaction was quenched by the addition of methanol (2 mL) before removing all volatiles and co-evaporating twice with toluene. The residue was dissolved in dichloromethane, extracted twice with 5% citric acid solution, once with saturated sodium bicarbonate and saturated sodium chloride solution. The organic phase was dried using sodium sulfate, filtered through a cotton plug before removing all volatiles. The crude product was purified by column chromatography using 0 – 7% methanol in dichloromethane (2% triethyl-amine). Yield: 810 mg of compound **U2** as a white foam (81%). TLC (dichloromethane/methanol, (9/1): R_F = 0.52. ESI-MS: (m/z) $[M+Na]^+$ calcd. 637.1768 found: 637.1761. ^{1}H-NMR (400 MHz, DMSO-*d*$_6$, 25 °C, δ [ppm]): δ = 3.29 (2H, m, ***H***$_2$-C(5')), 3.74 (6H, s, ***H***$_3$C-ODMTr), 4.02 (1H, m, ***H***-C(4')), 4.34 (1H, q, J = 6.06 Hz, J = 5.76 Hz, ***H***-C(2')), 5.03 (1H, tripletoid, J = 4.73 Hz, ***H***-C(2')), 5.39 (1H, d J = 8.14 Hz, ***H***-C(5)), 5.89 (1H, d, J = 6.42 Hz, ***H***O-C(3'), 5.99 (1H, d, J = 3.91 Hz, H-(C1')), 6.90 (4H, m, ***H***-C(DMTr), 7.25 (5H, m, ***H***-C(DMTr), 7.32 (2H, m, **H**-C(DMTr), 7.38 (2H, m, **H**-C(DMTr), 7.73 (1H, d, J = 8.20 Hz, H-C(6)) , 11.43 (1H, bs, ***H***-N(3)).^{13}C-NMR (100 MHz, DMSO-*d*$_6$, 25 °C, δ [ppm]): δ = 55.1 (***C***H_3-O(DMTr)), 62.4 (1C, s, ***C***(5')), 67.7 (1C, s, ***C***(3')), 78.7 (1C, s, ***C***(2')), 82.3 (1C, s, ***C***(4')), 86.0 (1C, ***C***-(DMTr)), 86.6 (1C, ***C***(1 ')), 101.9 (1C, s, ***C***(5)), 113.3 (4C, s, ***C***H-(DMTr)), 123.2 (1C, d, J = 255.1 Hz, -***C***F_3), 126.8 (***C***H-(DMTr)), 127.7 (2C, s, ***C***H-(DMTr)), 127.9 (2C, s, ***C***H-(DMTr)), 129.8 (4C, s, ***C***H-(DMTr)), 135.0 (1C, s, ***C***-(DMTr)), 135.3 (1C, s, ***C***-(DMTr)), 140.3 (1C, s, ***C***(6)), 144.6 (1C, s, ***C***-DMTr), 150.2 (1C, s, ***C***(2)), 158.2 (2C, s, ***C***-DMTr), 162.9 (1C, s, ***C***(4)).^{19}F-NMR (376 MHz, DMSO-d$_6$, 25°C, δ [ppm]): δ = -56.99 (3F, s, ***F***$_3$C-).

^{1}H-NMR (400 MHz, DMSO-d_6, 25 °C) of compound **U2**

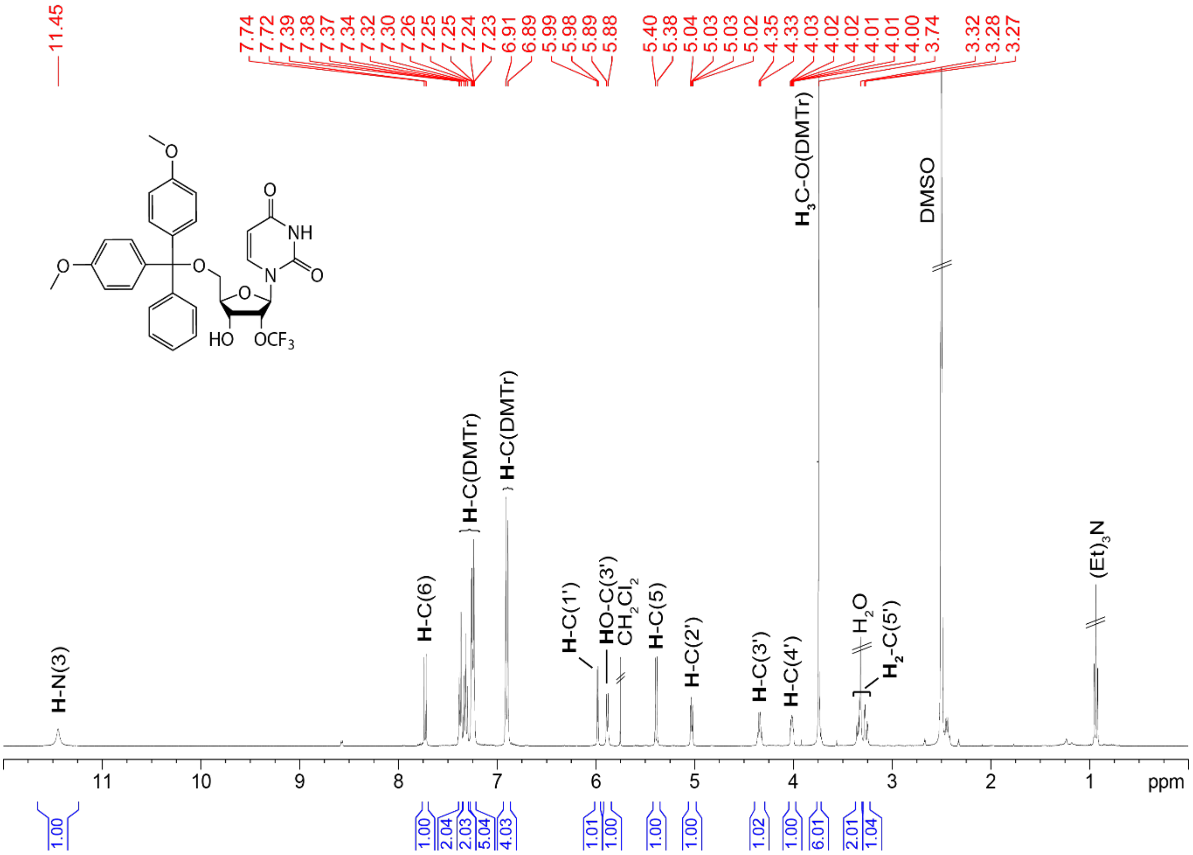

^{13}C-NMR (100 MHz, DMSO-d_6, 25 °C) of compound **U2**

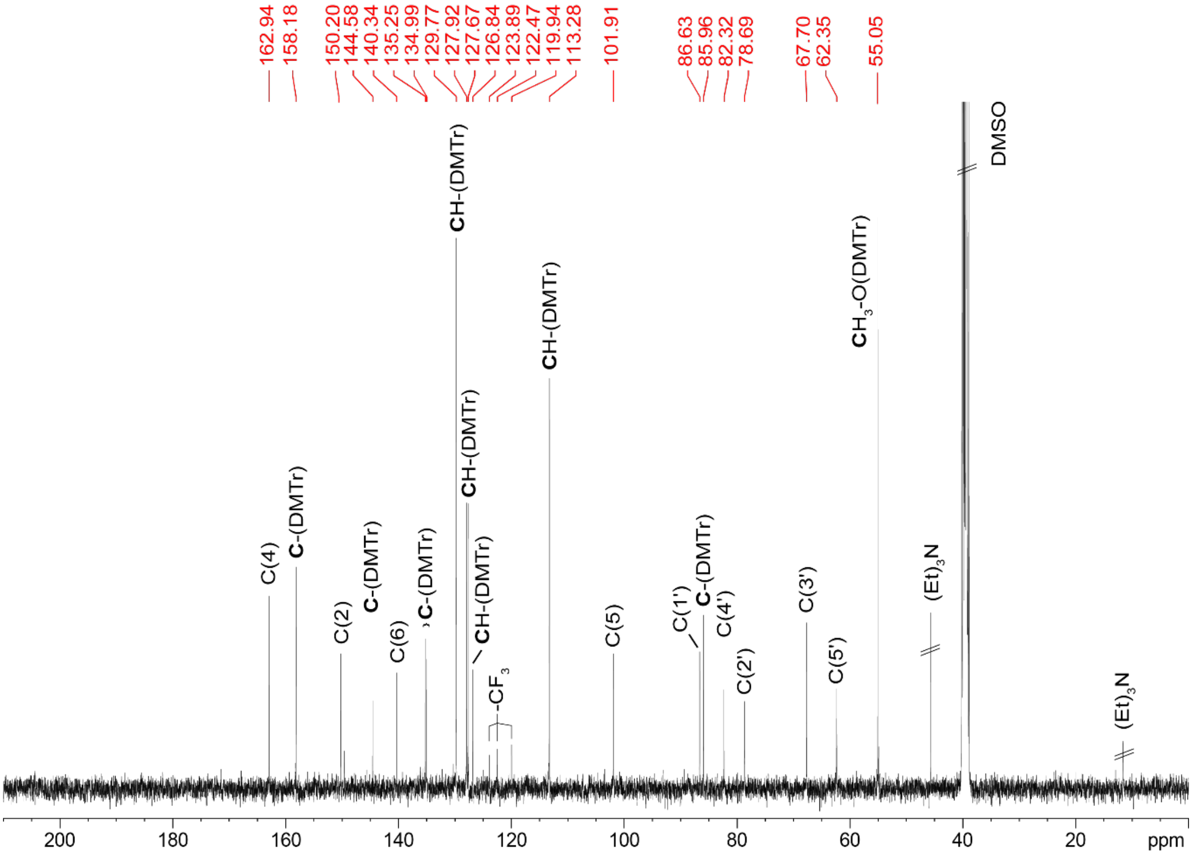

^{19}F-NMR (377 MHz, DMSO-d_6, 25 °C) of compound **U2**

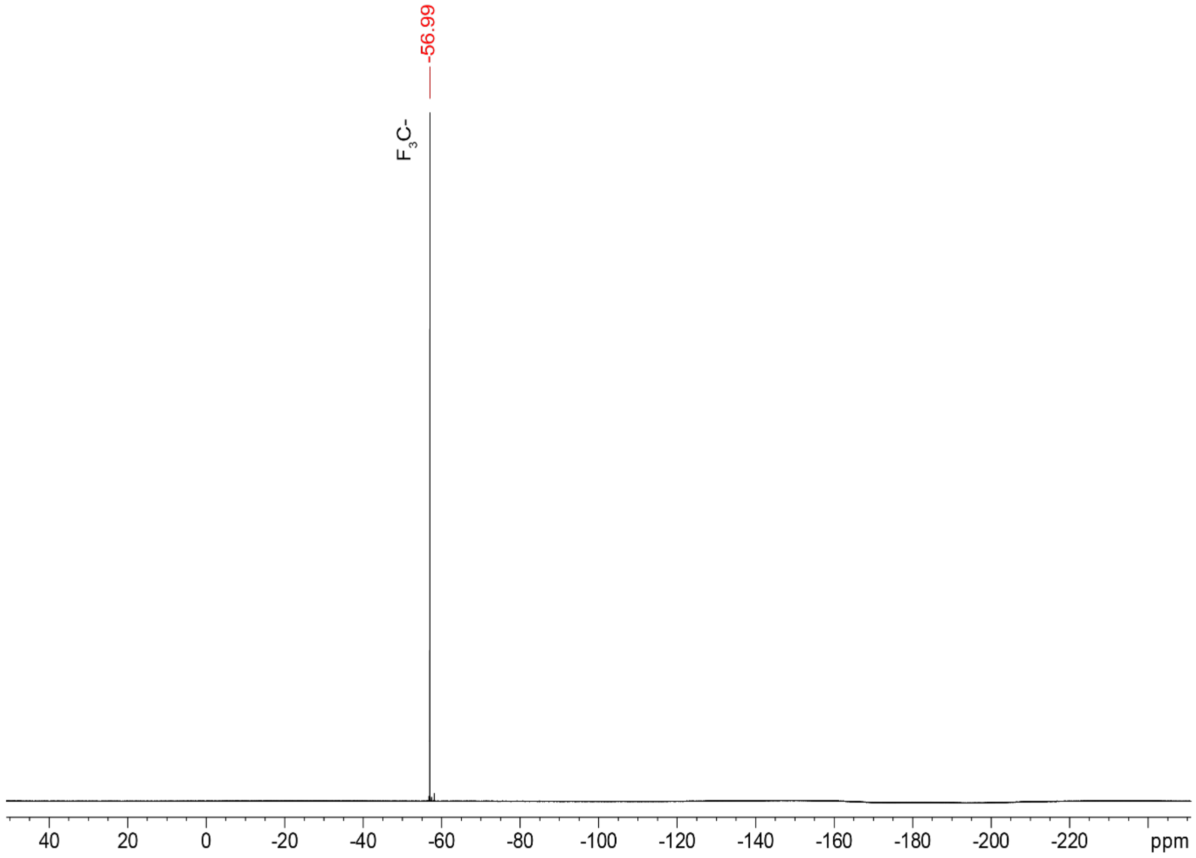

5'-*O*-(4,4'Dimethoxytrityl)-2'-*O*-(trifluoromethyl)uridine-3'-(2-cyanoethyl)-*N*,*N*-diisopropylphosphoramidite (U3)

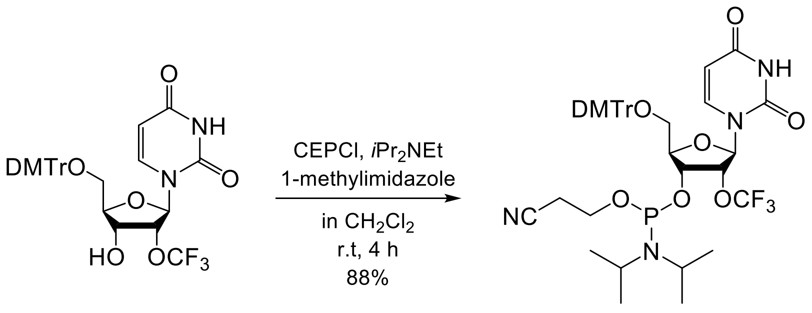

U2 **U3**

Compound **U2** (858 mg, 1.30 mmol) was dissolved in dichloromethane (13 mL) before adding *N*,*N*-diisopropylethylamine (905 µL, 671 mg, 5.20 mmol) and mixing the resulting solution for 15 min. Subsequently 1-methylimidazole (52 µL, 53 mg, 649 µmol) and 2-cyanoethyl *N*,*N*-diisopropylchloro-phosphoramidite (CEPCl, 580 µL, 615 mg, 2.60 mmol) were added to the solution and mixed for 4 h at room temperature. The reaction was quenched by the addition of methanol (0.5 mL), diluted with dichloromethane and washed with saturated sodium bicarbonate and saturated sodium chloride solutions. The organic phase was dried using sodium sulfate before removing all volatiles under reduced pressure. The crude product was purified by column chromatography on silica gel using 25% ethyl acetate in cyclohexane (2% triethylamine). Yield: 929 mg of a 4:3 mixture of diastereomers of compound **U3** as a white foam (88%). TLC (ethyl acetate/cyclohexane, (1/1): R_F = 0.45. ESI-MS: (m/z) $[M+H]^+$ calcd. 815.3027 found: 815.3009. ^{1}H-NMR (400 MHz, DMSO-*d*6, 25 °C, δ [ppm]): δ = 0.96–1.14 (12H, m, ***H*3**C-CNP), 2.57 (t, J = 5.93 Hz, ***H*2**C-COP(a)), 2.76 (t, J = 5.90 Hz, ***H*2**C-COP(b)), 3.33-3.42 (2H, m, ***H*2**-C(5')), 3.48-3.58 (2H, m, ***H***C-NP), 3.59-3.82 (2H, m, ***H*2**C-OP), 3.74 (3H, s, ***H*3**C-O(DMTr) a & b), 4.11-4.17 (1H, m, ***H***-C(4')), 4.49–4.56 (1H, m, ***H***-C(3')), 5.18 (1H, dd*,* J = 1.80 Hz, J = 3.69 Hz, ***H***-C(2'a)), 5.18 (1H, triplettoid*,* J = 7.49 Hz, ***H***-C(2'b)), 5.43 (1H, t, J = 7.53 Hz, ***H***-C(5)), 6.00 (1H, d, J = 4.12 Hz, ***H***-(C1')), 6.87-6.91 (4H, m, ***H***-C(DMTr), 7.23-7.34 (7H, m, ***H***-C(DMTr), 7.39 (2H, m, ***H***-C(DMTr), 7.77 (1H, d, J = 8.20 Hz, H-C(6)) , 11.48 (1H, bs, ***H***-N(3)). ^{13}C-NMR (100 MHz, DMSO-*d*6, 25 °C, δ [ppm]): δ = 20.2 (1C, t, J=7.55 Hz, -***C***H2-CNP), 24.5 -24.8 (4C, m, ***C***H3-O(DMTr)), 43.1 (2C, d J=12.64 Hz, -***C***H-NP)), 55.5 (***C***H3-O(DMTr)), 58.9 (1C, q, J=19.4 Hz, -***C***H2-OP), 62.4 (1C, d, J=5.1 Hz, ***C***(5')), 69.4 (1C, d, J=14.8 Hz, ***C***(3' a)), 70.0 (1C, d, J=14.8 Hz, ***C***(3' b)), 77.4 (1C, s, ***C***(2' a)), 78.3 (1C, s, ***C***(2' b)), 82.0 (1C, d, J=4.6 Hz, ***C***(4' a)), 82.6 (1C, s, ***C***(4'b)), 86.5 (1C, s, ***C***-(DMTr)a), 86.6 (1C, s, ***C***-(DMTr)b), 87.2 (1C, s, ***C***(1' a)), 88.1 (1C, s, ***C***(1' b)), 102.6 (1C, s, ***C***(5)), 113.7 (4C, s, ***C***H-(DMTr)), 119.1 (1C, s, -***C***N a), 119.3 (1C, s, -***C***N b), 121.6 (1C, d, J = 25561 Hz, -***C***F3), 127.3 (1C, s, ***C***H-(DMTr)), 128.2 (2C, s, ***C***H-(DMTr)), 128.4 (2C, s, ***C***H-(DMTr)), 130.3 (4C, s, ***C***H-(DMTr)), 135.4 (1C, d, J=21.7 Hz, ***C***-(DMTr)), 135.5 (1C, d,J=16.9 Hz, ***C***-(DMTr)), 140.8 (1C, s, ***C***(6)a), 141.1 (1C, s, ***C***(6)b), 144.9 (1C, s, ***C***-(DMTr)a), 145.0 (1C, s, ***C***-(DMTr)b), 150.7 (1C, s, ***C***(2)), 158.7 (2C, s, ***C***-DMTr), 163.4 (1C, s, ***C***(4)). ^{19}F-NMR (376 MHz, DMSO-d6, 25°C, δ [ppm]): δ = -57.15 (3F, d, J=3.49, ***F*3**C-) & -57.25 (3F, d, J=5.20, ***F*3**C-). ^{31}P-NMR (162 MHz, DMSO-*d*6, 25 °C, δ [ppm]): δ = 150.51 (1P, s, ***P***-OC(3')) & 149.78 (1P, duplettoid, J=5.84 Hz, ***P***-OC(3')).

^{1}H-NMR (400 MHz, DMSO-d_6, 25 °C) of compound **U3**

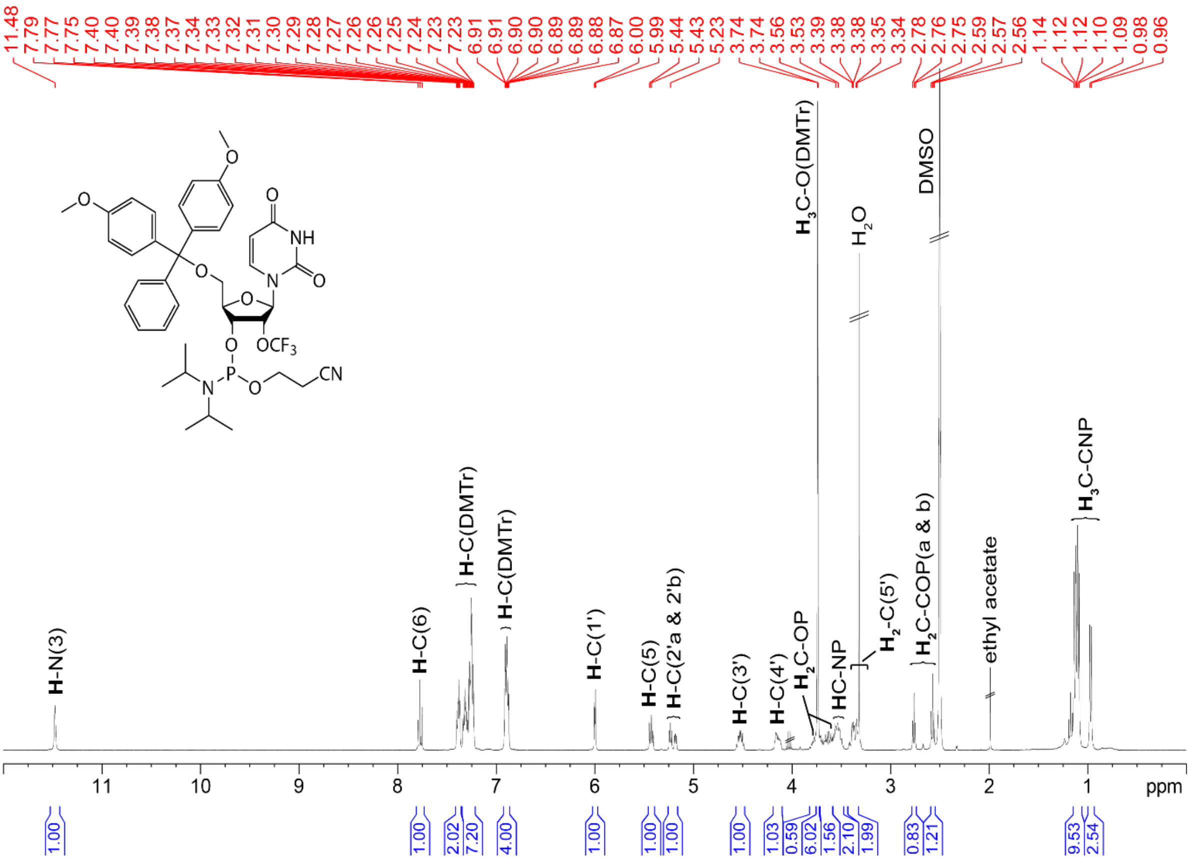

^{13}C-NMR (100 MHz, DMSO-d_6, 25 °C) of compound **U3**

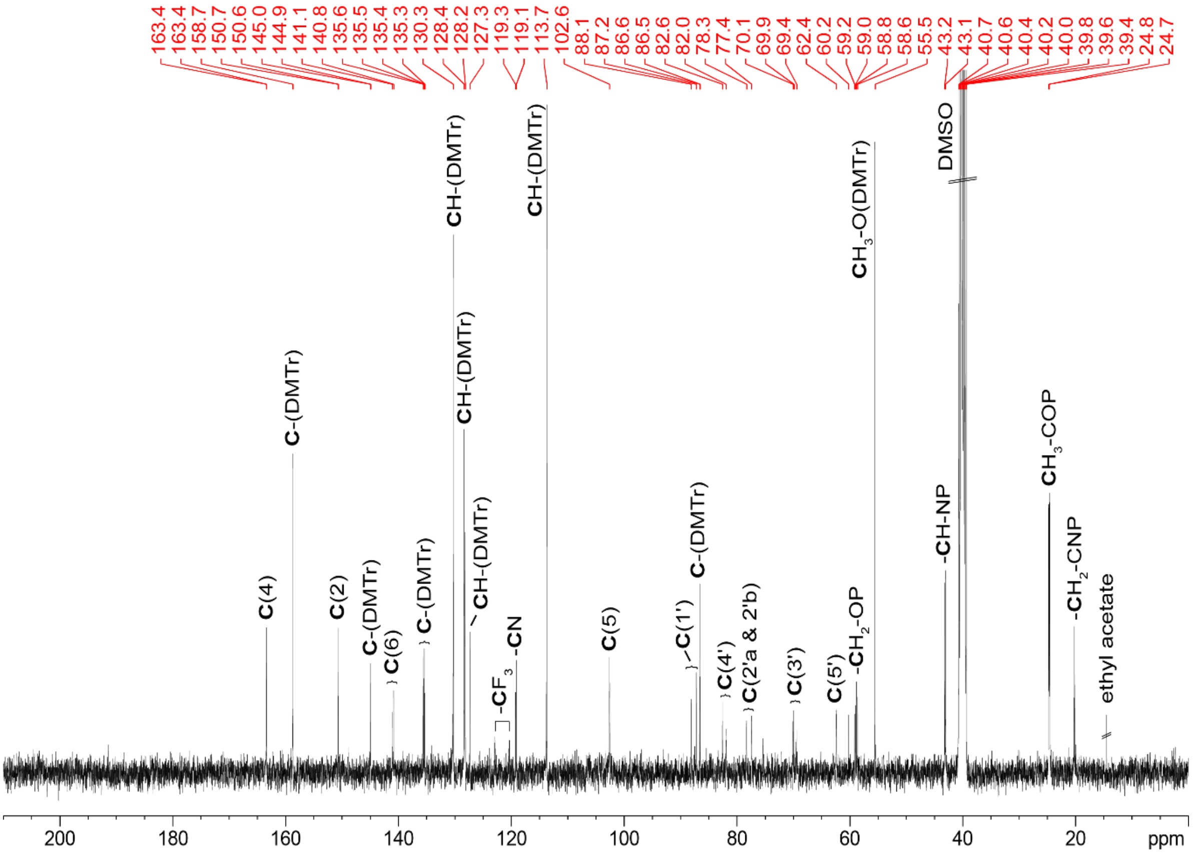

^{19}F-NMR (377 MHz, DMSO-d_6, 25 °C) of compound **U3**

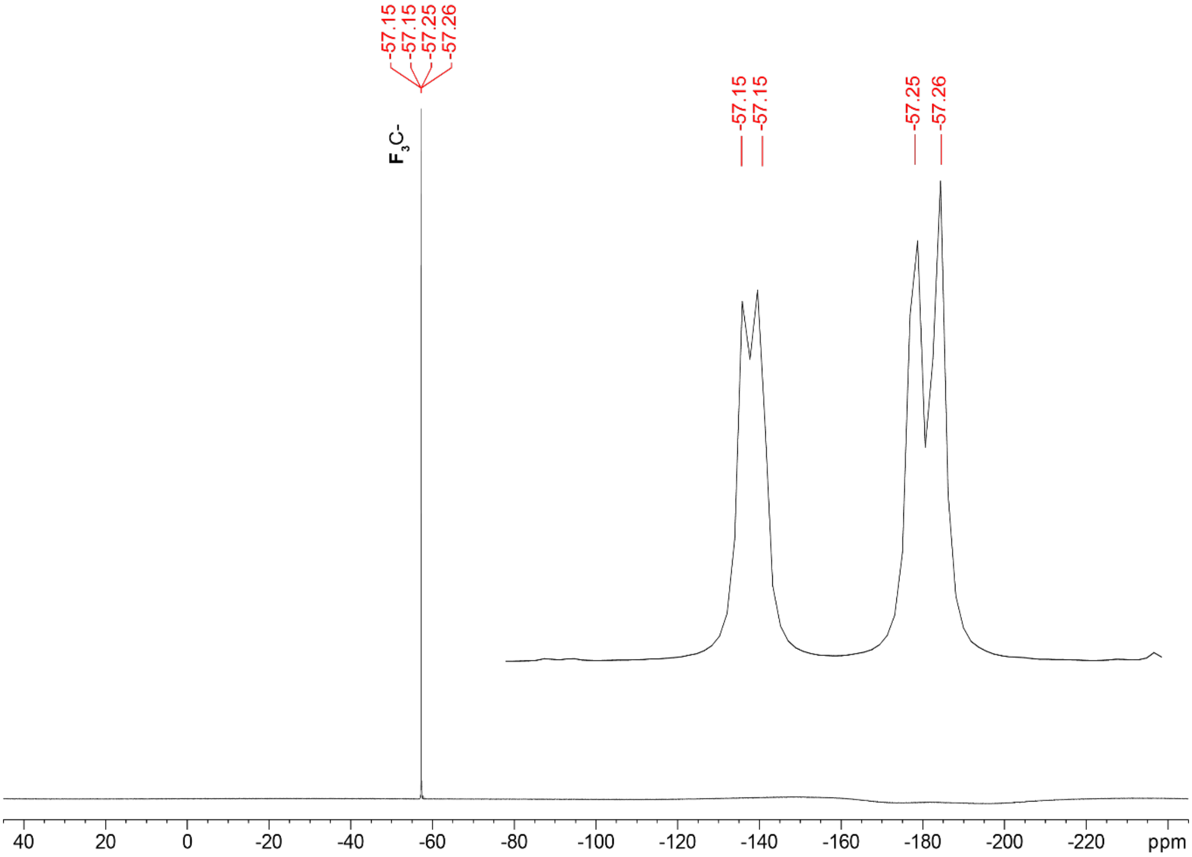

^{31}P-NMR (162 MHz, DMSO-d_6, 25 °C) of compound **U3**

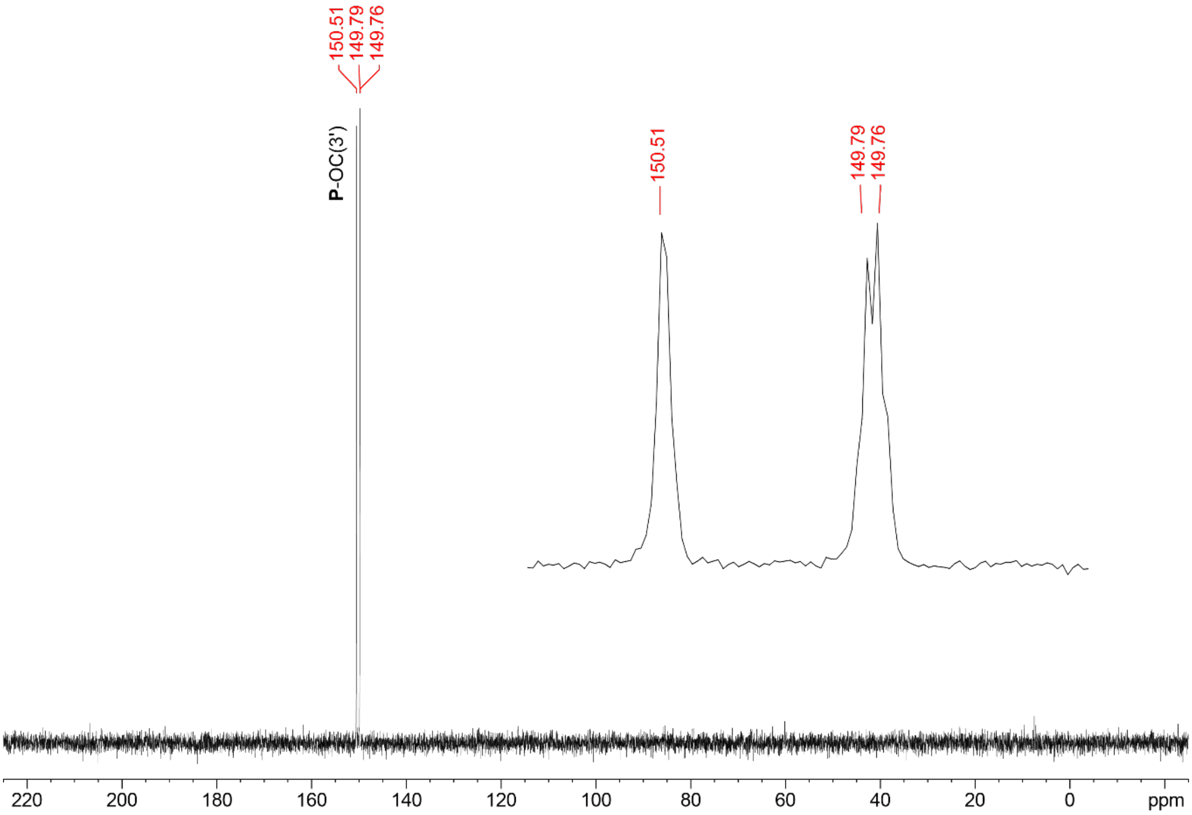

Table S1. Selection of synthesized 2'-OCF_3 modified RNAs.

Sequence (5' → 3')	nt	Molecular weight	
		calc.	found
GGC**U**$^{2'-OCF3}$AGCC	8	2592.6	2592.4
GGUC**G**$^{2'-OCF3}$ACC	8	2592.6	2592.6
GGCA**G**$^{2'-OCF3}$AGGC	9	3000.9	3000.8
GCCU**U**$^{2'-OCF3}$UGCC	9	2835.7	2835.7
GAA**G**$^{2'-OCF3}$GGCAACCUUCG	15	4882.0	4882.1
GAAGGGCAACC**U**$^{2'-OCF3}$UCG	15	4882.0	4881.8
UGUCU**U**$^{2'-OCF3}$AUUGGCAGAGACCUdG	21	6741.9	6741.9
UGUCU**U**$^{2'-OCF3}$AU**U**$^{2'-OCF3}$GGCAGAGACCUdG	21	6809.9	6809.8
UGUCUUAU**U**$^{2'-OCF3}$GGCAGAGACCUdG	21	6741.9	6741.8
CUGGGUCGCA**G**$^{2'-OCF3}$UAACCCCAGUUAACAAAACAAG	33	10650.5	10651.0
CUGGGUCGCAGUAACCCCAGUUAACAAAACAAG**G**$^{2'-OCF3}$G	35	11340.9	11341.3
AUUCCUC**U**$^{2'-OCF3}$UCAUCCAUACAGACAGAACUAACGAUUCG	37	11756.1	11755.8

Table S2. List of 2'-OCF_3-modified siRNA duplexes used in this study

No.	siRNA sense and antisense sequences	
siRNA-U6	5′-GGUCUCUGCCAAUA----AGACAUT-3'	sense
	3'-dGUCCAGAGACGGUUAU^{OCF3}UCUGU-5'	antisense
siRNA-U9	5′-GGUCUCUGCCA----AUAAGACAUT-3'	sense
	3'-dGUCCAGAGACGGU^{OCF3}UAUUCUGU-5'	antisense
siRNA-U6/U9	5′-GGUCUCUGCCA----AUA----AGACAUT-3'	sense
	3'-dGUCCAGAGACGGU^{OCF3}UAU^{OCF3}UCUGUUCUGU-5'	antisense
siRNA unmodified	5′-GGUCUCUGCCAAUAAGACAUT-3'	sense
	3'-dGUCCAGAGACGGUUAUUCUGU-5'	antisense
siRNA random	5′-GGUCUCUGCCAAAAGGACAUT-3'	sense
	3'-dGUCCAGAGACGGUUUUCCUGU-5'	antisense

References

[1] M. Aigner, M. Hartl, K. Fauster, J. Steger, K. Bister, R. Micura, *Chembiochem*, **2011**, *12*, 47-51.

[2] M. Hartl, K. Puglisi, A. Nist, P. Raffeiner, K. Bister, *Mol Oncol.* **2020**, *14*, 625-644.
